# Genome-wide survey and expression analysis of calcium-dependent protein kinase (CDPK) in grass *Brachypodium distachyon*

**DOI:** 10.1186/s12864-020-6475-6

**Published:** 2020-01-16

**Authors:** Feng Wen, Feng Ye, Zhulong Xiao, Liang Liao, Tongjian Li, Mingliang Jia, Xinsheng Liu, Xiaozhu Wu

**Affiliations:** School of Pharmacy and Life Science, Jiujiang University, Jiujiang, China

**Keywords:** CDPK, Phytohormones, Abiotic stresses, Expression pattern, *Brachypodium distachyon*

## Abstract

**Background:**

Ca^2+^ played as a ubiquitous secondary messenger involved in plant growth, development, and responses to various environmental stimuli. Calcium-dependent protein kinases (CDPK) were important Ca^2+^ sensors, which could directly translate Ca^2+^ signals into downstream phosphorylation signals. Considering the importance of CDPKs as Ca^2+^ effectors for regulation of plant stress tolerance and few studies on *Brachypodium distachyon* were available, it was of interest for us to isolate CDPKs from *B. distachyon*.

**Results:**

A systemic analysis of 30 CDPK family genes in *B. distachyon* was performed. Results showed that all BdCDPK family members contained conserved catalytic Ser/Thr protein kinase domain, autoinhibitory domain, and EF-hand domain, and a variable N-terminal domain, could be divided into four subgroup (I-IV), based upon sequence homology. Most BdCDPKs had four EF-hands, in which EF2 and EF4 revealed high variability and strong divergence from EF-hand in AtCDPKs. Synteny results indicated that large number of syntenic relationship events existed between rice and *B. distachyon*, implying their high conservation. Expression profiles indicated that most of *BdCDPK* genes were involved in phytohormones signal transduction pathways and regulated physiological process in responding to multiple environmental stresses. Moreover, the co-expression network implied that *BdCDPKs* might be both the activator and the repressor involved in WRKY transcription factors or MAPK cascade genes mediated stress response processes, base on their complex regulatory network.

**Conclusions:**

BdCDPKs might play multiple function in WRKY or MAPK mediated abiotic stresses response and phytohormone signaling transduction in *B. distachyon*. Our genomics analysis of BdCDPKs could provide fundamental information for further investigation the functions of CDPKs in integrating Ca^2+^ signalling pathways in response to environments stresses in *B. distachyon*.

## Background

To adapt the environmental stresses, such as drought, cold, heat and high salinity, plant developed a series of effective defense mechanism comprised of stress signal perception and subsequent transduction, leading to the activation of various physiological and metabolic responses [1]. As a ubiquitous secondary messenger, Ca^2+^ played an important role in the signal transduction pathways via transiently changing in the cytoplasmic Ca^2+^ concentration in plant growth and development, as well as responses to various environmental stimuli [2–4]. During exposure of plants to stress, several Ca^2+^ sensors or Ca^2+^ binding proteins can sense Ca^2+^ concentration changes and recognize the Ca^2+^ signatures, and then subsequently transduct them into a series of downstream effects to improve plant resistance [5, 6]. Up to present, four major classes of Ca^2+^ sensors or Ca^2+^ binding proteins, including calmodulins (CaM), calmodulin-like proteins (CaML), calcineurin B-like proteins (CBL) and calcium-dependent protein kinases (CDPK), have been indentified in plants [7–10]. Among these, CDPKs were distinctive because their unique structure, which contains a catalytic Ser/Thr protein kinase domain and a CaM domain containing EF-hand motifs for Ca^2+^-binding capacity [11]. Therefore, CDPK proteins can function both as Ca^2+^ sensors and effectors to directly translate Ca^2+^ signals into downstream phosphorylation signals [12, 13].

CDPKs, which were plant-specific gene family, have been identified throughout the plant kingdom, but not in animals [6, 14, 15]. Typical CDPK gene family members were composed of four conserved domains, including a variable N-terminal domain, a protein kinase domain, an auto-inhibitory domain and a C-terminal regulatory calmodulin-like domain [6]. The N-terminal domains were consisting of the myristoylation and palmitoylation sites, which helped CDPKs anchor to or/and dissociate from membranes, which were highest sequence divergence among CDPKs [16]. The different lengths and amino acid compositions of the N-terminal domain led to specific function of the individual CDPKs [16, 17]. The protein kinase domain contains a catalytic Ser/Thr protein kinase domain with a ATP binding site and is adjacent to the auto-inhibitory domain, acting as a pseudosubstrate combined with kinase domain to maintain CDPK inactive [18, 19]. The C-terminal regulatory calmodulin-like domain contained one to four EF-hand motifs for Ca^2+^ binding, which was the center of Ca^2+^ sensor. The Ca^2+^ binding led to a change in the protein structure, altered the auto-inhibitory domain, and then activated the CDPKs [20].

A number of studies from various plant species confirmed that CDPK genes played important roles in regulating plant growth and response to various stimuli, including light, hormones, wounding, abiotic and biotic stresses [9, 21–23]. As for the critical roles that CDPKs played in Ca^2+^ signaling transduction, many studies presented their functions in plant growth and development, including pollen tube growth, nutrient transport, seed development and germination, stem and petiole elongation. For example, the anatomical analysis of *Atcpk28* mutant has been revealed that AtCPK28 acted as a developmentally controlled regulator for coordinated stem and petiole elongation, and vascular development [24]. Besides, AtCPK17 and AtCPK34 were found earlier to be essential to pollen fitness, and transduce Ca^2+^ signals to increase the rate of pollen tube tip growth and facilitate a response to tropism cues [25]. In rice, OsCDPK2 and OsCDPK11 have been reported to be essential for seed development, while further study revealed that over-expression *OsCDPK2* could disrupt seed development [26, 27]. Multiple researches proved that CDPKs were involved in response to environmental stress stimuli in conjunction with ABA signaling transduction [12, 16, 28]. In Arabidopsis, CPK10, possibly by interacting with HSP1, was involved in Ca^2+^-mediated regulation of stomatal movements under drought treatment and played important roles in ABA signaling [29]. Loss-of-function mutations of CPK4 and CPK11 exhibited pleiotropic ABA-insensitive phenotypes and decreased tolerance of seedlings to salt stress, further study showed that CPK4 and CPK11 positively regulated calcium-mediated ABA signaling pathways through the phosphorylation of two ABA-responsive transcription factors, ABF1 and ABF4 [28]. Over-expression of *OsCDPK7* enhanced plant tolerance to salinity/drought by inducting some stress-responsive genes, but not to cold, while OsCDPK13 and OsCPK17 were supposed to be important signaling components for response to cold stress in rice [30–32]. Recent studies have provided compelling evidence for the involvement of CDPKs in immunity signaling and plant disease resistance. CDPKs could act synergistically or independently with the MAPK cascade in regulating PAMP-triggered immunity signaling, or phosphorylate distinct substrates in regulating effector-triggered immunity signaling, via phosphorylating WRKY transcription factors for immune gene expression and Rbohs for ROS production. For example, AtCDPK4/5/6/11 could regulate MAMP- or PAMP-triggered immunity and phosphorylate WRKY transcription factors to regulate gene expression [33]. Moreover, although most of CDPKs exhibited positive roles in regulating plant growth and response to environmental stimuli, some CDPKs played negative roles. For instance, *atcpk21* and *atcpk23* mutants showed increased tolerance to drought and salt stresses, suggesting that AtCPK21 and AtCPK23 acted as negative regulators in *Arabidopsis* responses to abiotic resistance [34, 35] Interesting, AtCPK12 might be a balancer in ABA signaling pathway, since AtCPK12 could phosphorylate ABF1 and ABF4 as well as ABI2 [36, 37].

As a critical role of the Ca^2+^-signaling pathway, CDPKs were encoded by a large gene family in various plant species. To date, genome-wide analyses have identified 34 *CDPK* genes in Arabidopsis [9, 18], 31 *CDPK* genes in rice [38], 20 *CDPK* genes in wheat [39], 35 *CDPK* genes in maize [40], and 41 *CDPK* genes in cotton [41]. *Brachypodium distachyon*, which has been whole genome sequenced, became a attractive model organism for crops and herbaceous functional genomics research [42–44]. Considering the importance of *CDPK* genes as promising candidates for regulation of plant stress tolerance and few studies on *B. distachyon* were available, it was of interest for us to isolate CDPKs from *B. distachyon*. In this study, a total 30 *CDPK* family genes were identified from Bd21 genome, and the gene characterizations and phylogenies have been systematically analyzed. Furthermore, an expression heatmap of *BdCDPKs* in response to different hormones and abiotic stresses was also exhibited, a predicted co-expression network was also discussed. The identification and systematical study for *CDPK* genes from *B. distachyon* will provide fundamental information for exploring the functions of CDPKs in integrating Ca^2+^ signalling pathways in *B. distachyon*’s adaptation to vagaries of environments.

## Results and discussion

### Identification of 30 *BdCDPK* genes from the *B. distachyon* plants

**Fig. 1:**
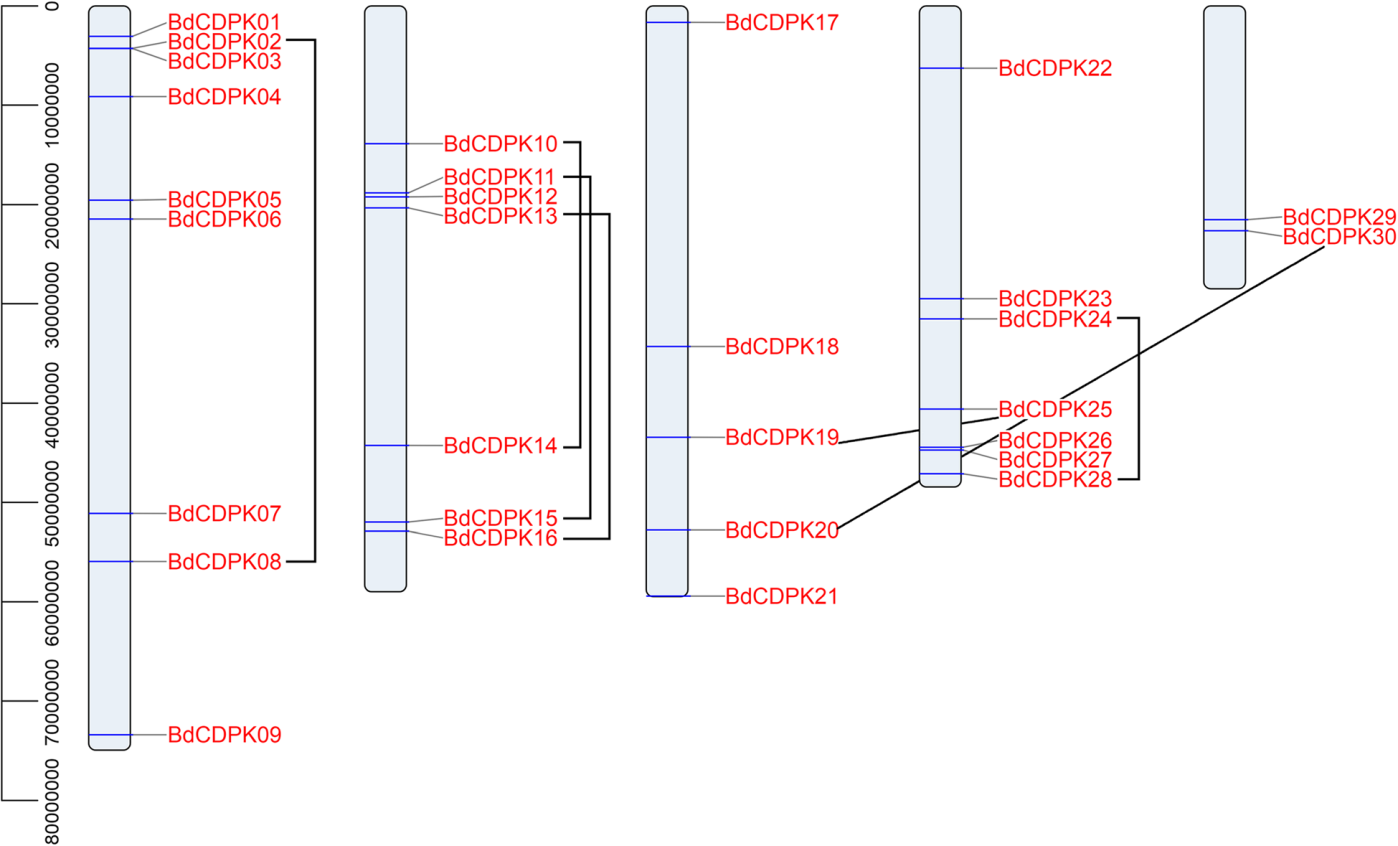
Chromosome distribution of *CDPK* genes in *B. distachyon*. The chromosome numbers are indicated at the top of each chromosome image. Gene duplication analysis of *BdCDPKs* was also presented with a gray line

A total of 30 *CDPK* family genes were identified in *B. distachyon*, using known rice and Arabidopsis CDPK proteins as query sequences (Table 1 and Additional file 6) [9, 38]. We named these genes *BdCDPK1* to *BdCDPK30* according to the position of the genes on chromosomes. The detailed gene information for these *BdCDPK* genes, such as gene names, Locus IDs, gene locations, peptide lengths, conserved domain and site locations, and parameters for the deduced polypeptides, are listed in Table 1. The BdCDPKs identified in our study varied markedly from 508 amino acids (BdCDPK18) to 623 amino acids (BdCDPK03), ranged in molecular mass from 56.8 kDa to 68.7 kDa, and the predicted isoelectric points varied from 4.94 (BdCDPK28) to 9.35 (BdCDPK04), which were comparable with CDPKs from other plant species [9, 38, 45]. The result of subcellular localization prediction showed that most of BdCDPKs were located in cytoplasmic, other of them were predicted to exit in chloroplast or mitochondrial, which was consistent with the fact that CDPKs acted as Ca^2+^ sensors to integrate extracellular stimuli [46]. The 30 identified *BdCDPK* genes were distributed on all five chromosomes (Fig. 1). The number of *BdCDPK* genes mapped on each chromosome was uneven and ranged from 2 to 9. Ten *BdCDPK* genes were present on chromosome 1, representing 33.3% of the total *CDPK* genes, followed by seven each on chromosomes 2 and 4, five on chromosomes 3, and two on chromosomes 5 (Table 1 and Fig. 1).

**Table 1:** CDPK genes in *B. distachyon*

Gene Name	Gene Locus	Genebank Acc. No.	ORF (bp)	Exon No.	Pepide length	PKD	EF-hand domain	PI	MW (KD)	N- myrist^a^	N- Palmit^b^	Subcellular Localization^c^
BdCDPK01	Bradi1g04440	XM_010229652	1602	8	533	58–316	366–533	6.31	59.96	N	Y	pero: 6, mito: 5, chlo: 2
BdCDPK02	Bradi1g06270	XM_010230428	1773	7	590	127–385	435–590	5.29	64.59	N	Y	vacu: 6, chlo: 4, nucl: 2, pero: 1
BdCDPK03	Bradi1g06300	XM_024456669	1872	7	623	157–415	465–623	5.84	68.48	N	Y	chlo: 6, vacu: 6, nucl: 1
BdCDPK04	Bradi1g12150	XM_003559516	1863	6	620	109–405	455–620	9.35	68.74	N	Y	chlo: 6, vacu: 3, E.R.: 2, nucl: 1.5, cysk_nucl: 1.5
BdCDPK05	Bradi1g24240	XM_003562825	1617	8	538	64–322	372–538	6.98	60.5	N	Y	cyto: 7, pero: 3, E.R.: 2, plas: 1
BdCDPK06	Bradi1g26310	XM_024456613	1599	8	532	84–342	391–532	5.8	59.38	N	Y	chlo: 8, cyto: 3, nucl: 2
BdCDPK07	Bradi1g52567	XM_003557252	1551	12	516	53–313	363–516	7.63	57.85	N	Y	cysk: 7, cyto: 3, chlo: 1, nucl: 1, plas: 1
BdCDPK08	Bradi1g56970	XM_010230066	1704	7	567	105–363	413–567	5.17	62.42	N	Y	E.R.: 5, chlo: 2, nucl: 2, cyto: 1, plas: 1, vacu: 1, pero: 1
BdCDPK09	Bradi1g76560	XM_024456675	1653	7	550	80–339	389–550	5.45	61.51	N	Y	nucl: 3.5, cyto_nucl: 3.5, mito: 3, plas: 3, cyto: 2.5, chlo: 2
BdCDPK10	Bradi2g15520	XM_003567783	1647	8	548	96–354	404–548	6.56	62.12	Y	N	chlo: 5, nucl: 5, pero: 3
BdCDPK11	Bradi2g21390	XM_003568155	1551	7	516	62–320	370–516	5.59	56.94	N	Y	chlo: 3, plas: 3, vacu: 2, E.R.: 2, nucl: 1.5, cysk_nucl: 1.5
BdCDPK12	Bradi2g21790	XM_003566058	1575	7	524	13–298	364–524	5.33	58	N	N	chlo: 6, cyto: 4, nucl: 3.5, nucl_plas: 2.5
BdCDPK13	Bradi2g22750	XM_003568218	1647	7	548	74–332	382–548	6.25	60.79	N	Y	cyto: 4, mito: 3, chlo: 2, E.R.: 2, pero: 1, cysk: 1
BdCDPK14	Bradi2g43910	XM_003569338	1581	8	526	74–332	382–526	5.8	59.44	Y	N	mito: 8, chlo: 4, nucl: 1
BdCDPK15	Bradi2g52870	XM_003564390	1545	6	514	59–317	367–514	5.45	56.78	N	Y	chlo: 4, plas: 3, nucl: 2.5, cyto: 2, cysk_nucl: 2, vacu: 1
BdCDPK16	Bradi2g54080	XM_024458897	1686	6	561	62–320	370–561	6.49	63.26	N	Y	chlo: 4, cyto: 4, mito: 2, E.R.: 1, pero: 1, cysk: 1
BdCDPK17	Bradi3g02600	XM_010235446	1563	12	520	57–317	367–520	8.99	58.64	Y	Y	cyto: 7, chlo: 2, nucl: 2, mito: 2
BdCDPK18	Bradi3g32187	XM_003571966	1527	6	508	66–324	374–508	5.23	57.16	N	Y	mito: 4, nucl: 3, chlo: 2, cyto: 2, plas: 1, pero: 1
BdCDPK19	Bradi3g41770	XM_003572421	1740	3	579	78–368	418–579	6.5	63.84	Y	Y	chlo: 5, cyto: 2, mito: 2, nucl: 1, vacu: 1, E.R.: 1, cysk: 1
BdCDPK20	Bradi3g51970	XM_003570070	1671	7	556	98–356	406–556	5.45	56.78	Y	Y	mito: 10.5, chlo_mito: 7.5, chlo: 3.5
BdCDPK21	Bradi3g60750	XM_003570682	1599	2	532	57–325	375–532	5.13	58.5	Y	Y	cyto: 7, mito: 4, nucl: 2
BdCDPK22	Bradi4g07280	XM_010240643	1833	6	610	158–416	466–610	5.92	67.78	Y	Y	chlo: 5, mito: 3.5, cyto_mito: 2.5, cyto: 2, nucl: 1, E.R.: 1
BdCDPK23	Bradi4g24390	XM_003577735	1557	7	518	52–310	360–518	5.28	57.05	N	N	chlo: 11, E.R.: 2
BdCDPK24	Bradi4g26317	XM_003576298	1599	5	532	75–333	383–532	5.6	58.44	Y	Y	cyto: 7, chlo: 4, nucl: 2
BdCDPK25	Bradi4g35100	XM_003578386	1698	8	565	88–358	426–565	5.95	62.54	Y	Y	chlo: 3, mito: 3, cyto: 2, plas: 2, vacu: 2, nucl: 1
BdCDPK26	Bradi4g39870	XM_003578709	1713	6	570	81–339	389–570	6.14	63.3	N	Y	chlo: 6, mito: 6, cyto: 1
BdCDPK27	Bradi4g40300	XM_003578741	1572	7	523	48–306	356–523	5.53	57.65	N	N	chlo: 7, E.R.: 3, plas: 2, nucl: 1
BdCDPK28	Bradi4g43400	XM_003578916	1650	6	549	91–349	399–549	4.94	60.07	N	Y	cyto: 10, nucl: 3
BdCDPK29	Bradi5g18250	XM_010241951	1719	7	572	99–388	437–572	6.6	64.05	Y	Y	cyto: 7, chlo: 4, nucl: 2
BdCDPK30	Bradi5g19430	XM_003580349	1686	7	561	98–356	406–561	5.49	61.59	Y	Y	mito: 9.5, chlo_mito: 7, chlo: 3.5

*ORF* Open Reading Frame, *PKD* Protein kinase domain, *PI* isoelectric point

a. The myristoylation site was predicted by Myristoylator program in ExPASy (http://web.expasy.org/myristoylator/)

b. The palmitoylation site was predicted by CSS-plam4.0 (http://csspalm.biocuckoo.org/)

c. *chlo* Ch**l**oroplast, *cysk* Cytoskeleton, *cyto* Cytoplasmic, *E.R.* Endoplasmic reticulum, *mito* Mitochondrial, *nucl* Nuclear, *pero* Peroxysome, *plas* Plasma membrane, *vacu* Vacuole

To dissect the evolutionary relationships of BdCDPK family members, all BdCDPK full-length amino acid sequences were used to construct an unrooted tree (Additional file 7). Based upon sequence homology, BdCDPK family members were clustered into four subgroup (I-IV), which was consistent with the previous works in Arabidopsis, rice, maize, and so on [9, 38, 40, 47]. Subgroup IV contained the least members, with three members, in which, the BdCDPK12 showed distant relationship with other members in this subgroup. Subgroup I was the most complex, with 11 members, while Subgroups II and III contained eight members each. Subgroups I-III showed more closer in sequence identity to each other than to subgroup IV, but it remained unknown whether such a pattern of clustering reflected any functional differences among these subgroups.

### Structural divergence and tissue-specific expression of *BdCDPKs*

**Fig. 2:**
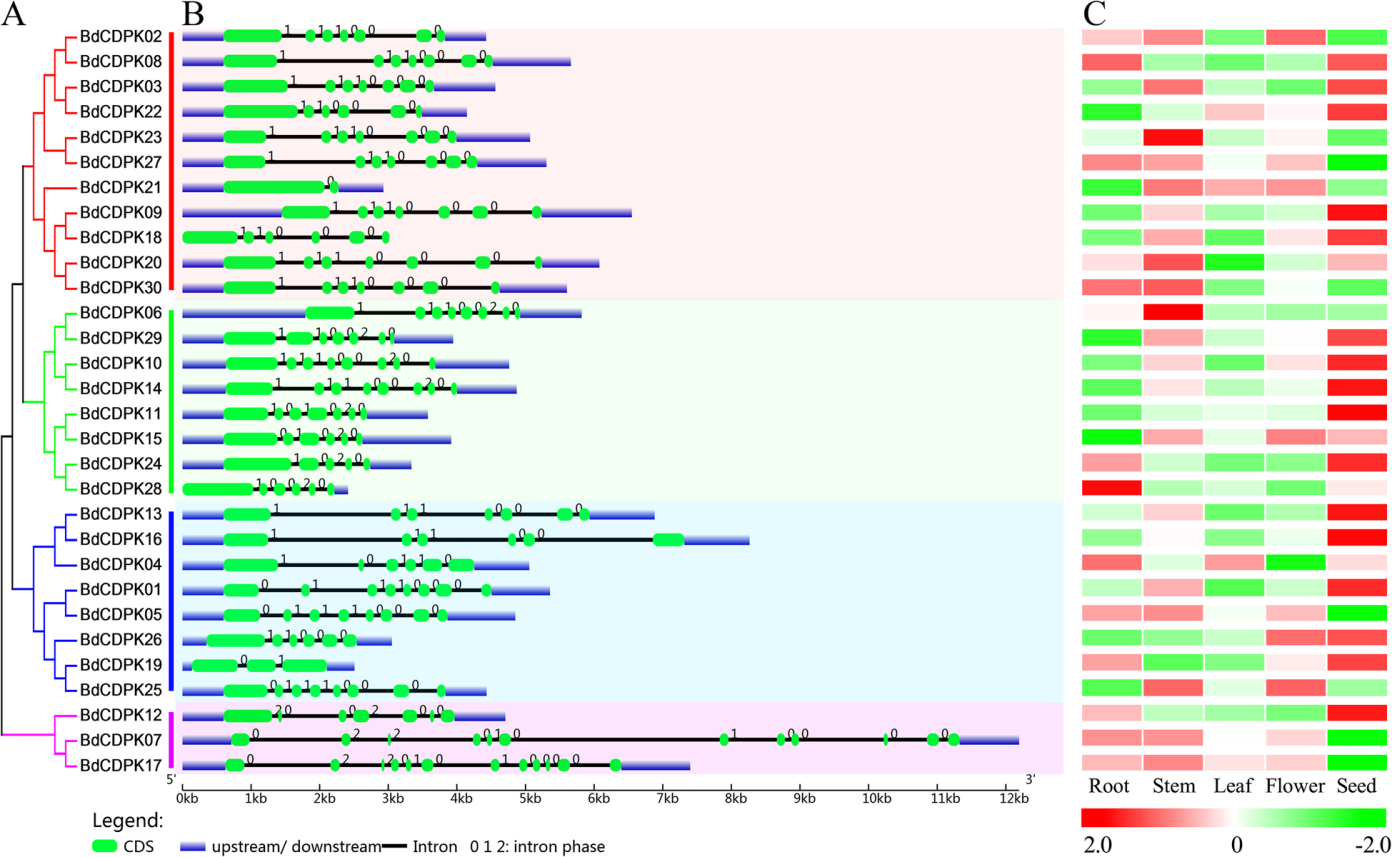
Gene structure and tissue-specific expression heatmap of *BdCDPKs*. **a** Unrooted phylogenetic relationships among the BdCDPK protein sequences. Gene classes were indicated with different colors. **b** Exon-intron organization of corresponding *BdCDPK* gene. The exons and introns are represented by boxes and lines, respectively. **c** Analysis of the *CDPK* genes in different tissues of *B. distachyon*. Heatmap representation and hierarchical clustering of *BdCDPK* genes in root, stem, and leaf

The gene structure provides information of possible structural evolutionary relationships among gene families. To gain further insight into the structural diversity of *BdCDPK* genes, we constructed a separate unrooted phylogenetic tree using the full-length BdCDPK protein sequences, and compared it with the exon/intron organization of the corresponding gene sequence (Fig. 2). As reflected by the evolutionary relationship of these CDPK proteins, most of the BdCDPK members clustered in the same subfamily shared very similar exon-intron structures, including intron numbers and exon/intron lengths, and although the lengths varied, the number and/or locations of intron insertions were seemed similar. Most *BdCDPK* genes from subgroup I possessed seven exons, except for *BdCDPK18/22* and *BdCDPK21*, which contained six and two exons, respectively. Most genes in subgroup II and III had 6–8 exons, subgroup IV possessed 12 exons except *BdCDPK12*, these traits are also found in Arabidopsis and rice [38, 45]. Since genes in the joint phylogenetic tree were considered the related sister pairs and triplets [48, 49], fourteen related sister pairs among the *BdCDPK* gene family, were displayed very similar intron/exon scatter in terms of intron/exon length and number. This conserved exon/intron structure in each group and sister gene pairs, supported the close evolutionary relationship of these CDPKs and the introduced classification of groups. Other *BdCDPK* genes were showed divergent gene structure, suggesting that the function of these genes might be diversified.

Extensive studies have proved that the function of gene was often correlated with its transcriptional profile. Previous findings have suggested that the *CDPK* gene family was involved in plant growth and development [23, 50, 51]. Since no CDPKs in *B. distachyon* has been previously documented, and to investigate the divergence of homologs and putative functions of *CDPK* genes in *B. distachyon* growth and development, we analyzed the expression patterns of *CDPK* genes in different tissues. The results showed high alterations in expression levels among different CDPK group genes in *B. distachyon*, and a similar expression pattern in same CDPK group genes. Most paralogous genes which clustered as sister branches in the phylogenetic tree (Fig. 2a) and shared sequence identities no less than 90% presented largely similar expression patterns (Fig. 2c), implying their functional redundancy. Notably, *BdCDPK09* and *BdCDPK18*, *BdCDPK03* and *BdCDPK22*, *BdCDPK10* and *BdCDPK14*, and *BdCDPK13* and *BdCDPK16* were almost exclusively expressed in seed, while *BdCDPK07* and *BdCDPK17* were lowest in seed, but highly expressed in root and stem, suggesting that they might be involved in these organs’ development and growth. However, some paralogous exhibited rather different expression patterns. For instance, *BdCDPK08* and *BdCDPK19* showed high expression level in root and seed, whereas their paralogs (*BdCDPK02* and *BdCDPK25*, respectively) appeared to have relatively high expression in stem and flower. These data indicate that the *CDPK* gene family members might be involved in the growth and development of different tissues or organs.

**Fig. 3:**
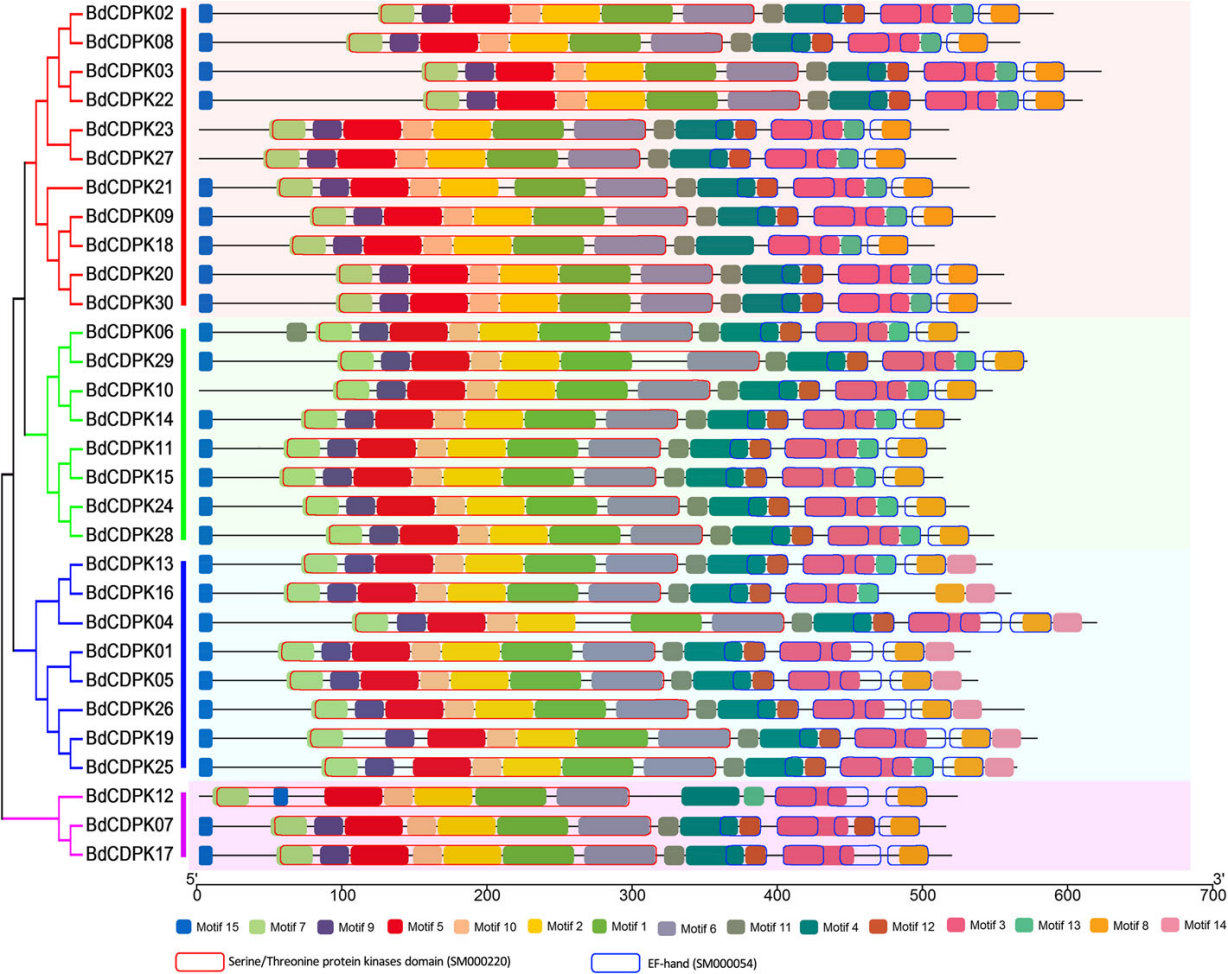
Protein structures of BdCDPKs in *B. distachyon*. Different motif is represented by specific color. Red hollow box indicted the Serine/Threonine protein kinases domain (SM000220), and blue hollow box indicted the EF-hand (SM000054)

To investigate the protein structural and functional diversity of BdCDPKs, motif and domain analyses were performed based on the phylogenetic relationship (Table 1 and Fig. 3). Generally, 15 conserved motifs within the *B. distachyon* CDPKs were identified using online MEME tools, which can help us investigate their function [52]. As mentioned above, the unrooted phylogenetic tree showed that BdCDPKs were broadly divided into four major subgroups. Eight of the motifs (motif 7, 9, 5, 10, 2, 1, 6, and 4) were shared by all of the CDPK proteins. Meanwhile, the conserved gene structures revealed similar motifs among groups. For example, motif 14 was only presence in subgroup III, while most of this subgroup members did not contain motif 13. The motif analysis results illustrated that conserved motif structures within each group supported their close evolutionary relationship, while there might be functional divergences between different groups. Further, the protein structure of BdCDPKs were predicting using InterPro and SMART protein databases and sequence alignment (Fig. 3 and Additional files 8, 9 and 10). Results showed that all CDPKs identified in our study were possessed of four characterized domains, including a variable N-terminal domain, a catalytic Ser/Thr protein kinase domain, an autoinhibitory domain, and a EF-hand domain, which were considered as the typical CDPK structure [19].

The variable N-terminal domain of several CDPK proteins had myristoylation motif with an N-terminal glycine residue, which was thought to be critical for mediating protein-protein and protein-lipid interactions. Myristoylation played an essential role in membrane targeting and functioned widely in a variety of signal transduction pathways [53, 54]. As a kind of important Ca^2+^ sensor, CDPKs had an N-myristoylation motif tended to localize in the plasma membranes, suggesting that myristoylation was required for membrane binding and Ca^2+^ signal transduction pathways [29, 39, 55–57]. In addition, another type of lipid modification, palmitoylation, could enhance the hydrophobicity of proteins and contributes to their membrane association, playing a significant role in subcellular trafficking of proteins between membrane compartment [55, 58]. Among 30 BdCDPKs, 11 CDPKs were predicted to have myristoylation motifs, and 5 members lacked palmitoylation site at the N-terminus (Table 1). Exceptionally, the membrane association of CDPKs might be affected by other motifs. For instance, TaCPK3 and 15, lacking myristoylation motifs, were also shown to be membrane-bounded, lacked myristoylation motifs [39], while a ZmCK1::hGFP fusion protein was found to localize to the cytoplasm and nucleus, which was predicted to have an N-myristoylation [59]. Therefore, other motifs may affect the subcellular localizations, and it was still needed to be further characterized experimentally.

All members of BdCDPK contained a protein kinase domain (Interpro acc. no. IPR000719, SMART acc. no. SM000220), which was composed of motifs 7, 9, 5, 10, 2, 1, and 6 as shown in Fig. 3. In the protein kinase domain, it contained motif 7 and 2 as ATP binding site (IPR017441) and serine/threonine-protein kinase active site (IPR008271), respectively. The protein kinases domain could catalyze the gamma phosphate transferring to amino acid residues (such as serine and threonine) in a protein substrate side chain from nucleotide triphosphates (often ATP), resulting in a conformational change affecting protein function [60]. All members of BdCDPKs contained an ATP-binding site, suggesting that these BdCDPKs could used ATP as source of phosphate groups to phosphorylate target substrates in signal transduction pathways. Although the protein kinases domain is extremely conservation, we notice that the sequences of serine/threonine-protein kinase active site among different subgroups contain a large number of variations. For example, the sequences of protein kinase active site in subgroup III was quite different with subgroups I and II (Additional file 9). Similarly, the residues in protein kinase active site of subgroup IV, especially BdCDPK12, were less conservative compared with subgroups I – III.

The calmodulin-like domain contained Ca^2+^ binding EF-hand structure that made CDPK proteins to function as a Ca^2+^ sensor. Each EF-hand structure consists of a helix-loop-helix structure, and at least one Ca^2+^-binding site composed of 12 amino acids confers the Ca^2+^-binding activity [61]. In *B. distachyon*, 26 CDPKs had four Ca^2+^ binding EF-hand structures (Interpro acc. no. IPR002048, SMART acc. no. SM000054), which might be composed of motif 4, 12, 3, 13, and 8, four CDPKs (BdCDPK05, 12, 16, and 18) contained three EF-hand structures each (Fig. 3 and Additional files 8, 9 and 10). This difference in EF-hand structure was also found in the CDPK family of other plants [9, 38, 40, 62]. Further, the structure of EF-hands in *B. distachyon* were D_1_-D_3_-S_5_-E_12_, D_1_-D_3_-S/N_5_-E_12_, D_1_-D_3_-S_5_-E_12_, and D_1_-D_3_-D_5_-E_12_, respectively (Additional file 10). And results showed that EF2 and EF4 exhibited difference with CDPKs in Arabidopsis [63]. Studies have proved that the C-terminal EF-hand structures might be important for recognizing the autoinhibitory-junction domain when the Ca^2+^ regulated the activity of CDPKs [60, 64, 65]. Compared to C-terminal EF-hand structures, N-terminal EF-hand structures (EF1 and EF2) with lower Ca^2+^-binding affinities played important roles in activating the kinases [35, 66]. These results suggested a high conservation of this family through evolution.

### Phylogenetics and synteny analysis of BdCDPKs

**Fig. 4:**
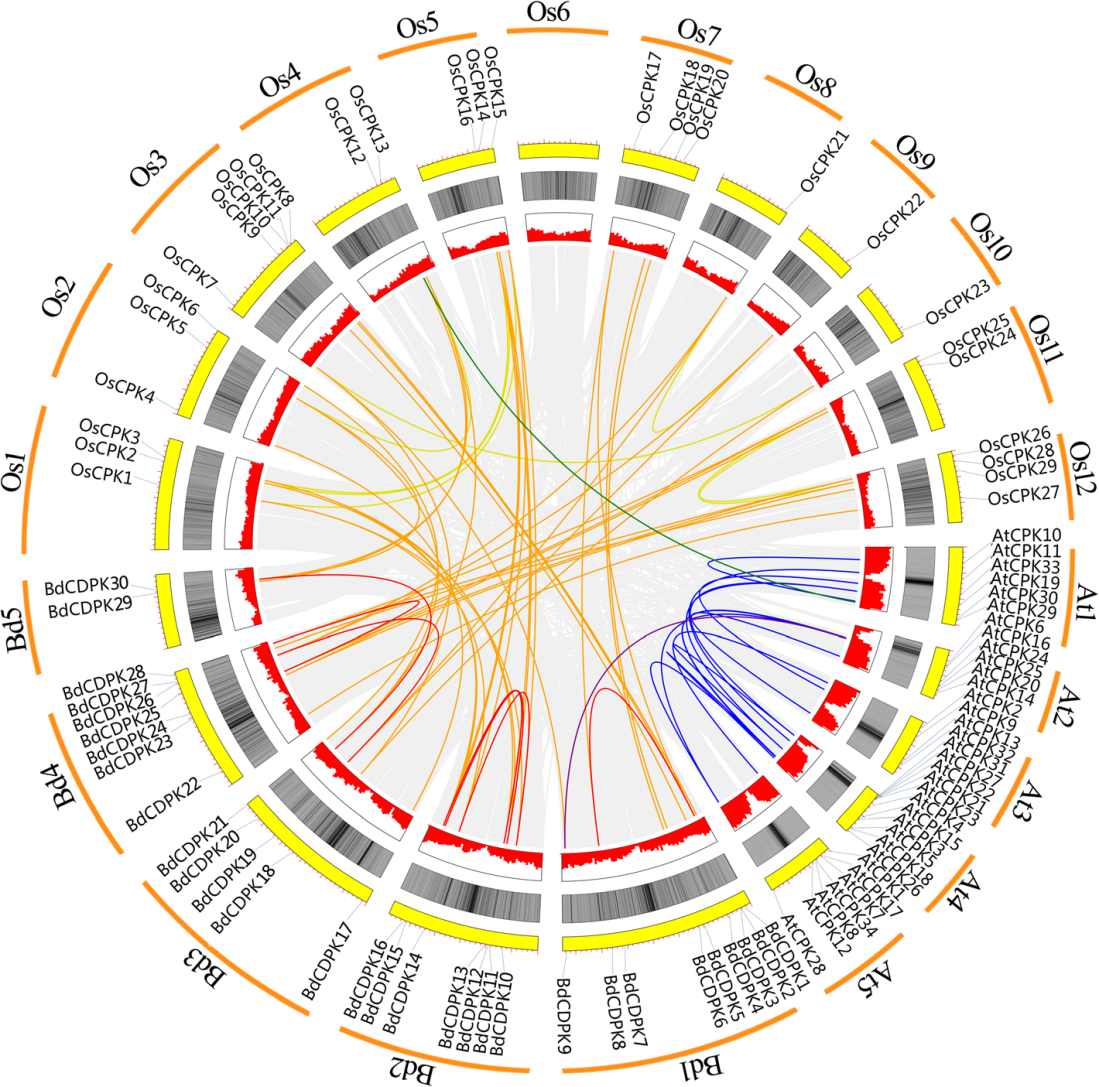
Synteny analysis of *CDPK* genes among Arabidopsis, rice and *B. distachyon*. Gray lines in the background indicate the collinear blocks within Arabidopsis, rice and *B. distachyon* genomes, while the colored lines highlight the syntenic *CDPK* gene pairs. Schematic representation was displayed by using the CIRCOS software. The size of chromosomes was consistent with the actual pseudo-chromosome size. Positions are in Mb

To explore the function and phylogenetic relationship of BdCDPK proteins between dicots and monocots, the dicot model plant Arabidopsis, the monocot model plant rice, and *B. distachyon* CDPK full-length amino acid sequences were used to construct an unrooted phylogenetic tree. The maximum likelihood phylogenetic distribution suggested that the organization of these BdCDPK proteins was very similar to each other in subgroup I, II, III, and IV, implying that CDPKs within these classes derived from a common ancestor (Additional file 11). In general, all CDPKs and their subgroups were present in monocots and dicots, indicating that the appearance of most of CDPKs in plants predated the monocot-dicot divergence and CDPK genes were conserved during evolution. Similar to the CDPKs in Arabidopsis and rice, subgroups I, II and III in *B. distachyon* consist of large numbers of CDPKs, which exhibit a great diversity in number among different plant species. For example, eleven CDPKs in subgroup I were found in *B. distachyon*, and eight CDPKs in subgroup II were found in *B. distachyon*. The members in subgroup IV contained the lowest number of CDPKs. However, nine AtCDPKs in the subgroups II was clustered into one sub-branch, which exhibited the difference between monocots and dicots, suggesting that these members might arise after dicot-monocot divergence. These results implied that the divergence in distribution of CDPK subgroups in different plant species might be because of the independent evolution of subgroup II among the different organisms. The phylogenetic similarity found in rice and *B. distachyon*, suggesting that they may have evolved conservatively, which was consistent with the synteny analysis results (Fig. 4). As a result, 72 collinear gene pairs among rice, Arabidopsis and *B. distachyon* CDPKs. Results indicated that large number of syntenic relationship events existed between rice and *B. distachyon*, indicating that many consensuses in CDPK protein may have existed before the species divergence between rice and *B. distachyon*. However, there are only one collinear gene pairs (AtCPK6 and BdCDPK09) existing between *B. distachyon* and Arabidopsis, suggesting that the origin of this gene pairs were very old.

### Expression profile of *BdCDPK* genes

**Fig. 5:**
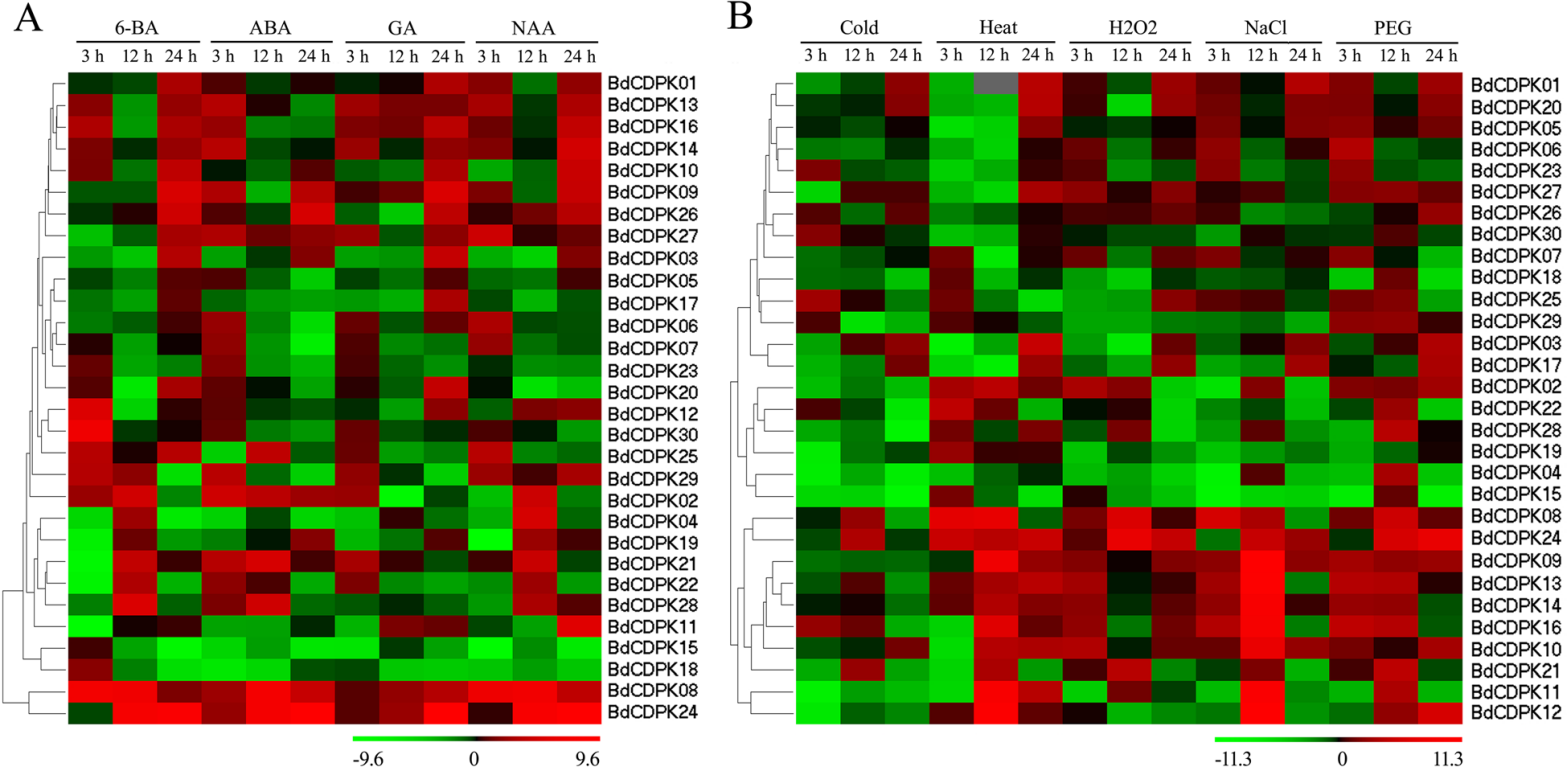
Expression patterns of *CDPK* genes in *B. distachyon* in response to phytohormone (**a**) and abiotic stresses (**b**). 6-BA (6-benzylaminopurine, 20 μM); ABA (abscisic acid, 100 μM); GA (gibberellin A3, 10 μM); NAA (1-naphthylacetic acid, 5 μM); Cold for 4 °C; Heat for 45 °C; H_2_O_2_ (10 mM); NaCl (200 mM) and PEG (polyethylene glycol, 20%), respectively. Levels of down expression (green) or up expression (red) are shown on a log2 scale from the high to the low expression of each *BdCDPK* gene

Extensive research has revealed the crosstalk between CDPK and phytohormones in plant defense and development processes [36, 37, 67]. Phytohormones played well-established roles in regulating plant signaling networks [68]. Incipiently, CDPKs were known to modulate Ca^2+^-dependent plant responses caused by phytohormones, mostly cytokinins, auxins and gibberellins. For example, the activity of CDPK was higher in GA-treated seeds than in untreated ones [69], the transcript level of *OsCDPK13* was increased by GA treatment in rice seedlings [70], and in Arabidopsis, CPK28 was identified as a key regulator of GA and JA levels [24, 71]. In tobacco leaves, a slight induction of *NtCDPK1* expression was observed by treated with IAA [72], and the expression level of *MsCPKs* was increased after auxin (2, 4-D) treated in alfalfa [73]. Further, an increasing body of evidence has shown that CDPKs regulate ABA-mediated signal transduction in plants [28, 29, 74]. In Arabidopsis, CPK4 and CPK11 were found to positively regulate calcium-mediated ABA signaling, while CPK21 and CPK23 were identified to act as negative regulators in ABA signaling pathway [28, 35, 75]. To better understand the role of BdCDPKs in phytohormone signaling pathway, the expression levels of the *BdCDPK* family genes in *B. distachyon* seedlings were detected by qRT-PCR under various phytohormones treatments, including 6-BA, ABA, GA and NAA, and then the green-red gradient heatmaps were generated by PermutMatrix program. Firstly, the expression levels of eight genes were determined as marker genes to evaluate the phytohormones treatments (Additional files 12 and 13). The expression levels of all marker genes exhibited obviously increased after phytohormones treatments, which was consistent with previous studies [76–78]. In this study, most members of *BdCDPK* genes exhibited a transcriptional expression changes in response to all tested phytohormones, and the transcriptional regulation of these *BdCDPK* genes was shown great divergence (Fig. 5). The real-time qPCR results revealed that most of *BdCDPK* genes (except *BdCDPK05*, *BdCDPK15* and *BdCDPK18*) were induced at least one phytohormone treatment, and nine *BdCDPKs* (*BdCDPK02*, *BdCDPK08*, *BdCDPK13*, *BdCDPK14*, *BdCDPK16*, *BdCDPK21*, *BdCDPK24*, *BdCDPK25* and *BdCDPK29*) were induced by these four types of phytohormones. Among these genes, the expression of seven *BdCDPKs* (*BdCDPK02*, *BdCDPK13*, *BdCDPK14*, *BdCDPK16*, *BdCDPK21*, *BdCDPK25* and *BdCDPK29*) were up-regulated within 12 h after phytohormone treatments, suggesting these *BdCDPK* genes were related to early response in phytohormone treatments, while the expression level of *BdCDPK24* was increased at 24 h post-treatment, implying this *BdCDPK* gene might be considered as a late response gene. As shown in Fig. 5, *BdCDPK08*was significantly up-regulated in the seedlings by treatment with all test phytohormones, suggesting BdCDPK08 might play critical roles in phytohormones-induced signaling pathway, and execute multifunction in plant growth and development, which was consistent with the previous reports that the homologs of *BdCDPK08* were played important roles in plant growth and development and response to abiotic and biotic stresses (Additional file 14) [32, 79–83]. It has been reported that CDPKs regulated ABA signal pathway by phosphorylating ABA-responsive element binding factors (ABFs) and ABA Insensitive (ABI). For example, AtCPK32 acted as an ABA signaling component that positively modulates the ABA-responsive gene expression via ABF4, while AtCPK12 was considered as a balancer in ABA signaling pathway by phosphorylating ABF1 and ABF4 as well as ABI2 [37, 74]. In response to exogenous ABA, transcripts of an overwhelming majority of *BdCDPK* genes were up-regulated within 3 h, suggesting that *BdCDPK* genes can quickly respond to ABA signal. It seems likely that expression level of *BdCDPK* genes from subgroup I and II were significantly increased in response to ABA which is in agreement with the changes in AtCPK3/4/6/11/23 in subgroup I and II of Arabidopsis [63, 68]. In addition, *BdCDPK23/27* kept at a high expression level after ABA treatment, and their homologous AtCPK4/11 was known to positively modulate calcium-mediated ABA signaling [28]. Although the role of CDPKs in Arabidopsis has been reported in many researches, the function of BdCDPKs in phytohormones signaling transduction remained to be studied in future.

It has been proven that many CDPKs play important roles in responses to cold, salt and drought, and the phytohormones were major modulators in plant adaptation and responses to various environmental stresses [2, 13, 84]. Transcriptome data showed that large numbers of *BdCDPK* genes were up-regulated by kinds of abiotic stresses, including cold, heat, drought and submergence (Additional file 4). To further evaluate the possible functional divergence of *BdCDPK* genes during abiotic stress and understand the relationship between phytohormone homeostasis and abiotic stresses, we determined the expression pattern of *BdCDPK* genes in response to cold, heat, H_2_O_2_, NaCl and PEG treatments. Firstly, the expression levels of marker genes were increased after abiotic treatments, as reported in previous studies (Additional files 12 and 13) [77, 85–87]. Generally, the expression pattern of *BdCDPK* genes in response to cold and heat stress was obviously different. For instance, 22 *BdCDPK* genes were up-regulate after heat treatment, while most of these *BdCDPK* genes were significantly down-regulate in response to cold stresses. Under cold stress, most of *BdCDPK* genes were down-regulated at the early phase of cold treatment, while four members (*BdCDPK16, 23, 25* and *30*) were significantly induced, implying most of BdCDPKs might act as early negative regulator in response to cold stress. The expression level of *BdCDPK01, 03, 10* and *17* were up-regulated 24 h post-cold treatment, suggesting these *BdCDPKs* were involved in the late response to cold stress. Under heat condition, most of *BdCDPK* genes were up-regulated 12–24 h post treatment, while four *BdCDPK* genes (*BdCDPK02*, *13*, *14* and *24*) were persistently up-regulated. It is possible that most of *BdCDPK* genes were transcriptionally activated only at a certain time point, until the enzymes exert their functions [41, 88]. But there were still a few *BdCDPK* genes which might execute their function continuously. Interestingly, it exhibited a similar expression pattern in the *B. distachyon* seedlings subjected to salt, drought and oxidative stresses, which was consisted with the previous reports that many CDPKs were involved in both salt and drought stresses, and they positively mediated tolerance to these stresses by protecting plants from reactive oxygen species (ROS) damage, with the uncontrolled generation of ROS being a common feature of these stresses [80, 89–91]. Notably, a set of genes (*BdCDPK09*, *10*, *11*, *12*, *13*, and *20*) were up-regulated 0–12 h post treatment with NaCl and PEG, while they were up-regulated 12–24 h post treatment with heat stress, implying these BdCDPKs might play different roles in response to these stresses. The present study, together with the previously public transcriptome data, revealed that osmotic stress induced expression of a different pattern of genes compared with temperature stress, which was consist with the expression pattern of VaCPKs (Fig. 5 and Additional file 4) [88]. Interestingly, some gene pairs in the terminal nodes of the phylogenetic tree showed similar expression pattern under different treatments. For instance, *BdCDPK02/08* were up-regulated under heat, H_2_O_2_ and drought stresses, while *BdCDPK10/14*, *BdCDPK13/16* and *BdCDPK23/27* were induced under H_2_O_2_, salt and drought stresses. These observations indicate that these CDPK genes might be function redundancy, or can cooperatively regulate a specific stimulation, which was consistent with the findings of many plants, such as *CDPK* gene in tomato, poplar and cucumber [62, 92, 93]. Nonetheless, some gene pairs exhibited a divergent transcriptional level. For example, *BdCDPK08* was up-regulated by salt stress, but the transcriptional level *BdCDPK02* was decreased under same condition. Transcriptional abundance of *BdCDPK22* was up-regulated by heat stress, while *BdCDPK22* was strongly down-regulated after heat treatment. These results imply complex functionality of BdCDPKs in multiple signaling pathways, and the evolutional variation in *cis*- or *trans*-acting element of such genes. Synteny analysis results indicated 72 collinear blocks of CDPK genes among rice, Arabidopsis and *B. distachyon* CDPKs (Fig. 4). Intriguingly, large number of correlated functional connections in syntenic gene pairs have been found. For example, AtCPK4 was proved as a positive regulator in involved in tolerance to salt, drought and oxidative stresses, and its collinear gene *BdCDPK23* was also up-regulated during salt, drought and oxidative stresses [28, 94]. Both *BdCDPK09* and its collinear gene *OsCDPK7* were involved in the response to salt and drought stresses [30]. Thus, the synteny analysis among Arabidopsis, rice and *B. distachyon* could provide insights into the prediction of gene function for *CDPK* genes. These results implied a conservation of gene function during the evolution of *CDPK* genes.

### Prediction of co-regulatory and interaction networks of *BdCDPKs, BdWRKYs and BdMAPK* cascade genes

**Fig. 6:**
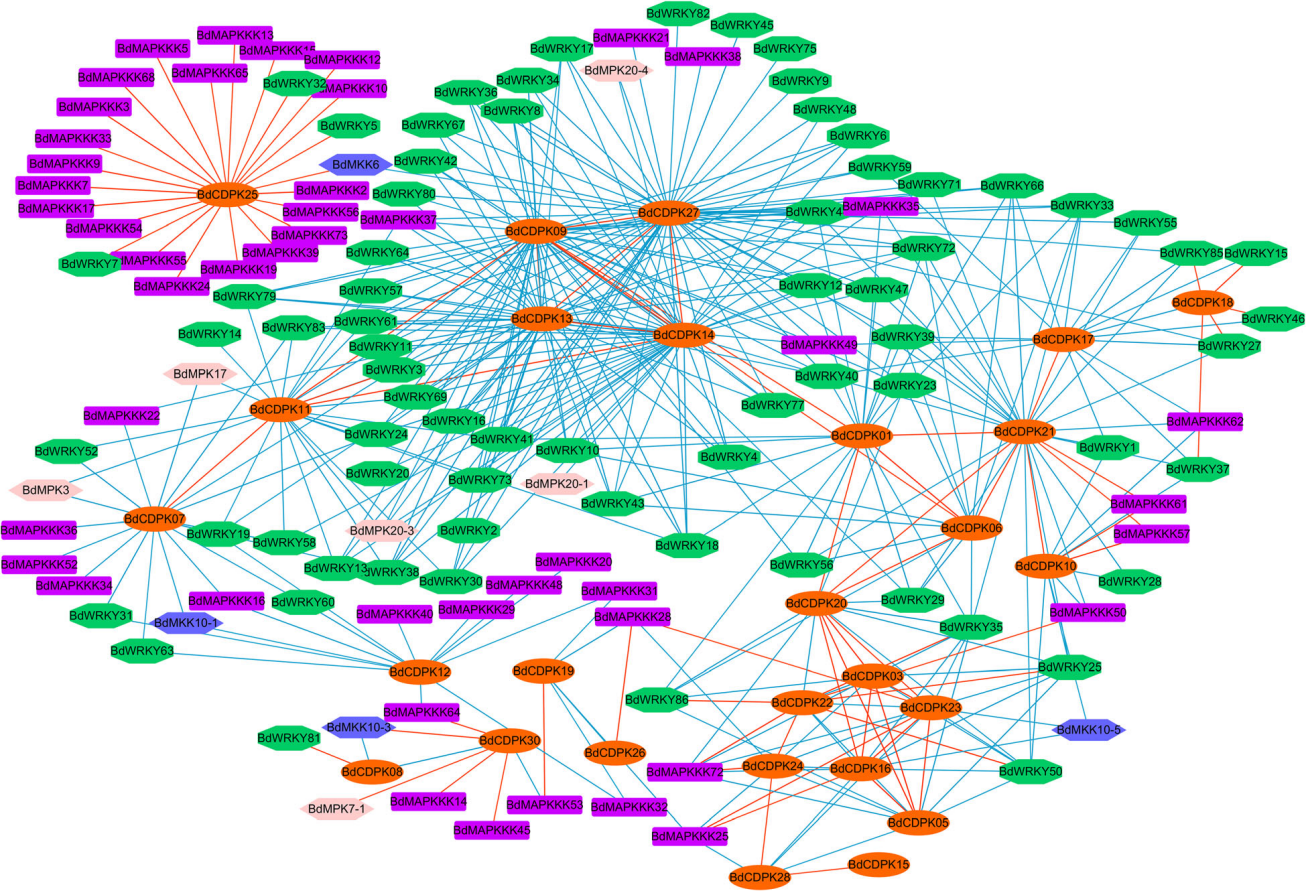
Regulatory networks of *CDPK* genes. Co-expression regulatory network among *BdCDPK*, *BdWRKY* and *BdMAPK* cascade genes upon different stress treatments based on the Pearson correlation coefficients of the relative expression of genes. The different colors represented the different family genes, orange for *BdCDPKs*, green for *BdWRKYs*, pink for *BdMPKs*, blue for *BdMKKs*, and purple for *BdMAPKKKs*

Ca^2+^ was considered as a critical pathway trigger in response to environmental stimuli and developmental cues, and it was a ubiquitous secondary messenger in cellular signal transduction, which was decoded by various Ca^2+^ binding proteins and kinases such as CDPK [3]. Besides the Ca^2+^ signaling, MAPK cascade was a pivotal phosphorylation pathway to transmit external or internal signals to downstream effectors. WRKY transcription factors were one of the largest families of transcriptional regulators, which could form integral parts of signalling networks to modulate many plant processes. Here, to explore the potential regulatory networks between *BdCDPKs* and *BdWRKYs* as well as that between *BdCDPKs* and *BdMAPK* cascade gene, we constructed the co-expression regulatory network among these genes upon different stress treatments by calculating the correlation coefficient of expression level between each two genes. Results showed that large number of *BdCDPK* genes exhibited co-expression correlation with *BdWRKYs* and *BdMAPK* cascade gene, suggesting these genes might be involved in same regulatory pathway (Fig. 6 and Additional file 15). Reports proved that the CDPK and MAPK pathways were often triggered by same environmental stimuli and developmental cues, it has been speculated that a potential crosstalk might exist between these two important protein kinase families [67, 95]. For instance, CDPKs and MAPK cascades differentially regulated flg22-induced early genes through CDPK/MAPK parallel or CDPK/MAPK synergistic regulation, implying an independent or co-regulation of transcription machinery in response to flg22 [12]. As shown in Fig. 6, *BdCDPK25* showed positive co-expression levels with a large number of BdMAPKKKs, indicating that *BdCDPK25* might be a key regulators in MAPK pathways, which was consist with the result that OsCPK18 was identified as an upstream kinase of MAPK (MPK5) in rice [96]. However, the prediction protein-protein interaction (PPI) result showed that *BdCDPK25* didn’t directly interact with MAPKKKs (Additional file 16). Moreover, CDPK signaling has been reported to compromise stress-induced MAPK activation in tobacco [67]. In this study, we also found that *BdCDPK07* and *BdCDPK12* exhibited negative expression correlation with some MAPK cascade genes, implying these CDPKs might mediated the inhibition of MAPK activation. The prediction PPI result also showed that a set of BdCDPKs (BdCDPK06, 07, 09, 17, 18 and 23) were predicted to be interacted with BdMAPKs, implying that BdCDPKs might be involved in MAPK pathways through directly interacting with MAPKs, but not the upstream components of MAPK pathways, such as MAPKKs and MAPKKKs (Additional file 16). The regulatory network between *BdCDPKs* and *BdWRKYs* was more complicated. As shown in Fig. 6, *BdCDPK09/13/14/27* showed a strong negative co-expression correlation with a set of *BdWRKYs*, indicating that these BdCDPKs might be a repressors to negatively regulate the stress response in *B. distachyon*. BdWRKYs were also predicted to be interacted with several members of BdCDPKs, suggesting BdCDPKs might phosphorylate WRKY transcription factor to execute their function in plant tolerance to environmental stresses. In Arabidopsis, Gao et al. found that sustained activation of CPK4, 5, 6, and 11 (subgroup I CDPKs) phosphorylate WRKY transcription factors important for activation of transcriptional reprogramming of immune genes [94]. Consistently, BdCDPK18 and BdCDPK22, members of subgroup I CDPKs, showed a strong co-expression correlation with a set of *BdWRKY* transcription factors, implying these BdCDPKs might be involved in WRKY transcription factors mediated stress response signaling network. The diagrammatic co-expression regulatory and predicted PPI network could reveal a deductive signaling pathway of stress response in *B. distachyon*, which showed that the BdCDPKs might act as both the activator and the repressor of WRKYs or MAPK cascade genes to modulate stress response processes. However, the accurate regulatory mechanisms among BdCDPKs, BdWRKYs and BdMAPK cascade of herbaceous plants during development and stress responses required further investigation.

## Conclusions

**Fig. 7:**
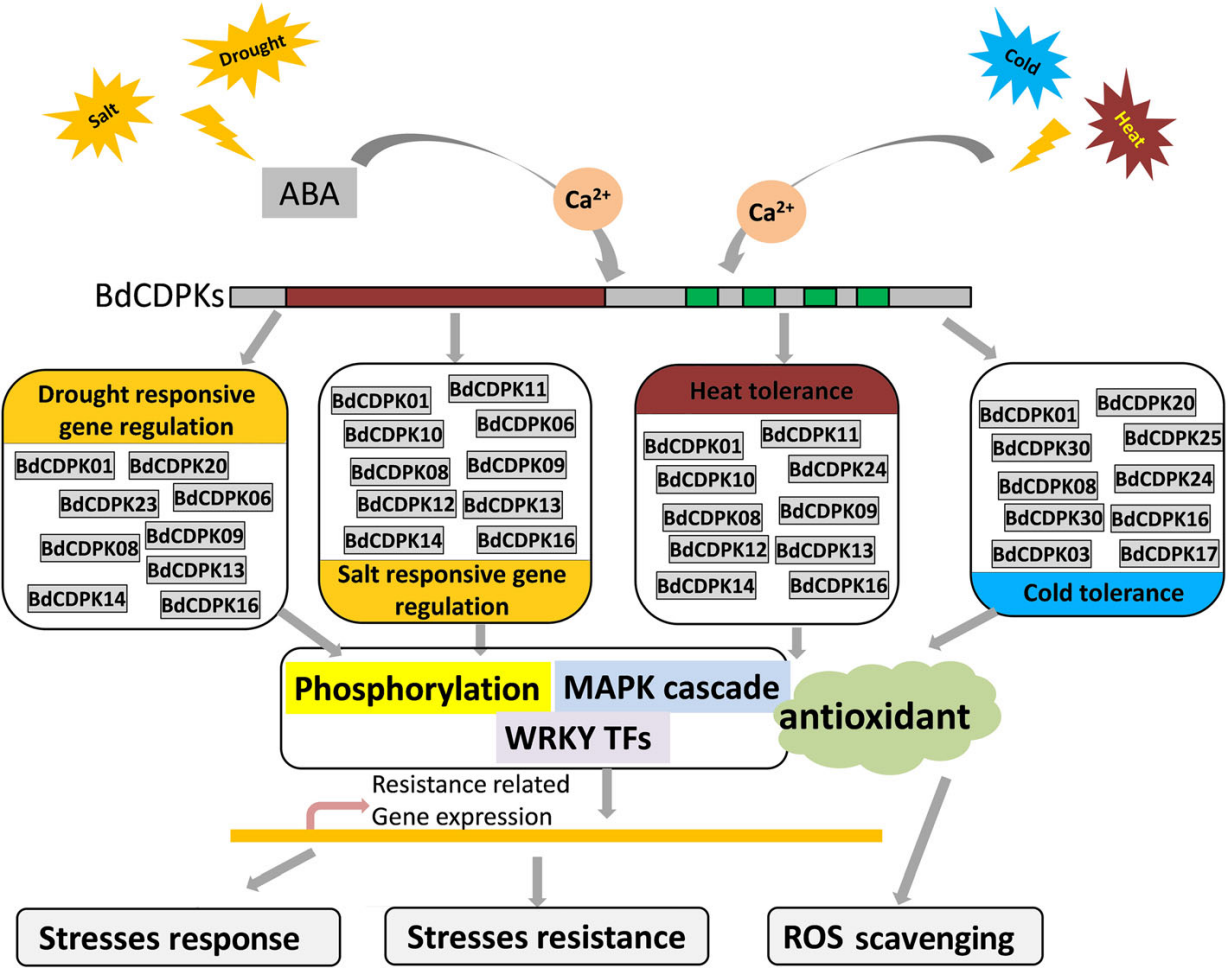
A summarizing model of BdCDPK function

The identification and systematical study of *CDPK* genes in *B. distachyon* would help scientist to better explore the functions of CDPKs in integrating Ca^2+^ signalling pathways in *B. distachyon* in adaptation to vagaries of environments. In this study, 30 members of *BdCDPK* genes were identified. The gene characterizations and phylogenies have been systematically analyzed. Phylogenetic tree revealed that BdCDPK family members can be clustered into four subgroup (I-IV), based upon sequence homology. And all BdCDPKs were possessed of a typical CDPK structure like AtCPKs, including a variable N-terminal domain, a catalytic Ser/Thr protein kinase domain, an autoinhibitory domain, and a EF-hand domain. However, we found that the structure of EF-hands (EF2 and EF4) were shown strong evolutionary divergence compared with CDPKs in Arabidopsis. Synteny results indicated that large number of syntenic relationship events existed between rice and *B. distachyon*, indicating that many consensuses in CDPK protein may have existed before the species divergence between rice and *B. distachyon*. The expression patterns revealed the involvement of *BdCDPK* genes in various phytohormones and response to abiotic stresses. Moreover, the co-expression and predicted PPI network implied that there was a complex regulatory network between BdCDPKs and BdWRKYs as well as that between BdCDPKs and BdMAPK cascade, and BdCDPKs might be both the activator and the repressor involved in WRKY transcription factors or MAPK cascade mediated stress response processes. Our study provided systematical study of *CDPK* genes in *B. distachyon* under multiple phytohormones and stresses conditions which is an important step for further investigation the functions of CDPKs across different plant species (Fig. 7).

## Methods

### Identification and characterization analysis of *B. distachyon CDPK* genes

All the *B. distachyon* genome sequence data were downloaded from Phytozome V12 (https://phytozome.jgi.doe.gov) [97]. The protein sequences of *A. thaliana* CDPK were obtained from TAIR database (http://www.arabidopsis.org). To identify the CDPK family members in *B. distachyon*, the Arabidopsis and rice CDPK sequences were used as the query to perform a BLASTP search against 52,972 sequences of the protein database of *B. distachyon*, with a cutoff *e*-value ≤e^− 10^. The SMART (http://smart.embl-heidelberg.de/) and InterPro (http://www.ebi.ac.uk/interpro/) online tools were used to analyze these potential sequences to validate the BLAST search [98]. The theoretical isoelectric point and molecular weight were estimated by pI/Mw tool (http://web.expasy.org/compute_pi), while WoLF PSORT predictor was used to predict the subcellular localization of BdCDPK proteins (https://wolfpsort.hgc.jp). All conserved domains were investigated by multiple alignment analyses using ClustalW, and the phylogenetic analysis for BdCDPKs was performed by using MEGA-X program by the maximum likelihood method, with bootstrap value from 1000 replicates indicated at each node with the following parameters: p-distance and pairwise deletion.

### Gene structure and chromosomal locations

The *BdCDPK* gene structures were displayed by comparing the coding sequences and corresponding genomic DNA sequences with the Gene Structure Display Server tools (http://gsds.cbi.pku.edu.cn/) [99]. The chromosomal locations of the *BdCDPK* genes were determined using the *B. distachyon* genome browser, and mapped by using a TBtools toolkit [100]. The Multiple Collinearity Scan toolkit (MCScanX) was used for the synteny analysis, and the result is graphic by Circos software (http://circos.ca/) [101, 102].

### Protein structure and conserved motif analysis

The MEME program (http://meme-suite.org/) was used to identify the conserved motifs of the *B. distachyon* CDPKs with the following parameters: any number of repetitions of a single motif, the maximum numbers of different motifs up to 15 motifs, the minimum motif width with six amino acids, the maximum motif width of a motif with 80 amino acids [103]. The details of sequence logo of motifs were shown in Additional file 1. The Interpro (www.ebi.ac.uk/interpro) and SMART(smart.embl-heidelberg.de) database were used to identify the conserved domains and important sites in *B. distachyon* CDPKs [104, 105]. Subsequently, the TBtools toolkit was used to draw the diagram.

### Expression analysis of BdCDPKs

Seeds of *B. distachyon* Bd21 were germinated on 1/2 Murashige and Skoog medium (MS) solid medium. Seedlings were transferred to soil in temperature-controlled (25 °C) growth chambers under a 16-h light/8-h dark cycle. The Bd21 seedlings were used for tissue-specific expression analysis and stress or hormone treatments according to previous work with some modifications [106]. For tissue-specific expression analysis, 2-week-old seedlings were used to collect the roots, stems and leaves, while 3-month-old seedlings were used to collect the flowers and seeds. For hormone and abiotic stress treatment, 2-week-old seedlings were treated in Murashige and Skoog medium (MS) liquid medium containing 100 μM abscisic acid (ABA), 20 μM 6-benzylaminopurine (6-BA), 5 μM 1-naphthylacetic acid (NAA), 10 μM gibberellin A3 (GA3), 20% polyethylene glycol (PEG), 200 mM NaCl and 10 mM H_2_O_2_ for 3 h, 12 h and 24 h, respectively. Cold and heat treatments were achieved by placing 2-week-old seedlings in MS liquid medium at 4 °C or 45 °C for 3 h, 12 h and 24 h, respectively. Samples were stored at − 80 °C after liquid nitrogen freezing if not immediately used for RNA isolation and subsequent analysis. Total RNA was extracted by the TRIzol method and treated with DNaseI to eliminate any DNA contamination. First-strand cDNA (20 μL) was synthesized according to the instructions for the PrimeScript™ RT Master Mix (Takara Biomedical Technology (Beijing) Co., Ltd., Beijing, China). Gene specific primers for quantitative real-time PCR are listed in Additional file 2. The expression of *CDPK* genes was assessed upon the qPCR result analysis. Each experiment was repeated three biological replications. *BdActin* (*Bradi2g24070*) gene was the internal reference gene. For tissue-specific analysis, the average of total Δ*C*Tvalue (Δ*C*T. average) was subtracted from all other Δ*C*T values to obtain second normal standardization, according to the previous method, using the formula: u = (Δ*C*T–Δ*C*T. average)/σ (in which, u is the value after normal standardization, and σ is the standard deviation) [107]. The *BdCDPK* gene expression profiles were calculated from the –ΔΔ*C*T value [−ΔΔ*C*T = (*C*Tcontrol.gene – *C*Tcontrol.actin) – (*C*Ttreat.gene – *C*Ttreat.actin)], and a heatmap was generated by PermutMatrixEN version 1.9.3 software. Two tailed Student’s t-test (P_0.05_) was used to determine the significant difference of relative expression of individual *BdCDPK* genes between control and different treatments (Microsoft Excel 2007). Fold-change greater than 2 with *p-value* of < 0.05 was defined as up-regulated gene, while a fold change of 0.5 or less was used to define down-regulated genes when the *p-value* of < 0.05 (Additional file 3). Raw publicly available transcriptome data for *B. distachyon* were downloaded from NCBI GEO and used to analyze the expression profiles of *BdCDPK* genes under diverse stress and hormone conditions (Additional files 4 and 5).

### Predicted co-expression and interaction network

The Pearson correlation coefficients (PCCs) of transcript levels of gene pairs were calculated by Microsoft Excel 2007, based on log2-transformed quantitative Real-Time (qRT)-PCR data. For gene co-regulatory network analysis, the gene pairs, whose PCCs was greater than 0.8, were selected. Based on the PCCs of these gene pairs, the co-expression networks were represented by using Cytoscape [108]. The predicted protein-protein interaction (PPI) network among BdCDPKs, BdWRKYs and BdMAPK cascade genes was generated by STRING V10.0 software online. The parameters were set as follows: meaning of network edges, confidence; active interaction sources, experiments; minimum required interaction score, 0.8.

## Supplementary information

**Additional file 1 **Sequence logos for the conserved motifs of CDPK proteins in *B. distachyon*.

**Additional file 2 **The list of qRT-PCR primers of *BdCDPK* genes.

**Additional file 3 **Expression data of *BdCDPK* genes after phytohormone treatment and abiotic stresses.

**Additional file 4 **Expression heatmap of *BdCDPK* genes under diverse stress and hormone conditions obtained from publicly available transcriptome data.

**Additional file 5.** List of publicly available transcriptome data accession number.

**Additional file 6 **The CDS and protein sequence of *BdCDPKs*.

**Additional file 7 **Phylogenetic relationships among the *BdCDPK* genes. Gene classes were indicated with different colors.

**Additional file 8 **Conserved domain and important site analysis of CDPKs in *B. distachyon*.

**Additional file 9.** Multiple alignments of conserved PDK domain in BdCDPK proteins.

**Additional file 10.** Multiple alignments of conserved EF domain in BdCDPK proteins.

**Additional file 11 **Phylogenetic analysis of Arabidopsis, rice and *B. distachyon* CDPKs.

**Additional file 12.** List of marker genes and qRT-PCR primers of marker genes.

**Additional file 13 **Expression of marker genes in *B. distachyon* in response to phytohormone and abiotic stresses.

**Additional file 14 **Homologous gene of *BdCDPKs* in responsive to hormone and abiotic stress.

**Additional file 15 **Expression patterns of *CDPK*, *WRKY* and *MAPK* cascade genes in *B. distachyon* in response to abiotic stresses.

**Additional file 16 **Predicted protein-protein interaction network of CDPKs, WRKYs and MAPK cascade members identified in *B. distachyon*.

## Data Availability

All data generated or analyzed during this study were included in this published article and the Additional files. The sequence data were obtained from Phytozome V12 (https://phytozome.jgi.doe.gov) and TAIR database (http://www.arabidopsis.org).
