# Supplementary material for: Genome-wide survey and expression analysis of calcium-dependent protein kinase (CDPK) in grass Brachypodium distachyon
Source: BMC Genomics. 2020 Jan 16;21:53. doi: 10.1186/s12864-020-6475-6 (PMC6966850; doi:10.1186/s12864-020-6475-6)
Supplement: Supplementary file 2 — Additional file 2 The list of qRT-PCR primers of BdCDPK genes. [file 12864_2020_6475_MOESM2_ESM.doc]

Additional file 2 The list of qRT-PCR primers of *BdCDPK* genes.

| **Gene Name** | **Forward Primer** | **Reverse Primer** |
| --- | --- | --- |
| **BdCDPK01** | CAGGGAGTAGCACAGGCAAT | GCAGTCTTGCTCGGACAGTA |
| **BdCDPK02** | AACCAGAACGAGGATTCGCC | CCAGAATGGAGGAACCCCAC |
| **BdCDPK03** | TGGTGGTCTGGCTCCTGATA | TAGGTGATTTGCCCGCTGTT |
| **BdCDPK04** | AACGCTTGCTTGTTCTGCTG | TAGGTCTGGGCTTCGGGTTA |
| **BdCDPK05** | GCGATTAACAGCCCAGCAAG | TTTATGCCAGCCACCTCCTC |
| **BdCDPK06** | GTAAGGAACTTGGCCGTGGA | GCACACTGCTCTTGTCCTCA |
| **BdCDPK07** | GAGGGTGGTGAGTTGCTTGA | TCTGTAGCCTTGAGGGGTGA |
| **BdCDPK08** | ACGTGATCTCCATTGTCGGG | GTCTCTGTGCATGACCCCAA |
| **BdCDPK09** | GTGCGCCGTGAGATACAGAT | TGGCACATAGCCACAATGCT |
| **BdCDPK10** | CGGAGAGGCCCCAGATAAAC | ATTGTCCCGCTGTTGTCAGT |
| **BdCDPK11** | ATAAAGGAAGACGGCGAGGC | CCGCTGTTGTCAGAGTCCAT |
| **BdCDPK12** | GCATCCGTGGGTGATAGGAG | TCGGAGGTAAGGTCATGGGT |
| **BdCDPK13** | TCTTCAAACCCGGCGAGAAA | AGCTTGTGCCACTCCTTGTT |
| **BdCDPK14** | ACGGCACTATGGAGCAGAAG | TCTTTGGGGTCTTGTCGCAG |
| **BdCDPK15** | TCAAGCAAGGGGAGGTGTTC | CGCAGGATGGAGTTGAAGGT |
| **BdCDPK16** | CGGCTCCCATCTTGCTGAAT | GCCTCTCGAAGCTCATCTGG |
| **BdCDPK17** | ATGAACCGACCTGTTGCTGT | ACTGCTGCATCTTTCTCGCT |
| **BdCDPK18** | TGTGCGACGTGAAATCCAGA | AATGGCACTTCGCTATGGCA |
| **BdCDPK19** | ACAAGAACGGCAACCTCTCC | CCAGGTACTCGTCGTTGGTC |
| **BdCDPK20** | CGATTTCGGCCTCTCTGTGT | TGCTGTGTCTCTGCCCAAAA |
| **BdCDPK21** | GGCAACGGAGAAGACGAAGA | CGGAGAGCAGGATGTAGACG |
| **BdCDPK22** | GTGGGTAATGAGGAGGACGC | CCGCAGAGCAGGATGTAGAC |
| **BdCDPK23** | TGGCTTGAAAAGAGTGGGCT | AGCCACTTCCATCCTTGTCG |
| **BdCDPK24** | GGAGATCAAGGGGCTCAAGG | CGGTGACGAACTCCTCGTAG |
| **BdCDPK25** | TCCCTCCTTTCTGGGGAGAC | AGCGACACATTTGGAGCCTT |
| **BdCDPK26** | CTGGACTGCAAGGAGTTCGT | CTTCCCGTCCTTGTCGATGT |
| **BdCDPK27** | AAATCCATCCCCAAGCGGAA | CGCTCGCTGTAATGTCCCTT |
| **BdCDPK28** | CATTTTTCTCTCCGGCGTGC | ATTGAGGACTTGGATGGCGG |
| **BdCDPK29** | CATCGACTTCGGTCTCTCCG | TTACCTGCCCAGAAAGGTGG |
| **BdCDPK30** | AAGAAGATGGCTTTGCGGGT | CCACATCCGCTGCTTCCATA |
| **BdActin (XM_014899375)^a^** | GTGTCCTGAAGTGCTGTTCC | TCTCCTTGCTCATGCGATCA |
| **BdUbi (Bradi3g04730)**  **(XM_003574817)^a^** | TGACACCATCGACAACGTGA | GAGGGTGGACTCCTTCTGGA |

a. Genebank accession number
