# Supplementary material for: Genome-wide survey and expression analysis of calcium-dependent protein kinase (CDPK) in grass Brachypodium distachyon
Source: BMC Genomics. 2020 Jan 16;21:53. doi: 10.1186/s12864-020-6475-6 (PMC6966850; doi:10.1186/s12864-020-6475-6)
Supplement: Supplementary file 3 — Additional file 3 Expression data of BdCDPK genes after phytohormone treatment and abiotic stresses. [file 12864_2020_6475_MOESM3_ESM.doc]

Additional file 3A Expression data of *BdCDPK* genes after phytohormone treatment

|  |  | 6-BA | | | | | |  |  | ABA | | | | | |  |  | GA | | | | | |  |  | NAA | | | | | |  |
| --- | --- | --- | --- | --- | --- | --- | --- | --- | --- | --- | --- | --- | --- | --- | --- | --- | --- | --- | --- | --- | --- | --- | --- | --- | --- | --- | --- | --- | --- | --- | --- | --- |
|  | 3h | | | 12h | | 24h | | | 3h | | | 12h | | 24h | | | 3h | | | 12h | | 24h | | | 3h | | | 12h | | 24h | | |
| Gene name | FC^*^ | | P^*^ | FC^*^ | P^*^ | FC | P | | FC | | P | FC | P | FC | P | | FC | | P | FC | P | FC | P | | FC | | P | FC | P | FC | P | |
| *BdCDPK01* | -0.26 | | 0.49 | -0.53 | 0.45 | **2.85** | **0.00** | | 0.64 | | 0.10 | -0.37 | 0.48 | 0.13 | 0.60 | | -0.15 | | 0.54 | 0.06 | 0.46 | **3.04** | **0.01** | | **1.76** | | **0.00** | -1.28 | 0.08 | **2.01** | **0.03** | |
| *BdCDPK02* | **2.23** | | **0.00** | **4.59** | **0.01** | -1.76 | 0.07 | | **4.47** | | **0.00** | **3.48** | **0.02** | **2.40** | **0.02** | | **2.26** | | **0.02** | -8.08 | 0.10 | -0.47 | 0.24 | | **-3.70** | | **0.00** | **4.27** | **0.02** | **-1.53** | **0.00** | |
| *BdCDPK03* | **-2.27** | | **0.01** | **-3.99** | **0.03** | **3.19** | **0.01** | | **-2.51** | | **0.00** | -0.30 | 0.55 | **1.65** | **0.00** | | **-2.51** | | **0.00** | **-2.11** | **0.00** | **3.99** | **0.00** | | **-2.99** | | **0.03** | -4.67 | 0.11 | 1.62 | 0.20 | |
| *BdCDPK04* | **-4.93** | | **0.00** | **2.39** | **0.01** | **-6.32** | **0.03** | | **-4.57** | | **0.00** | -0.54 | 0.23 | **-4.82** | **0.01** | | **-3.84** | | **0.00** | 0.27 | 0.44 | **-1.24** | **0.00** | | **-2.98** | | **0.01** | **4.80** | **0.00** | **-1.02** | **0.02** | |
| *BdCDPK05* | -0.46 | | 0.12 | -1.59 | 0.07 | 0.78 | 0.02 | | 0.63 | | 0.06 | -0.94 | 0.05 | **-4.27** | **0.03** | | -0.45 | | 0.09 | **-1.28** | **0.04** | 0.67 | 0.02 | | -1.12 | | 0.07 | **-1.39** | **0.00** | 0.48 | 0.37 | |
| *BdCDPK06* | **-1.44** | | **0.05** | -0.84 | 0.03 | 0.47 | 0.31 | | **2.20** | | **0.01** | **-1.68** | **0.02** | -5.29 | 0.07 | | **1.03** | | **0.05** | -0.68 | 0.07 | 0.96 | 0.15 | | **2.92** | | **0.00** | -0.55 | 0.17 | -0.66 | 0.15 | |
| *BdCDPK07* | 0.17 | | 0.54 | **-2.53** | **0.03** | 0.00 | 0.99 | | **1.98** | | **0.04** | **-2.14** | **0.03** | **-6.72** | **0.00** | | 0.64 | | 0.25 | **-1.78** | **0.01** | -1.25 | 0.25 | | **2.17** | | **0.00** | -1.24 | 0.06 | -0.61 | 0.11 | |
| *BdCDPK08* | **7.21** | | **0.00** | **6.99** | **0.00** | **1.51** | **0.02** | | **2.40** | | **0.00** | **7.47** | **0.00** | **4.00** | **0.00** | | 0.77 | | 0.05 | **2.08** | **0.01** | **3.22** | **0.00** | | **6.82** | | **0.00** | **7.17** | **0.00** | 3.71 | 0.00 | |
| *BdCDPK09* | -0.72 | | 0.13 | -0.73 | 0.36 | **5.13** | **0.00** | | **3.01** | | **0.00** | **-3.04** | **0.04** | **3.36** | **0.05** | | 0.47 | | 0.45 | 1.10 | 0.19 | **5.23** | **0.00** | | 1.52 | | 0.07 | **-1.05** | **0.03** | 4.02 | 0.07 | |
| *BdCDPK10* | **1.45** | | **0.02** | **-1.32** | **0.01** | **3.71** | **0.00** | | -0.07 | | 0.92 | -0.93 | 0.01 | 0.75 | 0.02 | | -0.81 | | 0.01 | **-1.30** | **0.00** | **2.94** | **0.00** | | **-2.82** | | **0.03** | -1.00 | 0.14 | 4.13 | 0.13 | |
| *BdCDPK11* | **-8.54** | | **0.00** | 0.07 | 0.95 | 0.34 | 0.53 | | -2.61 | | 0.09 | **-2.55** | **0.01** | -0.17 | 0.88 | | **-3.21** | | **0.01** | **1.47** | **0.00** | 1.05 | 0.28 | | -0.51 | | 0.29 | **-2.53** | **0.03** | 5.54 | 0.06 | |
| *BdCDPK12* | **5.31** | | **0.01** | **-4.63** | **0.01** | 0.25 | 0.52 | | 0.85 | | 0.34 | -0.31 | 0.82 | -0.65 | 0.37 | | -0.20 | | 0.72 | -2.42 | 0.16 | **1.95** | **0.03** | | -0.92 | | 0.19 | **1.48** | **0.03** | 1.86 | 0.32 | |
| *BdCDPK13* | **1.81** | | **0.02** | -2.17 | 0.08 | 2.11 | 0.10 | | **3.28** | | **0.00** | 0.13 | 0.66 | -1.81 | 0.27 | | **2.43** | | **0.01** | **1.41** | **0.00** | 1.43 | 0.07 | | **2.75** | | **0.01** | -0.31 | 0.39 | **2.93** | **0.03** | |
| *BdCDPK14* | **1.49** | | **0.00** | -0.20 | 0.39 | 2.22 | 0.27 | | **3.38** | | **0.00** | -0.53 | 0.48 | -0.04 | 0.97 | | **2.30** | | **0.00** | -0.17 | 0.46 | **2.02** | **0.00** | | **1.59** | | **0.00** | -0.06 | 0.91 | 4.71 | 0.19 | |
| *BdCDPK15* | 0.44 | | 0.45 | **-2.67** | **0.00** | **-7.76** | **0.00** | | -4.85 | | 0.06 | **-2.43** | **0.00** | **-6.03** | **0.00** | | **-5.89** | | **0.00** | -0.36 | 0.57 | **-2.56** | **0.00** | | **-8.51** | | **0.00** | **-1.91** | **0.00** | **-6.37** | **0.00** | |
| *BdCDPK16* | **3.03** | | **0.01** | -2.27 | 0.08 | **2.90** | **0.00** | | **2.21** | | **0.01** | **-1.57** | **0.03** | -1.35 | 0.08 | | **1.58** | | **0.02** | **1.31** | **0.03** | **3.51** | **0.00** | | **1.15** | | **0.03** | -0.23 | 0.55 | 3.79 | 0.10 | |
| *BdCDPK17* | **-1.32** | | **0.00** | **-2.50** | **0.00** | 0.89 | 0.27 | | -1.02 | | 0.27 | **-2.17** | **0.00** | **-2.50** | **0.00** | | **-2.32** | | **0.00** | **-3.01** | **0.00** | **2.84** | **0.00** | | -0.55 | | 0.11 | **-3.23** | **0.02** | -0.68 | 0.71 | |
| *BdCDPK18* | 1.79 | | 0.06 | **-1.61** | **0.01** | **-5.33** | **0.00** | | **-6.04** | | **0.00** | **-4.94** | **0.00** | -0.65 | 0.02 | | -0.55 | | 0.52 | **-4.10** | **0.00** | **-4.22** | **0.00** | | **-3.98** | | **0.00** | **-2.43** | **0.00** | **-4.07** | **0.00** | |
| *BdCDPK19* | **-6.88** | | **0.00** | 1.11 | 0.26 | **-2.31** | **0.00** | | **-1.58** | | **0.03** | -0.06 | 0.94 | **1.71** | **0.01** | | -3.38 | | 0.06 | **-1.30** | **0.02** | 0.70 | 0.53 | | **-8.87** | | **0.00** | **2.30** | **0.02** | 0.46 | 0.55 | |
| *BdCDPK20* | 0.72 | | 0.26 | **-6.12** | **0.00** | **2.73** | **0.05** | | 0.90 | | 0.05 | -0.01 | 0.97 | -2.60 | 0.07 | | 0.19 | | 0.69 | -0.86 | 0.10 | **3.84** | **0.02** | | -0.01 | | 0.98 | **-5.66** | **0.00** | **-3.73** | **0.03** | |
| *BdCDPK21* | **-8.09** | | **0.01** | **3.77** | **0.01** | 0.37 | 0.24 | | **3.30** | | **0.00** | **4.91** | **0.00** | 0.48 | 0.62 | | **2.71** | | **0.00** | 0.33 | 0.39 | -0.58 | 0.67 | | 0.39 | | 0.00 | **4.16** | **0.00** | -0.49 | 0.15 | |
| *BdCDPK22* | **-7.35** | | **0.00** | 3.01 | 0.17 | **-3.14** | **0.00** | | **1.96** | | **0.02** | 0.59 | 0.28 | **-2.84** | **0.00** | | **1.53** | | **0.04** | **-1.84** | **0.04** | **-2.59** | **0.01** | | **-1.91** | | **0.03** | 2.41 | 0.06 | **-2.29** | **0.00** | |
| *BdCDPK23* | **1.00** | | **0.02** | **-2.68** | **0.00** | **-1.55** | **0.01** | | **1.63** | | **0.00** | **-2.04** | **0.03** | **-3.25** | **0.01** | | 0.56 | | 0.06 | **-1.04** | **0.01** | **-1.98** | **0.04** | | -0.20 | | 0.43 | **-1.96** | **0.00** | **-1.78** | **0.00** | |
| *BdCDPK24* | -0.51 | | 0.45 | **7.80** | **0.00** | **8.50** | **0.00** | | **2.12** | | **0.02** | **7.50** | **0.00** | **9.51** | **0.00** | | 0.77 | | 0.00 | **2.24** | **0.01** | **9.62** | **0.00** | | 0.28 | | 0.21 | **7.94** | **0.00** | **9.50** | **0.00** | |
| *BdCDPK25* | **3.29** | | **0.00** | 0.10 | 0.81 | **3.30** | **0.00** | | **-4.42** | | **0.00** | **3.67** | **0.00** | -0.79 | 0.13 | | **1.09** | | **0.05** | -2.38 | 0.11 | **-1.44** | **0.04** | | **3.35** | | **0.00** | **-1.69** | **0.00** | -1.11 | 0.06 | |
| *BdCDPK26* | -0.26 | | 0.51 | 0.16 | 0.91 | **4.46** | **0.01** | | 0.67 | | 0.18 | -0.39 | 0.51 | **4.92** | **0.01** | | -0.88 | | 0.26 | **-4.12** | **0.01** | 3.60 | 0.08 | | 0.28 | | 0.63 | **1.27** | **0.05** | **3.30** | **0.04** | |
| *BdCDPK27* | -3.74 | | 0.30 | -0.85 | 0.43 | 2.71 | 0.06 | | **3.04** | | **0.00** | 1.10 | 0.32 | 1.92 | 0.10 | | **2.34** | | **0.01** | -0.74 | 0.54 | 1.93 | 0.10 | | **4.36** | | **0.00** | 0.29 | 0.77 | 1.05 | 0.26 | |
| *BdCDPK28* | **-1.57** | | **0.01** | **5.21** | **0.00** | -0.92 | 0.08 | | **1.44** | | **0.03** | **4.52** | **0.00** | -1.11 | 0.56 | | -0.76 | | 0.14 | -0.18 | 0.75 | -0.88 | 0.36 | | -2.26 | | 0.09 | **2.86** | **0.02** | 0.73 | 0.12 | |
| *BdCDPK29* | **3.22** | | **0.01** | 1.93 | 0.07 | **-5.46** | **0.00** | | **3.40** | | **0.00** | **-1.09** | **0.04** | **-4.45** | **0.00** | | **2.02** | | **0.00** | -0.25 | 0.82 | **-4.38** | **0.00** | | **2.28** | | **0.00** | 0.46 | 0.31 | **2.69** | **0.00** | |
| *BdCDPK30* | **7.04** | | **0.00** | -0.31 | 0.25 | 0.05 | 0.53 | | **1.01** | | **0.00** | -1.45 | 0.06 | **-2.22** | **0.01** | | **1.04** | | **0.00** | -0.67 | 0.01 | -0.21 | 0.28 | | 0.58 | | 0.02 | -0.06 | 0.77 | -2.29 | 0.13 | |

*FC: Fold-change (log2); P:p-value

Red font indicate significant up-regulation, while blue indicate significant down-regulation.

Additional file 3B Expression data of *BdCDPK* genes after abiotic stresses

|  |  | Cold | | | | | |  | | Heat | | | | | |  | | H2O2 | | | | | |  | | NaCl | | | | | |  | | PEG | | | | | |  | |
| --- | --- | --- | --- | --- | --- | --- | --- | --- | --- | --- | --- | --- | --- | --- | --- | --- | --- | --- | --- | --- | --- | --- | --- | --- | --- | --- | --- | --- | --- | --- | --- | --- | --- | --- | --- | --- | --- | --- | --- | --- | --- |
|  | 3h | | | 12h | | 24h | | | 3h | | | 12h | | 24h | | | 3h | | | 12h | | 24h | | | 3h | | | 12h | | 24h | | | 3h | | | 12h | | 24h | | |  |
| Gene name | FC^*^ | | P^*^ | FC | P | FC | P | | FC | | P | FC | P | FC | P | | FC | | P | FC | P | FC | P | | FC | | P | FC | P | FC | P | | FC | | P | FC | P | FC | P | |  |
| *BdCDPK01* | **-2.49** | | **0.01** | -0.56 | 0.00 | **2.30** | **0.00** | | **-3.61** | | **0.00** | NA | NA | **3.94** | **0.00** | | 0.57 | | 0.02 | -0.76 | 0.01 | **2.76** | **0.00** | | **1.20** | | **0.00** | -0.03 | 0.86 | **3.72** | **0.00** | | **1.88** | | **0.00** | -0.61 | 0.06 | **2.83** | **0.01** | |  |
| *BdCDPK02* | **-4.26** | | **0.02** | -1.69 | 0.14 | -4.22 | 0.07 | | **3.20** | | **0.00** | **4.11** | **0.03** | 1.39 | 0.08 | | **3.30** | | **0.03** | 2.04 | 0.09 | **-4.99** | **0.00** | | **-6.82** | | **0.00** | 1.93 | 0.13 | **-4.51** | **0.00** | | **1.90** | | **0.00** | 1.67 | 0.17 | **3.01** | **0.01** | |  |
| *BdCDPK03* | **-3.12** | | **0.00** | 0.73 | 0.04 | **2.25** | **0.00** | | **-8.43** | | **0.00** | **-3.14** | **0.01** | **4.79** | **0.00** | | **-2.78** | | **0.02** | -7.29 | 0.18 | 1.23 | 0.12 | | **-1.09** | | **0.02** | 0.10 | 0.87 | **2.00** | **0.00** | | -0.76 | | 0.05 | 0.49 | 0.10 | **3.54** | **0.00** | |  |
| *BdCDPK04* | **-7.77** | | **0.00** | -3.32 | 0.27 | **-7.73** | **0.00** | | **-4.35** | | **0.00** | -1.12 | 0.17 | -0.22 | 0.36 | | **-4.12** | | **0.00** | **-2.95** | **0.00** | **-5.28** | **0.03** | | **-8.54** | | **0.00** | 0.83 | 0.18 | **-3.92** | **0.00** | | **-4.41** | | **0.00** | **3.24** | **0.00** | **-4.69** | **0.01** | |  |
| *BdCDPK05* | -0.17 | | 0.45 | -0.69 | 0.00 | 0.02 | 0.91 | | **-6.55** | | **0.04** | **-5.33** | **0.01** | **2.13** | **0.00** | | -0.15 | | 0.69 | -0.41 | 0.02 | 0.03 | 0.93 | | **1.77** | | **0.00** | -0.03 | 0.72 | **2.00** | **0.00** | | **2.20** | | **0.00** | 0.28 | 0.01 | **1.51** | **0.01** | |  |
| *BdCDPK06* | **-1.66** | | **0.02** | **-1.95** | **0.01** | -0.25 | 0.02 | | **-3.32** | | **0.00** | **-5.43** | **0.05** | 0.20 | 0.17 | | **1.29** | | **0.03** | **-1.52** | **0.00** | 0.40 | 0.00 | | **2.33** | | **0.01** | **-1.11** | **0.02** | 0.29 | 0.10 | | **3.74** | | **0.00** | **-1.08** | **0.00** | -0.43 | 0.29 | |  |
| *BdCDPK07* | **-1.41** | | **0.00** | -0.72 | 0.11 | -0.02 | 0.93 | | **1.54** | | **0.00** | **-7.16** | **0.00** | 0.18 | 0.58 | | **1.28** | | **0.01** | -1.69 | 0.16 | **1.09** | **0.03** | | **1.87** | | **0.00** | -0.32 | 0.42 | 0.24 | 0.43 | | **2.06** | | **0.00** | -0.08 | 0.82 | -3.97 | 0.32 | |  |
| *BdCDPK08* | -0.14 | | 0.38 | **2.56** | **0.00** | **-3.10** | **0.00** | | **6.96** | | **0.00** | **7.25** | **0.00** | **-1.10** | **0.03** | | **1.64** | | **0.00** | **6.13** | **0.00** | 0.50 | 0.16 | | **5.69** | | **0.00** | **3.43** | **0.01** | **-2.61** | **0.00** | | **1.46** | | **0.00** | **5.14** | **0.00** | **1.11** | **0.02** | |  |
| *BdCDPK09* | **-1.49** | | **0.02** | **-1.15** | **0.02** | **-1.29** | **0.02** | | -0.34 | | 0.58 | **8.12** | **0.01** | 2.50 | 0.11 | | **1.92** | | **0.01** | 0.00 | 1.00 | **1.89** | **0.00** | | **2.25** | | **0.00** | **10.95** | **0.00** | **2.24** | **0.00** | | **3.12** | | **0.00** | **2.37** | **0.00** | **2.69** | **0.00** | |  |
| *BdCDPK10* | -0.73 | | 0.02 | -0.20 | 0.10 | **1.56** | **0.00** | | **-6.21** | | **0.00** | **3.82** | **0.00** | **3.46** | **0.00** | | **3.67** | | **0.00** | -0.09 | 0.57 | **1.22** | **0.00** | | **1.21** | | **0.00** | **7.60** | **0.00** | **2.52** | **0.00** | | **1.59** | | **0.00** | 0.23 | 0.16 | **3.13** | **0.00** | |  |
| *BdCDPK11* | **-8.19** | | **0.00** | -2.86 | 0.22 | **-4.14** | **0.00** | | **-5.81** | | **0.01** | **9.60** | **0.00** | **3.93** | **0.00** | | **-5.21** | | **0.04** | **1.49** | **0.10** | -0.45 | 0.14 | | **-4.13** | | **0.01** | **10.06** | **0.00** | **-2.18** | **0.01** | | **-3.79** | | **0.00** | **3.48** | **0.00** | -3.71 | 0.07 | |  |
| *BdCDPK12* | **-7.25** | | **0.00** | -1.11 | 0.07 | **-2.09** | **0.00** | | 0.81 | | 0.23 | **10.31** | **0.00** | **1.06** | **0.02** | | 0.07 | | 0.93 | **-3.70** | **0.02** | **-2.17** | **0.00** | | -1.69 | | 0.16 | **11.37** | **0.00** | **-2.46** | **0.00** | | -1.39 | | 0.12 | **2.41** | **0.01** | **5.86** | **0.00** | |  |
| *BdCDPK13* | -0.82 | | 0.02 | 0.81 | 0.01 | **-2.33** | **0.03** | | 1.24 | | 0.19 | 3.16 | 0.07 | **4.02** | **0.01** | | **3.04** | | **0.00** | -0.08 | 0.92 | 0.41 | 0.45 | | **3.03** | | **0.00** | **10.65** | **0.00** | -1.72 | 0.06 | | **3.80** | | **0.00** | **3.64** | **0.00** | 0.19 | 0.80 | |  |
| *BdCDPK14* | -0.11 | | 0.56 | 0.06 | 0.83 | **-1.43** | **0.00** | | **1.05** | | **0.03** | **3.70** | **0.00** | **1.99** | **0.00** | | **2.26** | | **0.00** | -0.23 | 0.70 | 0.94 | 0.00 | | **2.42** | | **0.00** | **9.52** | **0.00** | 0.44 | 0.76 | | **2.60** | | **0.00** | **2.48** | **0.00** | -0.76 | 0.13 | |  |
| *BdCDPK15* | **-5.82** | | **0.00** | **-5.41** | **0.00** | **-9.51** | **0.04** | | 1.61 | | 0.06 | **-1.30** | **0.00** | -6.33 | 0.09 | | 0.22 | | 0.68 | **-2.77** | **0.00** | **-4.51** | **0.00** | | **-8.85** | | **0.00** | **-5.66** | **0.00** | **-5.28** | **0.01** | | **-7.63** | | **0.00** | **1.12** | **0.00** | **-8.13** | **0.00** | |  |
| *BdCDPK16* | **2.50** | | **0.01** | **1.29** | **0.00** | -3.46 | 0.20 | | **-5.44** | | **0.00** | **6.67** | **0.00** | 1.16 | 0.12 | | **2.38** | | **0.01** | **-1.59** | **0.02** | **1.38** | **0.05** | | **3.71** | | **0.00** | **8.94** | **0.00** | **-1.81** | **0.00** | | **4.35** | | **0.00** | **3.83** | **0.00** | -0.91 | 0.34 | |  |
| *BdCDPK17* | **-3.78** | | **0.00** | **-1.89** | **0.00** | **1.52** | **0.01** | | **-5.44** | | **0.00** | **-8.11** | **0.01** | **2.69** | **0.00** | | **-1.18** | | **0.00** | **-3.01** | **0.04** | **2.37** | **0.00** | | **-3.19** | | **0.04** | **-2.12** | **0.01** | **2.94** | **0.00** | | -0.13 | | 0.66 | **-1.00** | **0.01** | **3.41** | **0.00** | |  |
| *BdCDPK18* | -1.35 | | 0.11 | **-1.21** | **0.00** | **-4.50** | **0.04** | | 1.08 | | 0.15 | **-3.95** | **0.00** | -0.29 | 0.55 | | **-2.80** | | **0.02** | **-5.05** | **0.00** | -0.33 | 0.57 | | -0.96 | | 0.47 | -0.79 | 0.01 | -0.18 | 0.71 | | **-4.79** | | **0.00** | **1.30** | **0.02** | **-5.95** | **0.00** | |  |
| *BdCDPK19* | **-7.61** | | **0.00** | **-1.52** | **0.02** | -0.54 | 0.22 | | **2.56** | | **0.01** | 0.38 | 0.73 | 0.42 | 0.27 | | **-4.55** | | **0.05** | -0.62 | 0.70 | -2.46 | 0.23 | | **-4.64** | | **0.00** | **-2.59** | **0.11** | **-1.53** | **0.02** | | **-3.39** | | **0.02** | -1.24 | 0.23 | 0.08 | 0.92 | |  |
| *BdCDPK20* | -0.40 | | 0.34 | -0.17 | 0.54 | 2.02 | 0.12 | | **-3.28** | | **0.04** | **-3.98** | **0.00** | **3.88** | **0.03** | | 0.48 | | 0.50 | **-5.73** | **0.00** | 2.55 | 0.06 | | **1.51** | | **0.01** | -0.18 | 0.33 | 1.89 | 0.13 | | **1.86** | | **0.01** | -0.08 | 0.87 | 2.14 | 0.10 | |  |
| *BdCDPK21* | **-3.41** | | **0.00** | **2.60** | **0.00** | **-3.08** | **0.00** | | **-5.64** | | **0.00** | **3.42** | **0.00** | **-2.72** | **0.00** | | 0.57 | | 0.14 | **3.93** | **0.00** | **-2.01** | **0.01** | | -0.46 | | 0.24 | **1.82** | **0.01** | **-3.68** | **0.00** | | 0.54 | | 0.38 | **4.20** | **0.00** | -0.63 | 0.53 | |  |
| *BdCDPK22* | 0.66 | | 0.22 | -0.56 | 0.40 | **-7.11** | **0.00** | | **4.32** | | **0.01** | 1.29 | 0.25 | -3.68 | 0.08 | | -0.03 | | 0.97 | 0.23 | 0.76 | **-5.61** | **0.02** | | **-2.01** | | **0.02** | -0.61 | 0.22 | **-4.19** | **0.00** | | -0.58 | | 0.37 | **2.72** | **0.00** | **-4.78** | **0.00** | |  |
| *BdCDPK23* | **1.85** | | **0.00** | -0.69 | 0.01 | **-1.03** | **0.02** | | -5.30 | | 0.13 | **-3.51** | **0.01** | 0.39 | 0.14 | | 0.85 | | 0.02 | **-2.35** | **0.02** | -0.78 | 0.04 | | **2.18** | | **0.00** | **-1.71** | **0.01** | -0.64 | 0.05 | | **2.34** | | **0.00** | -0.74 | 0.01 | **-1.27** | **0.01** | |  |
| *BdCDPK24* | -0.66 | | 0.02 | **3.60** | **0.00** | -0.41 | 0.44 | | **4.91** | | **0.00** | **4.00** | **0.00** | **4.87** | **0.00** | | 0.91 | | 0.02 | **7.34** | **0.00** | **4.65** | **0.00** | | **-1.56** | | **0.00** | **4.77** | **0.00** | **2.63** | **0.00** | | -0.28 | | 0.12 | **5.79** | **0.00** | **7.34** | **0.00** | |  |
| *BdCDPK25* | **3.03** | | **0.00** | 0.18 | 0.49 | -1.72 | 0.23 | | **1.45** | | **0.01** | **-1.58** | **0.01** | **-6.05** | **0.00** | | -3.11 | | 0.37 | **-2.78** | **0.00** | **2.18** | **0.01** | | 0.96 | | 0.22 | 0.59 | 0.08 | -0.57 | 0.59 | | 1.54 | | 0.17 | **1.99** | **0.00** | **-2.99** | **0.01** | |  |
| *BdCDPK26* | 0.81 | | 0.14 | -1.29 | 0.32 | 0.97 | 0.28 | | -1.91 | | 0.54 | -1.03 | 0.45 | 0.13 | 0.94 | | 0.58 | | 0.43 | 0.55 | 0.21 | 1.22 | 0.15 | | 0.53 | | 0.30 | **-2.11** | **0.01** | -1.44 | 0.13 | | -0.58 | | 0.20 | 0.11 | 0.89 | **2.52** | **0.03** | |  |
| *BdCDPK27* | **-5.66** | | **0.00** | 0.64 | 0.58 | 0.66 | 0.68 | | **-3.81** | | **0.00** | **-5.71** | **0.02** | **3.21** | **0.02** | | **2.23** | | **0.01** | 0.19 | 0.84 | 2.01 | 0.09 | | 0.25 | | 0.63 | 0.66 | 0.52 | -0.57 | 0.58 | | **2.05** | | **0.01** | 2.22 | 0.12 | 1.16 | 0.24 | |  |
| *BdCDPK28* | **-3.32** | | **0.00** | -1.68 | 0.08 | **-8.98** | **0.01** | | **1.53** | | **0.03** | -0.58 | 0.60 | **1.73** | **0.01** | | -0.84 | | 0.01 | 1.61 | 0.15 | **-5.65** | **0.00** | | **-2.81** | | **0.00** | 0.94 | 0.14 | **-2.38** | **0.00** | | **-3.62** | | **0.01** | **3.99** | **0.00** | 0.03 | 0.94 | |  |
| *BdCDPK29* | 0.68 | | 0.38 | **-6.64** | **0.00** | **-3.66** | **0.02** | | 0.79 | | 0.07 | 0.06 | 0.93 | -0.97 | 0.01 | | -3.18 | | 0.55 | **-3.17** | **0.00** | **-1.80** | **0.00** | | -1.67 | | 0.16 | -1.15 | 0.26 | -3.23 | 0.14 | | **2.31** | | **0.05** | **2.38** | **0.00** | 0.36 | 0.45 | |  |
| *BdCDPK30* | **2.03** | | **0.00** | 0.17 | 0.37 | -0.34 | 0.03 | | **-4.43** | | **0.00** | -3.68 | 0.15 | 0.24 | 0.01 | | -0.13 | | 0.54 | -0.63 | 0.06 | -0.64 | 0.05 | | **-2.71** | | **0.00** | 0.16 | 0.02 | -0.36 | 0.00 | | -0.37 | | 0.48 | 0.73 | 0.01 | -0.60 | 0.00 | |  |

*FC: Fold-change (log2); P:p-value

Red font indicate significant up-regulation, while blue indicate significant down-regulation.

Additional file 3C The *C*t values of *BdActin* and *BdUbiquitin* gene under phytohormone treatment and abiotic stresses.

|  |  | Control | |  | |  | 6-BA | |  | |  | ABA | |  | |  | GA | |  | |  | NAA | |  |
| --- | --- | --- | --- | --- | --- | --- | --- | --- | --- | --- | --- | --- | --- | --- | --- | --- | --- | --- | --- | --- | --- | --- | --- | --- |
|  | 12h | | 24h | | 12h | | | 24h | | 12h | | | 24h | | 12h | | | 24h | | 12h | | | 24h | |
| *BdAct* | 18.46 | | 21.71 | | 25.36 | | | 25.00 | | 25.93 | | | 26.84 | | 21.06 | | | 26.33 | | 26.33 | | | 26.61 | |
| *BdUbi* | 14.30 | | 17.86 | | 21.42 | | | 21.11 | | 22.24 | | | 22.66 | | 17.32 | | | 22.26 | | 22.40 | | | 22.49 | |

|  |  | Cold | |  | |  | Heat | |  | |  | H_2_O_2_ | |  | |  | NaCl | |  | |  | PEG | |  |
| --- | --- | --- | --- | --- | --- | --- | --- | --- | --- | --- | --- | --- | --- | --- | --- | --- | --- | --- | --- | --- | --- | --- | --- | --- |
|  | 12h | | 24h | | 12h | | | 24h | | 12h | | | 24h | | 12h | | | 24h | | 12h | | | 24h | |
| *BdAct* | 22.43 | | 18.92 | | 26.98 | | | 22.45 | | 25.71 | | | 22.90 | | 22.80 | | | 19.56 | | 24.36 | | | 24.68 | |
| *BdUbi* | 18.64 | | 14.91 | | 22.74 | | | 18.16 | | 21.99 | | | 19.02 | | 19.07 | | | 15.25 | | 20.47 | | | 20.80 | |
