# Supplementary figures and images for: Genome-wide survey and expression analysis of calcium-dependent protein kinase (CDPK) in grass Brachypodium distachyon
Source: BMC Genomics. 2020 Jan 16;21:53. doi: 10.1186/s12864-020-6475-6 (PMC6966850; doi:10.1186/s12864-020-6475-6)

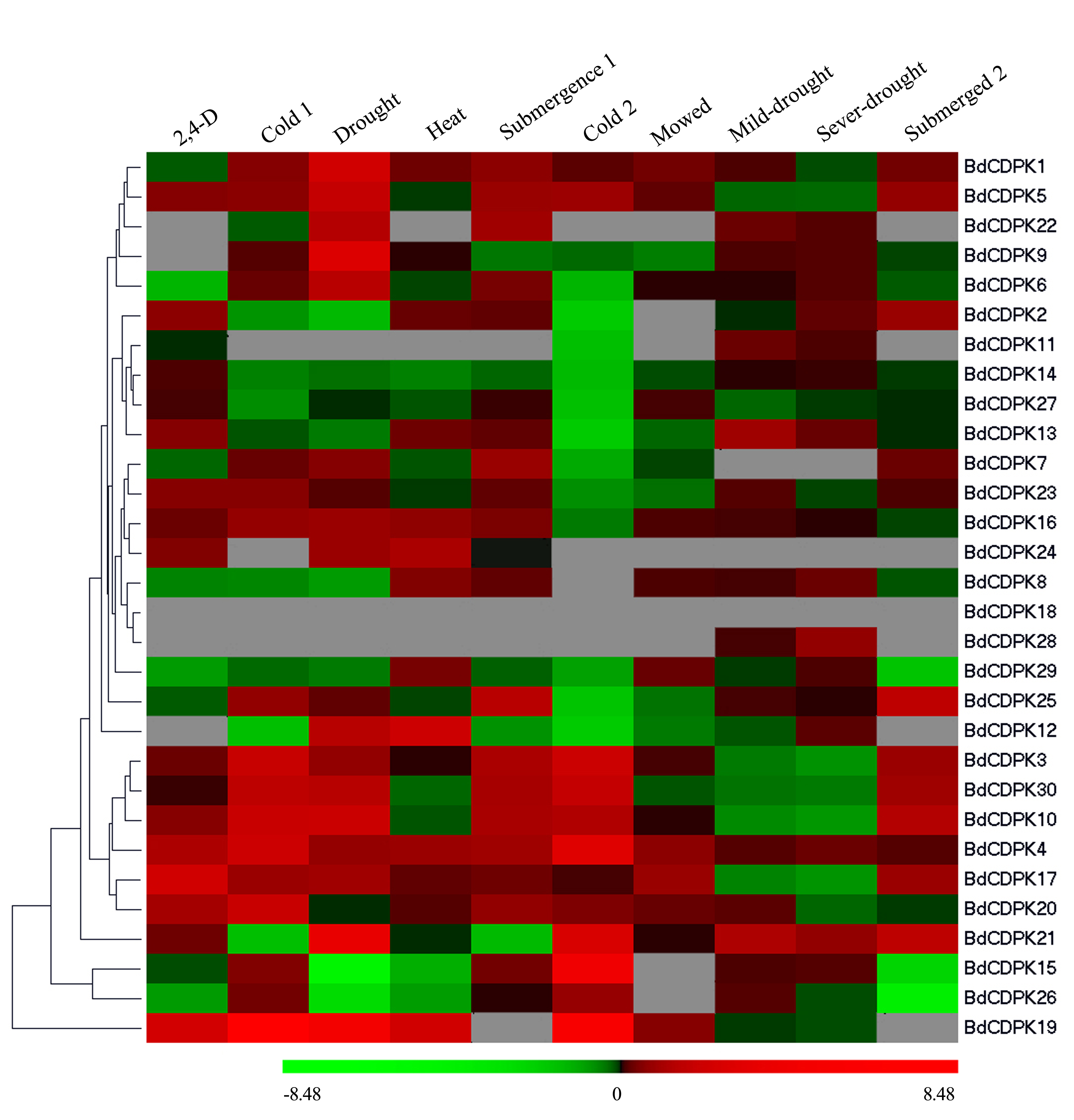

Supplement: Supplementary file 4 — Additional file 4 Expression heatmap of BdCDPK genes under diverse stress and hormone conditions obtained from publicly available transcriptome data. [file 12864_2020_6475_MOESM4_ESM.tif]

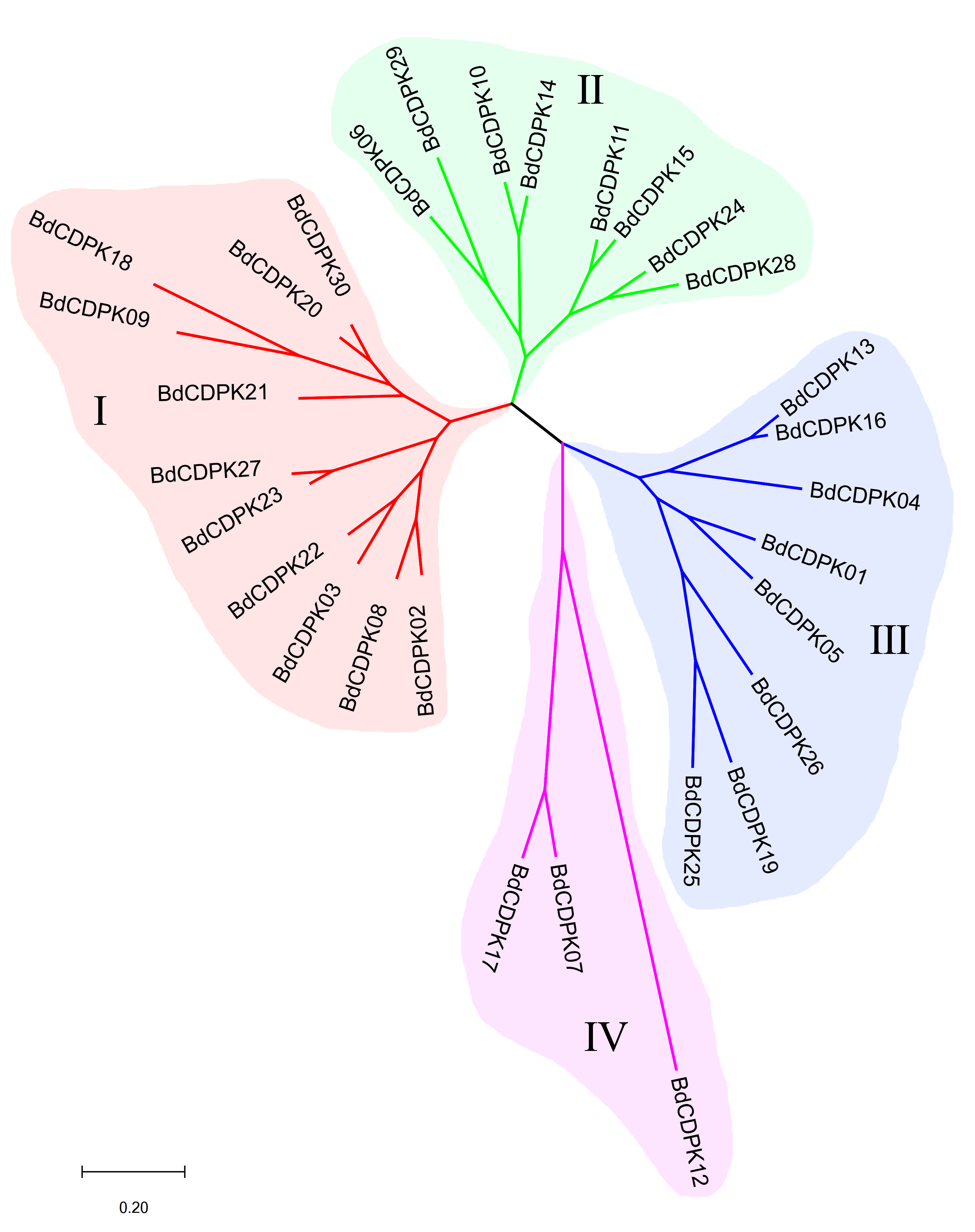

Supplement: Supplementary file 7 — Additional file 7 Phylogenetic relationships among the BdCDPK genes. Gene classes were indicated with different colors. [file 12864_2020_6475_MOESM7_ESM.tif]

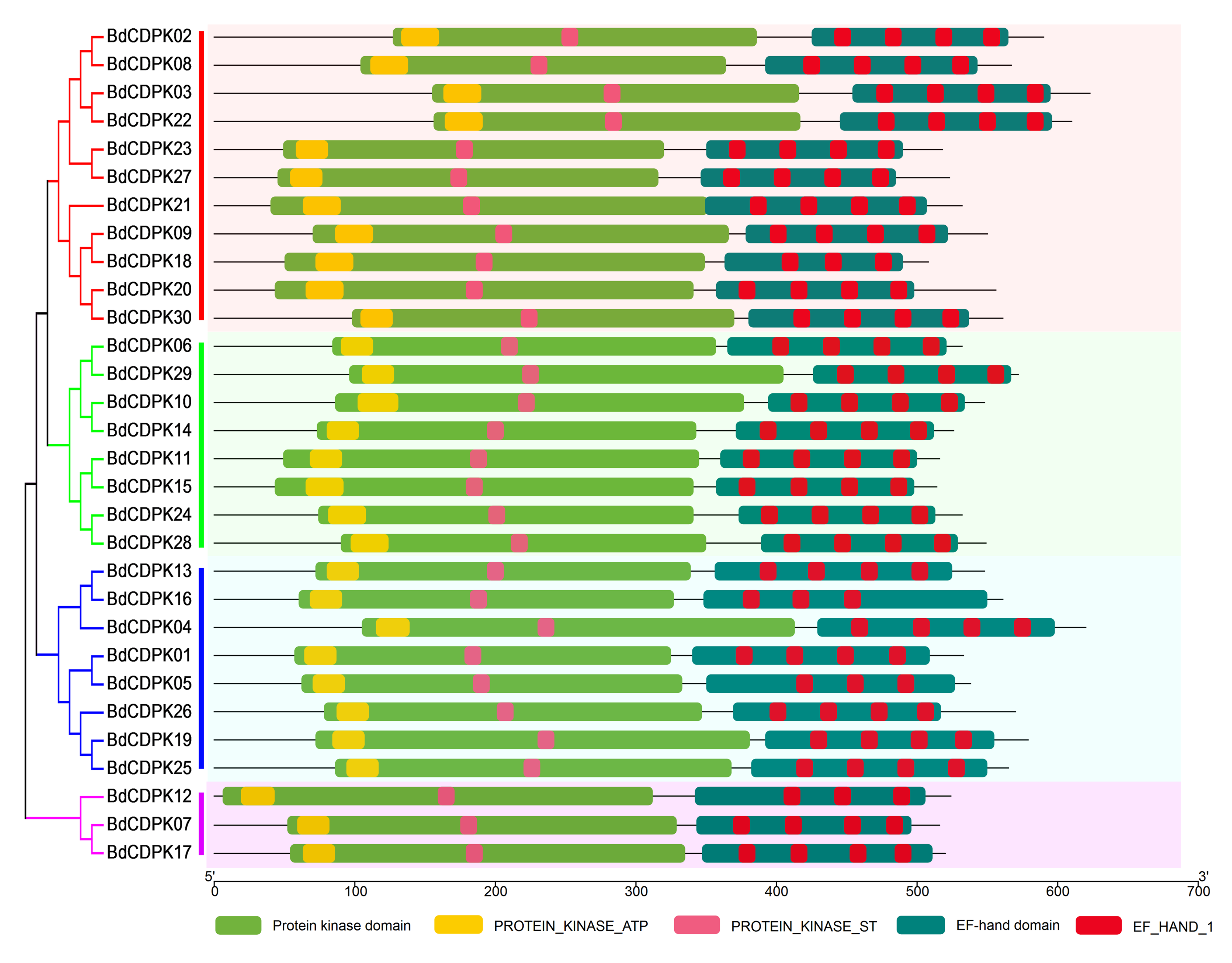

Supplement: Supplementary file 8 — Additional file 8 Conserved domain and important site analysis of CDPKs in B. distachyon. [file 12864_2020_6475_MOESM8_ESM.tif]

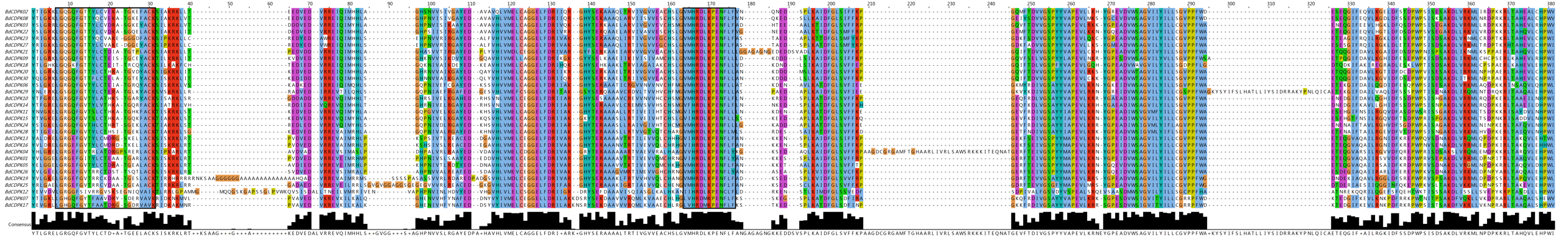

Supplement: Supplementary file 9 — Additional file 9. Multiple alignments of conserved PDK domain in BdCDPK proteins. [file 12864_2020_6475_MOESM9_ESM.tif]

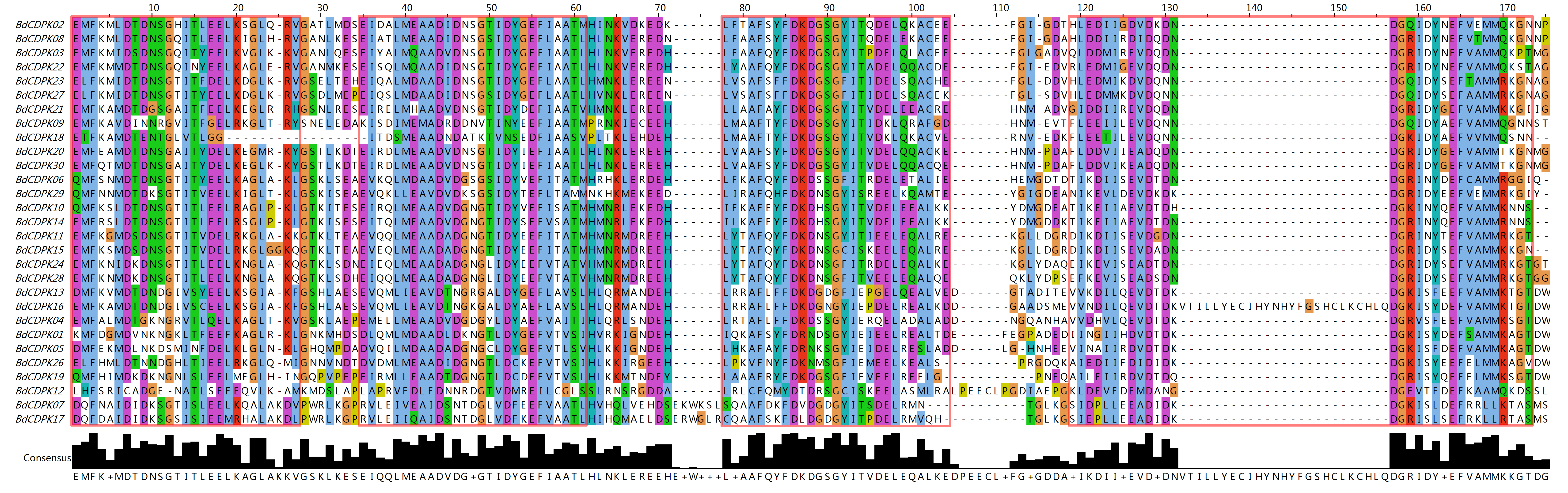

Supplement: Supplementary file 10 — Additional file 10. Multiple alignments of conserved EF domain in BdCDPK proteins. [file 12864_2020_6475_MOESM10_ESM.tif]

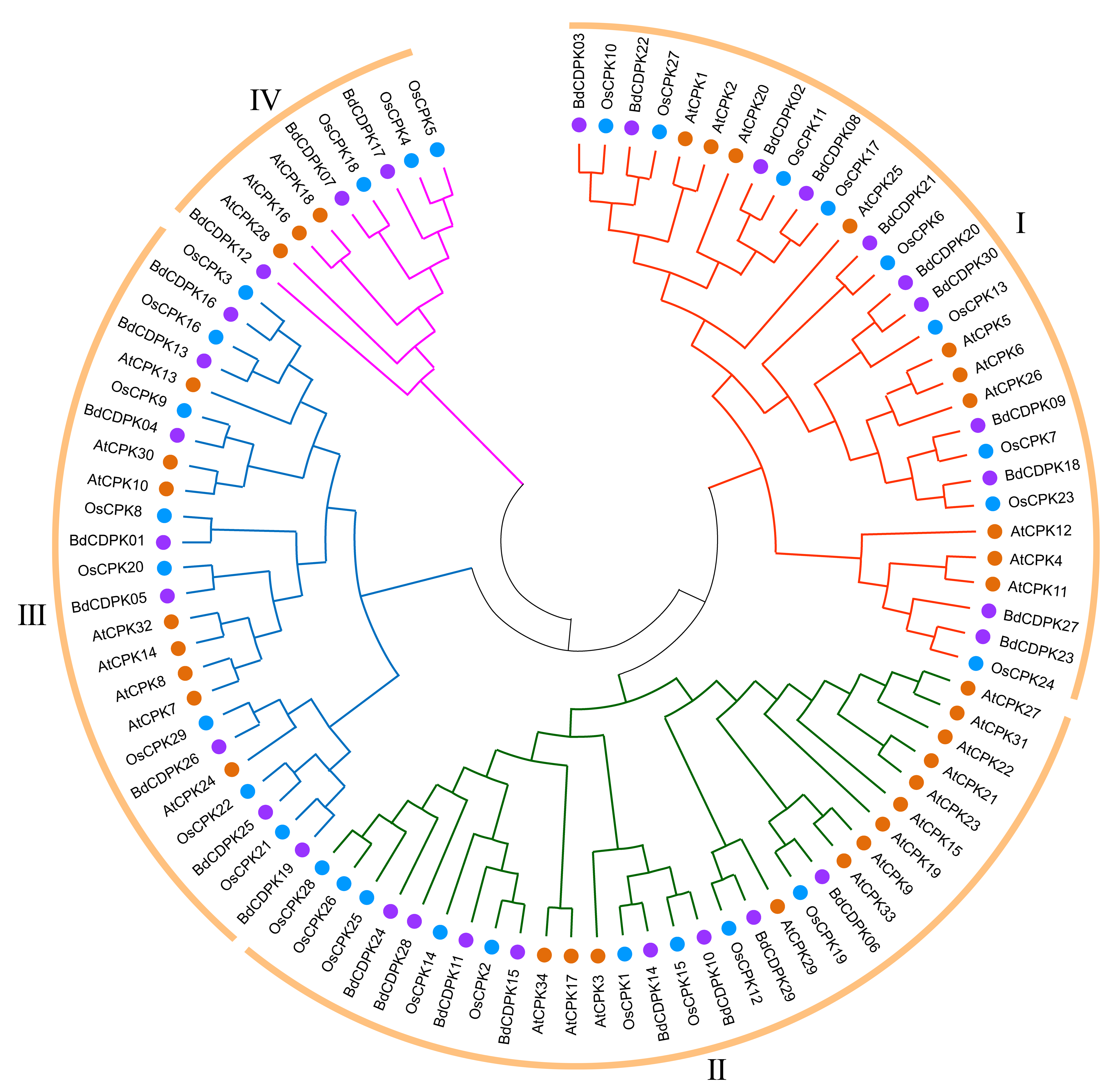

Supplement: Supplementary file 11 — Additional file 11 Phylogenetic analysis of Arabidopsis, rice and B. distachyon CDPKs. [file 12864_2020_6475_MOESM11_ESM.tif]

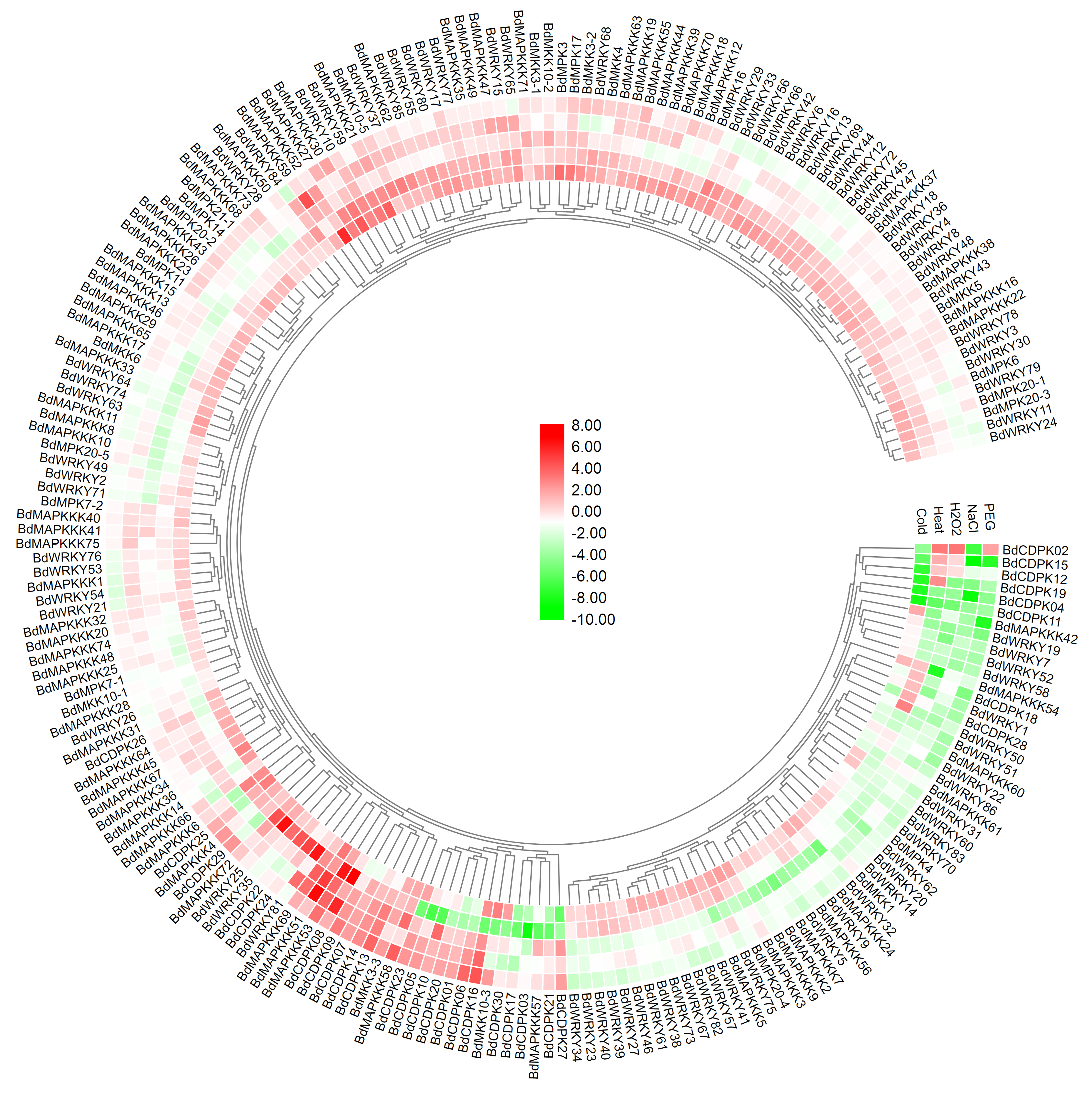

Supplement: Supplementary file 15 — Additional file 15 Expression patterns of CDPK, WRKY and MAPK cascade genes in B. distachyon in response to abiotic stresses. [file 12864_2020_6475_MOESM15_ESM.tif]

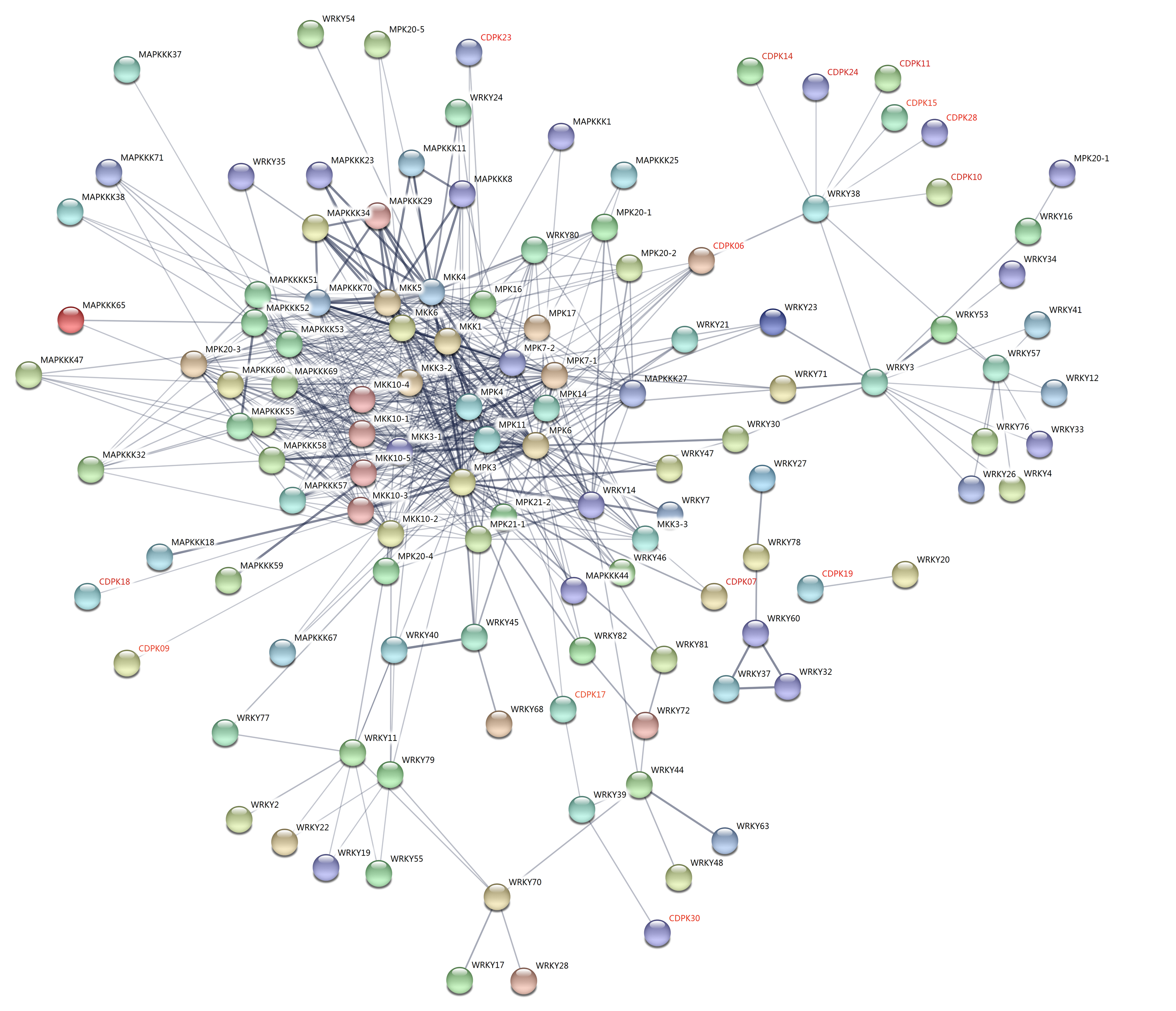

Supplement: Supplementary file 16 — Additional file 16 Predicted protein-protein interaction network of CDPKs, WRKYs and MAPK cascade members identified in B. distachyon. [file 12864_2020_6475_MOESM16_ESM.tif]
