# Supplementary material for: Genome-wide survey and expression analysis of calcium-dependent protein kinase (CDPK) in grass Brachypodium distachyon
Source: BMC Genomics. 2020 Jan 16;21:53. doi: 10.1186/s12864-020-6475-6 (PMC6966850; doi:10.1186/s12864-020-6475-6)
Supplement: Supplementary file 5 — Additional file 5. List of publicly available transcriptome data accession number. [file 12864_2020_6475_MOESM5_ESM.docx]

Additional file 5 List of publicly available transcriptome data accession number

| Treatment | Accession number |
| --- | --- |
| 2,4-D | GSE97940 |
| Cold1 | GSE112296 |
| Drought | GSE112296 |
| Heat | GSE112296 |
| Submergence 1 | GSE112296 |
| Cold 2 | GSE48040 |
| Mowed | GSE101500 |
| Mild-drought | GSE38247 |
| Sever-drought | GSE38247 |
| Submerged 2 | GSE74222 |
