# Supplementary material for: Genome-wide survey and expression analysis of calcium-dependent protein kinase (CDPK) in grass Brachypodium distachyon
Source: BMC Genomics. 2020 Jan 16;21:53. doi: 10.1186/s12864-020-6475-6 (PMC6966850; doi:10.1186/s12864-020-6475-6)
Supplement: Supplementary file 6 — Additional file 6 The CDS and protein sequence of BdCDPKs. [file 12864_2020_6475_MOESM6_ESM.doc]

Additional file 6 The CDS and protein sequence of BdCDPKs.

| Gene name | Type | Sequence |
| --- | --- | --- |
| BdCDPK01 | CDS | ATGGGCAACTGCTGCGGTGCGCCGTCGTCGTCGTCGCAGGGCGGCGGGAAGAACAGGCGGAAGCAGAAGGCGAACCCCTTCACCGTCGCGTACAACCGCGGCGCGGCGACGCCGGCGCGGCCGGGGCTGGTGGTGCTGCGGGACCCGACGGGGCGGGACCTGGAGGCCCGGTACGTGCTGGGCGGGGAGCTGGGCCGCGGCGAGTTCGGCATCACGTACCTGTGCACGGAGGCCGCCACGGGCGCGCGGCTGGCCTGCAAGTCGATTTCCAAGCGGAAGCTGCGGACGCCGGTGGACGTGGAGGACGTGCGCCGGGAGGTGGAGATCATGCGCCACATGCCGCCGCACCCGAACATCGTCAGCCTCAGCGCCGCCTACGAGGACGAGGACGACGTGCACCTCGTCATGGAGCTCTGCGAGGGCGGCGAGCTCTTTGACAGGATCGTCGCTCGAGGGCACTACACAGAGCGCGCTGCCGCTGCCGTAACGCGCACCATCGTCGAGGTGGTCCAGATGTGCCACAGGAATGGTGTCATCCATCGGGACCTTAAGCCAGAAAACTTCTTATATGCAAACAAGAAGGAGAGTTCTCCTCTAAAGGCAATTGATTTTGGGCTATCTGTGTTTTTCAGGCCTGGTGAACGTTTTACTGAAATTGTAGGTAGTCCATACTACATGGCCCCAGAGGTTTTAAAGCGCAACTATGGTCCTGAAATCGATGTCTGGAGTGCAGGAGTGATACTTTACATTCTTCTTTGTGGTGTGCCACCATTTTGGGCAGAAACTGAACAGGGAGTAGCACAGGCAATTATACGTTCTGTGGTTGATTTCAAAAGAGACCCATGGCCAAGAGTATCTGAGCCGGCTAAGGACCTTGTCAGGCGGATGCTGGATCCAAATCCAATCACGCGACTTACTGCAGCACAAGTACTCGAACATCCATGGTTACATGATTCCAAAAAGAATCCTGACATTCCTCTTGGAGATACTGTCCGAGCAAGACTGCAGCAATTTTCTGCAATGAACAAGTTAAAGAAGAAAGCCTTAAGGGTGATTGCTGAGCATTTATCTTTAGAGGAAGTAGCTGATATAAAGAAAATGTTTGATGGCATGGATGTAAACAAAAATGGCAAACTAACCTTTGAGGAGTTCAAGGCTGGCCTCCGTAAACTTGGAAACAAAATGCATGATTCAGATCTTCAGATGTTGATGGATGCTGCTGATCTTGATAAAAATGGCACCCTAGATTATGGTGAATTTGTTACGGTGTCTATACATGTGAGAAAGATAGGCAATGATGAACATATCCAAAAGGCTTTCTCATACTTTGACCGGAATGACAGTGGCTACATAGAAATCGAAGAGCTTAGAGAGGCTTTGACCGATGAGTTTGAGGGACCTGCTGATGAAGACATTATCAATGGCATCATTCATGATGTAGATACTGATAAGGACGGGAAAATAAGCTACGACGAGTTCTCGGCGATGATGAAGGCCGGAACAGACTGGAGGAAAGCATCCAGGCAGTACTCGAGGCAGAGGTTCAGCAACCTGAGCCTGAAGCTTCACAAGGACGGGTCCATCACCGACGATCGGCAGTAG |
|  | Protein | MGNCCGAPSSSSQGGGKNRRKQKANPFTVAYNRGAATPARPGLVVLRDPTGRDLEARYVLGGELGRGEFGITYLCTEAATGARLACKSISKRKLRTPVDVEDVRREVEIMRHMPPHPNIVSLSAAYEDEDDVHLVMELCEGGELFDRIVARGHYTERAAAAVTRTIVEVVQMCHRNGVIHRDLKPENFLYANKKESSPLKAIDFGLSVFFRPGERFTEIVGSPYYMAPEVLKRNYGPEIDVWSAGVILYILLCGVPPFWAETEQGVAQAIIRSVVDFKRDPWPRVSEPAKDLVRRMLDPNPITRLTAAQVLEHPWLHDSKKNPDIPLGDTVRARLQQFSAMNKLKKKALRVIAEHLSLEEVADIKKMFDGMDVNKNGKLTFEEFKAGLRKLGNKMHDSDLQMLMDAADLDKNGTLDYGEFVTVSIHVRKIGNDEHIQKAFSYFDRNDSGYIEIEELREALTDEFEGPADEDIINGIIHDVDTDKDGKISYDEFSAMMKAGTDWRKASRQYSRQRFSNLSLKLHKDGSITDDRQ |
| BdCDPK02 | CDS | ATGGGGAACAACTGCGTCGGCCCCAACGCCCCCGGTCGCAACGGCTTCCTCGCCTCCGTCGCCATCTGGCGCCCCGCCGCCGCGCGCCCCGACCCGGCGGCGCCCGCCCCCGACGCGCCCCCGCAGACGACGTTCTCCGACAAGGCGCCCGAGCCCGTCACCATCCCCGCCTCCGAGCACTCCTCCCACCACTCCTCCCGCGCCGCGGACCCGCCGCCGCCCAAGGCCGCCGAGCCGCAGCCGGCGCAGGCGCAGACGCAGGCACCCGCTCCCGCGGCGAAGAAGCCCGTGCCCAAGGTCAAGCGCGTCCAGAGCGCCGGCCTCCAGGCCGACTCCGTCCTCAAGCGCGACGTCAACACCGCCCGCCTCAAGGACCTCTACACGATCGGGAAGAAGCTCGGGCAGGGCCAGTTCGGCACCACCTACCTCTGCGTCGAGAAGGCCACCGGCAAGGAGTTCGCCTGCAAGTCCATCGCCAAGCGGAAGCTGCTCACGGAGGAGGACGTCGAGGACGTGCGCCGCGAGATCCAGATCATGCACCACCTCGCGGGCCACAGCAATGTCGTGTCCATCGTCGGGGCCTACGAGGACGCCGTCGCCGTGCAGCTCGTCATGGAGCTCTGCGCCGGCGGGGAGCTCTTCGACAGGATCATCCAGAGGGGGCATTACTCCGAGAAGGCCGCGGCCCAGCTCACGCGGGTCATCGTTGGCGTTGTCGAGGCGTGCCACTCGCTCGGGGTGATGCATAGGGATCTCAAGCCGGAGAATTTCCTATTCGTGAACCAGAACGAGGATTCGCCGCTCAAGGCCATTGATTTTGGGCTCTCAATCTTCTTCAAGCCAGGTCAGATGTTTACAGATGTTGTTGGAAGTCCATACTATGTTGCCCCTGAGGTTCTTCTAAAGCACTATGGACGTGAGGTTGATGTCTGGAGTGCCGGTGTAATAATATATATCTTGTTGAGTGGGGTTCCTCCATTCTGGGATGAAAGTGAACAAGGGATATTCGAACAAGTTTTGAAAGGTGAGCTGGACTTTTCAACTGACCCCTGGCCTAGTATCTCAGAGAGTGCAAAGGATTTGGTCAGGAAAATGCTTATCCGTGATCCAAAGAAGAGATTGACTGCTCACGAAGCTCTATGTCACCCTTGGGTTTGTGTTGATGGAGTTGCTCCTGACAAGCCTCTTGATTCTGCTGTCCTAAGTCGGTTAAAACAGTTTTCTGCAATGAACAAACTAAAGAAAATGGCCCTAAGGGTTATTGCGGAGAGTTTATCTGAAGAAGAAATTGCAGGGTTGAAAGAAATGTTCAAAATGCTAGACACTGATAACAGCGGTCATATCACATTGGAGGAGCTAAAATCTGGGTTGCAGCGAGTTGGTGCTACTTTGATGGACTCAGAAATTGATGCTTTAATGGAAGCAGCAGATATCGACAACAGTGGGACAATTGATTACGGGGAATTCATTGCTGCAACTATGCATATAAACAAAGTTGACAAGGAGGATAAGCTATTTACCGCTTTCTCATACTTTGATAAAGATGGCAGTGGTTACATTACTCAAGATGAGCTCCAAAAGGCATGTGAAGAGTTTGGTATAGGAGATACCCATCTCGAGGATATTATTGGAGACGTTGATAAGGACAATGATGGGCAGATCGACTACAATGAATTTGTTGAAATGATGCAGAAAGGTAATAATCCATTGGGTAGAAAGGGACAGCAAAGTAACGTGAACTTTGGTCTTGGGGATGCACTGAAGCTTCGGTAA |
|  | Protein | MGNNCVGPNAPGRNGFLASVAIWRPAAARPDPAAPAPDAPPQTTFSDKAPEPVTIPASEHSSHHSSRAADPPPPKAAEPQPAQAQTQAPAPAAKKPVPKVKRVQSAGLQADSVLKRDVNTARLKDLYTIGKKLGQGQFGTTYLCVEKATGKEFACKSIAKRKLLTEEDVEDVRREIQIMHHLAGHSNVVSIVGAYEDAVAVQLVMELCAGGELFDRIIQRGHYSEKAAAQLTRVIVGVVEACHSLGVMHRDLKPENFLFVNQNEDSPLKAIDFGLSIFFKPGQMFTDVVGSPYYVAPEVLLKHYGREVDVWSAGVIIYILLSGVPPFWDESEQGIFEQVLKGELDFSTDPWPSISESAKDLVRKMLIRDPKKRLTAHEALCHPWVCVDGVAPDKPLDSAVLSRLKQFSAMNKLKKMALRVIAESLSEEEIAGLKEMFKMLDTDNSGHITLEELKSGLQRVGATLMDSEIDALMEAADIDNSGTIDYGEFIAATMHINKVDKEDKLFTAFSYFDKDGSGYITQDELQKACEEFGIGDTHLEDIIGDVDKDNDGQIDYNEFVEMMQKGNNPLGRKGQQSNVNFGLGDALKLR |
| BdCDPK03 | CDS | ATGGGCAACACGTGCGTCGGCCCCAGCATCGCCAAGAACGGCTTCTTCCAGTCCGTCTCCACCGTGCTCTGGAAGGCGCGCCCCGACGGCGACGCTCTCCCCGACCCCGCCGCCGCCGCCTCCAATGGCCCAACAACAACACACCGCCCTCCCCCCGACCCGCCGCTGGCCATCCAGAGCATCCGCAAAGCTCCCGAGCCCGTCAAGATCGCCTCCTCCAAGCCAGAGCAGCAGAATCCCAAACCCTCCTCCGCCGCCGCCGGCGCGGCAGAGCAGGACTCCAAGCAGCAGGCGAGAAGCACGGACTCAGCCTCTACCGCCGATTCCAACTCCATCTCCTCCAACTCCGGCGAGCCCACGAAGCCGCACAACCACCGGCCCAAGGCGCCCCAGGTGAAGCGCGTCTCGAGTGCGGGCCTCCTGGTGGGCTCGGTCCTGAAGCGGAAGACGGAGAGCGTCAAGGAGAAGTACAGCCTGGGCCGGCGGCTGGGCCAGGGCCAGTTCGGCACGACGTACCTGTGCGTGGACCGGGCCTCCGGCAAGGAGTACGCCTGCAAGTCCATCCTCAAGCGGAAGCTGGTGACGGACGACGACGTGGAGGACGTCCGCCGGGAGATCCAGATAATGTACCACCTGGCGGGGCACCCCAACGTGATCTCCATCAAGGCCGCCTACGAGGACGCCGTCGCCGTGCACCTCGTCATGGAGCTCTGCGCCGGCGGCGAGCTCTTTGACAGGATCGTCCAGAAGGGGCATTATACGGAGCGGAAGGCGGCGGAGCTAGCCAGGGTTATCGTGGGTGTTGTTGAGGTGTGCCACTCCATGGGCGTCATGCACCGGGATCTCAAGCCTGAGAACTTCTTGTTTGCGGACCACACGGAGGAGGCCGCGCTCAAGACCATCGACTTTGGACTCTCCGTCTTCTTCCGGCCAGGTCAAGTGTTCACTGACGTTGTCGGAAGCCCTTACTACGTCGCACCGGAAGTCCTGAGGAAGAAATACGGTCCCGAGGCCGATGTCTGGAGTGCCGGTGTGATCATCTACATCCTGCTGTGCGGTGTGCCGCCATTTTGGGCAGAGAACGAGCAGGGTATATTTGAAGAGGTTTTACACGGGAAACTCGACTTCCAGTCAGATCCATGGCCTAGCATCTCTGAAGGCGCCAAAGATCTCGTGAGGAGAATGCTTCTCAGGGACCCCAAGAAACGATTGACCGCTCACGAAGTTTTGCGGCATCCATGGGTTCAGGTTGGTGGTCTGGCTCCTGATAAGCCTCTGGATTCTGCTGTTCTGTCCCGTATGAAGCAGTTCTCGGCCATGAATAAGCTGAAGAAGATGGCTCTTAGGGTGATTGCGGAGAACCTGTCCGAGGACGAGATCGCCGGGCTGAAGGAAATGTTCAAGATGATCGACTCGGACAACAGCGGGCAAATCACCTACGAGGAGCTCAAGGTGGGGCTGAAGAAGGTGGGCGCCAACCTGCAGGAATCGGAGATCTACGCTCTGATGCAAGCGGCGGATGTGGACAACAGCGGGACGATCGACTACGGGGAGTTCATCGCGGCGACGCTGCACCTGAACAAGGTGGAGCGGGAGGACCACCTGTTCGCGGCGTTCCAGTACTTCGACAAGGACGGCAGCGGCTACATCACCCCCGACGAGCTGCAGCTCGCCTGCGAGGAGTTCGGGCTCGGCGCCGACGTCCAGCTCGACGACATGATCAGGGAGGTCGACCAGGACAACGACGGGCGCATCGATTACAACGAGTTCGTGGCGATGATGCAGAAGCCGACGATGGGGCTGCCCAAGTCCAAGGCCGGGCTGGACAGCAGCTTCAGCATCGGGTTCAGGGAGGCGCTGAGGATGTCTTAG |
|  | Protein | MGNTCVGPSIAKNGFFQSVSTVLWKARPDGDALPDPAAAASNGPTTTHRPPPDPPLAIQSIRKAPEPVKIASSKPEQQNPKPSSAAAGAAEQDSKQQARSTDSASTADSNSISSNSGEPTKPHNHRPKAPQVKRVSSAGLLVGSVLKRKTESVKEKYSLGRRLGQGQFGTTYLCVDRASGKEYACKSILKRKLVTDDDVEDVRREIQIMYHLAGHPNVISIKAAYEDAVAVHLVMELCAGGELFDRIVQKGHYTERKAAELARVIVGVVEVCHSMGVMHRDLKPENFLFADHTEEAALKTIDFGLSVFFRPGQVFTDVVGSPYYVAPEVLRKKYGPEADVWSAGVIIYILLCGVPPFWAENEQGIFEEVLHGKLDFQSDPWPSISEGAKDLVRRMLLRDPKKRLTAHEVLRHPWVQVGGLAPDKPLDSAVLSRMKQFSAMNKLKKMALRVIAENLSEDEIAGLKEMFKMIDSDNSGQITYEELKVGLKKVGANLQESEIYALMQAADVDNSGTIDYGEFIAATLHLNKVEREDHLFAAFQYFDKDGSGYITPDELQLACEEFGLGADVQLDDMIREVDQDNDGRIDYNEFVAMMQKPTMGLPKSKAGLDSSFSIGFREALRMS |
| BdCDPK04 | CDS | ATGGGCAACGCTTGCTTGTTCTGCTGCACCACCACCACCTCGCCTCAGCCACACGCGCCCGAGTCGGCGGAAGCACCGCCGCCGCCGCCGTATACCAGGAAGAAGAAGCCGCAGACGACACCATCCACGAGCCAAAGCCACAGCCAGGGCAAACCAAGCCCCACCGCCGCCAGGGCCAGCCCTAACCCGAAGCCCAGACCTAGCCGACCCAGGCCCAAGCCCAACCCATACGCAGCGGCGTCGCCGTCCCGCGGCCCCGGCGGCCCGCGGGTGCTGGACGGCGTGGTGCCGCACCACCCGCGCGGGCCACGCGTGACGGACAAGTACCACCTGGGCCGCGAGCTGGGCCGGGGCGAGTTCGGCGTGACCAGGCTCGCCACGGACCGCGGCCCGTCACGGGAGCGGCTGGCCTGCAAGTCCATCCCCAAGGCCCGGCTCCGGACGGCCGTGGACGTGGCCGACGTGCGCCGGGAGGTGTCCATCATGGCGTCGCTCCCCGACCACCCGGCGCTCGTGCGGCTCCGGGCCGCCTACGAGGACGTGGACGCCGTGCACCTCGTCATGGAGCTCTGCGACGGCGGGGAGCTGTTTGACAGGATCGTGGCCCGCGGGCGGTACACGGAGCGCGCCGCCGCGGCCGCGGCCAGGACCGTGGCCGAGGTTGTGCGCGCGCTGCACGCCGCCGGGGTCGTGCATCGGGACCTCAAGCCGGAGAATTTTTTGTATAAGGGGAAGAGCGAGGACGCGCAGCTCAAGGCCATCGACTTTGGACTCTCCGTCTTCTTCCGGCCAGCTGCCGGGGACTGTGGCCGGGGTGCAATGTTCACCGGACACGCCGCGCGCCTCATCGTCAGGTTGAGTGCTTGGTCGCGAAAAAAGAAAATAACTGAGCAAAATGCAACAGGCGAGCGGTTCACGGAGATCGTGGGCAGCCCGTACTACATGGCCCCGGAGGTGCTCCGCCGTAACTACGGCCCCGAGGTGGACATCTGGAGCGCCGGCGTCATCCTCTACATCCTCCTCTGCGGCGTCCCTCCCTTCTGGGCCGAGACGGAGCAGGGCGTGGCGCGCTCCATCCTGCGCGGCGTGGTGGACTTCCAGCGCGAGCCCTGGCCCCGGATCTCCGACAGCGCCAAGAGCCTCGTGCGCCAGATGCTCGAGATGGACCCAAAGAAGCGCCTCACCGCCCGCCAAGTCCTCGAGCACCCGTGGCTGCAGGACGCGAAGAAGGCGCCGAACGTGCCGCTGGGCGACATCGTGCGCGCGAGGCTGAAGCAGTTCTCGGTGATGAACCGGTTCAAGAAGAAGGCGATGCGGGTGATCGCGGAGCACCTGTCGGCGGAGGAGGTGGAGGTGATCAAGGAGATGTTCGCGCTCATGGACACGGGCAAAAACGGCCGGGTCACGCTCCAGGAGCTCAAGGCCGGCCTCACCAAGGTCGGCTCCAAGCTCGCCGAGCCCGAGATGGAGCTGCTCATGGAGGCCGCTGACGTGGATGGCGACGGGTACCTTGACTACGCCGAGTTCGTGGCCATCACCATCCACCTGCAGCGGCTCTCCAATGACGAGCACCTGCGCACGGCGTTCCTCTTCTTCGACAAGGACAGCAGCGGCTACATCGAGCGCCAGGAGCTGGCCGACGCGCTCGCCGACGACAACGGACAGGCCAACCACGCCGTCGTCGACCACGTCCTGCAAGAGGTGGACACGGACAAGGATGGCCGGGTCAGCTTCGAGGAGTTCGTGGCCATGATGAAGTCCGGGACGGACTGGCGGAAGGCGTCCAGGCAGTACTCCAGGGAGCGGTTCAAGACGCTCAGCAACAGCCTCATCAAGGACGGATCGATCTCCATGGCACGCTAA |
|  | Protein | MGNACLFCCTTTTSPQPHAPESAEAPPPPPYTRKKKPQTTPSTSQSHSQGKPSPTAARASPNPKPRPSRPRPKPNPYAAASPSRGPGGPRVLDGVVPHHPRGPRVTDKYHLGRELGRGEFGVTRLATDRGPSRERLACKSIPKARLRTAVDVADVRREVSIMASLPDHPALVRLRAAYEDVDAVHLVMELCDGGELFDRIVARGRYTERAAAAAARTVAEVVRALHAAGVVHRDLKPENFLYKGKSEDAQLKAIDFGLSVFFRPAAGDCGRGAMFTGHAARLIVRLSAWSRKKKITEQNATGERFTEIVGSPYYMAPEVLRRNYGPEVDIWSAGVILYILLCGVPPFWAETEQGVARSILRGVVDFQREPWPRISDSAKSLVRQMLEMDPKKRLTARQVLEHPWLQDAKKAPNVPLGDIVRARLKQFSVMNRFKKKAMRVIAEHLSAEEVEVIKEMFALMDTGKNGRVTLQELKAGLTKVGSKLAEPEMELLMEAADVDGDGYLDYAEFVAITIHLQRLSNDEHLRTAFLFFDKDSSGYIERQELADALADDNGQANHAVVDHVLQEVDTDKDGRVSFEEFVAMMKSGTDWRKASRQYSRERFKTLSNSLIKDGSISMAR |
| BdCDPK05 | CDS | ATGGGCAACTGCTGCGTGACGGCGGGGGAAGGCGGCGGGAGTGGCAGGAAGAAGCAGCCCAAGGAGCCGAAGCAGAAGAAGGGCAAGAAGCCCAACCCTTTCTCGATCGAGTACAACCGGTCGGCGCCGCCGGGGGCCACGAAGCTGGTGGTGCTGCGGGAGCCCACGGGGCGGGACATCGCCGCGCGGTACGAGCTGGGCGGGGAGCTAGGCCGCGGGGAGTTCGGGGTCACCTACCTCTGCACGGACCGCGCCACAGGGGAGGCCCTCGCCTGCAAGTCCATCTCCAAGAAGAAGCTCCGGACAGCGGTGGACATCGAGGACGTGCGCCGCGAGGTTGAGATCATGCGCCACCTCCCCAAGCACCCCAACATCGTCACCCTCAGGGACACGTACGAGGACGACAATGCCGTGCACCTCGTCATGGAGCTCTGCGAGGGCGGGGAGCTCTTCGACCGGATCGTCGCACGGGGGCACTACACCGAGCGCGCCGCCGCAGTGGTTACCAAAACCATCGTCGAGGTCGTGCAGATGTGCCATAAGCATGGGGTGATGCACCGGGACCTCAAACCAGAGAATTTCTTGTTTGCAAACAAGAAAGAAACCGCGGCACTTAAGGCAATTGATTTTGGCCTCTCTGTATTTTTCACTCCAGGTGAACGGTTCACTGAGATTGTTGGAAGTCCTTATTACATGGCTCCGGAGGTGTTGAAGAGAAACTATGGCCAAGAGGTTGATGTTTGGAGTGCAGGAGTGATTCTCTACATTCTTCTTTGTGGTGTTCCTCCATTCTGGGCAGAAACTGAACAAGGTGTTGCTCAAGCAATTATCCGTTCTGCCATTGACTTTAAAAGGGATCCATGGCCAAGGGTCTCTGATAATGCAAAGGACCTTGTCAGGGGAATGCTTAACCCAGATCCAAAGCGGCGATTAACAGCCCAGCAAGTACTTGATCATCCGTGGTTGCAAAATATTAAGAAGGCACCAAATGTCAATTTGGGTGAAACTGTCAAGGCCAGACTTCAACAATTCTCTGTGATGAACAAGTTCAAGAAGCATGCACTTAGGGTCATAGCTGAGCATCTTTCGGTAGAGGAGGTGGCTGGCATAAAGGATATGTTTGAAAAGATGGACCTTAACAAAGATAGTATGATTAATTTTGATGAGCTGAAGCTTGGTTTGAATAAGCTTGGACACCAAATGCCTGATGCAGATGTCCAAATACTAATGGATGCTGCGGACGCTGATGGAAATGGATGCTTAGACTATGGAGAATTTGTTACTCTGTCCGTTCACCTAAAAAAGATTGGCAATGACGAGCATCTGCATAAGGCATTTGCATACTTTGATCGGAACAAGAGTGGATATATTGAAATTGACGAGCTCCGCGAGTCATTAGCGGATGACCTGGGACACAATCATGAAGAGGTTATCAATGCCATCATCCGCGATGTGGACACTGATAAGGATGGCAAGATAAGCTTTGATGAGTTTGTGGCAATGATGAAGGCTGGAACGGACTGGAGGAAAGCCTCGAGACAGTATTCCAGAGAACGGTTCACTAGCCTTAGCTTAAAGCTGCAGAAGGATGGATCGTTGCAGATAACAACCCAATAG |
|  | Protein | MGNCCVTAGEGGGSGRKKQPKEPKQKKGKKPNPFSIEYNRSAPPGATKLVVLREPTGRDIAARYELGGELGRGEFGVTYLCTDRATGEALACKSISKKKLRTAVDIEDVRREVEIMRHLPKHPNIVTLRDTYEDDNAVHLVMELCEGGELFDRIVARGHYTERAAAVVTKTIVEVVQMCHKHGVMHRDLKPENFLFANKKETAALKAIDFGLSVFFTPGERFTEIVGSPYYMAPEVLKRNYGQEVDVWSAGVILYILLCGVPPFWAETEQGVAQAIIRSAIDFKRDPWPRVSDNAKDLVRGMLNPDPKRRLTAQQVLDHPWLQNIKKAPNVNLGETVKARLQQFSVMNKFKKHALRVIAEHLSVEEVAGIKDMFEKMDLNKDSMINFDELKLGLNKLGHQMPDADVQILMDAADADGNGCLDYGEFVTLSVHLKKIGNDEHLHKAFAYFDRNKSGYIEIDELRESLADDLGHNHEEVINAIIRDVDTDKDGKISFDEFVAMMKAGTDWRKASRQYSRERFTSLSLKLQKDGSLQITTQ |
| BdCDPK06 | CDS | ATGGGGCAGTGTTGCAGCAGAGCTACGTCCCCTGATTCTGGTCGAGGAGTCAATGGTTATGGCTATTCCCACCAGCCAAAACAAGCGCAAACACCTCCTAGTTACAACCATGCTCATCCACCACCACAAGCCGAGGTAAGGTACACACCGCCAGCGATGAACCCTCCGGTAGTCCCACCTGTGGTTGCCCCCTCAAAGCCCACACCAGACACGATTCTTGGGAAGCAGTACGAGGATGTGAGATCCGTCTACTCCCTTGGTAAGGAACTTGGCCGTGGACAGTTTGGGGTGACTTACCTTTGCACTGAGATTGCCACTGGCAGGCAGTATGCTTGCAAGTCCATATCCAAGCGCAAGCTCGTGAGTAAGGCCGACAAGGAGGATATTCGCAGGGAGATCCAGATCATGCAGCACCTATCTGGACAACCGAACATAGTCGAGTTCTGCGGAGCATATGAGGACAAGAGCAGTGTGCATGTTGTCATGGAGCTCTGTGCAGGTGGGGAGCTGTTTGATCGGATTATTGCTAAGGGGCACTACTCAGAACGAGCAGCTGCTACAATCTGCAGAGGGGTTGTGAATGTTGTCAATGTTTGCCATTTCATGGGAGTGATGCACCGTGATCTGAAGCCAGAGAACTTCTTGCTTGCGACCAAGGATGAGAATGCAGTGCTCAAGGCCACTGATTTTGGCCTCTCAGTCTTCATTGAAGAAGGAAAAATGTATAGAGACATCGTTGGAAGTGCTTATTATGTTGCTCCTGAAGTCCTTAAACGAAATTATGGTAAAGAGATAGATGTTTGGAGTGCAGGTGTTATTCTGTACATTCTTCTCAGTGGTGTTCCTCCATTCTGGGCTGAAACTGAGAAGGGAATATTTGATGCTATTCTTCAAGGGGACATTGATTTCGAAAGTCAGCCATGGCCATCAATTTCTGAGAGCGCTAAAGACCTTGTTAGAAAGATGTTGGCACAGGATCCAAAGAAAAGAATTAATTCAGCACAAGTTCTTCAACATCCATGGCTCAGAGAAGGAGAAGCATCAGATAAACCTATCGACAGTGCTGTTCTTTCTAGGATGAAACAATTCAGAGCTATGAATAAGCTGAAAAAGATGGCTCTGAAGGTTATAGCTTCAAACCTTAACGAGGAAGAGATCAAGGGCTTGAAACAAATGTTCAGTAACATGGACACAGACAACAGTGGGACAATCACATACGAAGAACTCAAAGCAGGATTGGCCAAACTTGGATCAAAGCTATCAGAAGCTGAAGTAAAGCAGTTGATGGATGCCGCTGATGTGGACGGCAGTGGATCAATTGACTATGTTGAGTTCATCACAGCCACAATGCATAGACACAAGCTCGAAAGAGATGAGCATTTGTTCAAAGCATTCCAGTATTTTGACAAAGACAGTAGTGGCTTCATTACAAGAGATGAACTGGAAACTGCTTTGATTGAGCATGAAATGGGAGACACGGATACCATAAAGGACATCATATCAGAAGTTGACACGGATAACGATGGGAGGATTAACTATGACGAATTCTGCGCAATGATGAGAGGAGGAATACAGCAGCAGCCAGTAAGGCTCAAGTAG |
|  | Protein | MGQCCSRATSPDSGRGVNGYGYSHQPKQAQTPPSYNHAHPPPQAEVRYTPPAMNPPVVPPVVAPSKPTPDTILGKQYEDVRSVYSLGKELGRGQFGVTYLCTEIATGRQYACKSISKRKLVSKADKEDIRREIQIMQHLSGQPNIVEFCGAYEDKSSVHVVMELCAGGELFDRIIAKGHYSERAAATICRGVVNVVNVCHFMGVMHRDLKPENFLLATKDENAVLKATDFGLSVFIEEGKMYRDIVGSAYYVAPEVLKRNYGKEIDVWSAGVILYILLSGVPPFWAETEKGIFDAILQGDIDFESQPWPSISESAKDLVRKMLAQDPKKRINSAQVLQHPWLREGEASDKPIDSAVLSRMKQFRAMNKLKKMALKVIASNLNEEEIKGLKQMFSNMDTDNSGTITYEELKAGLAKLGSKLSEAEVKQLMDAADVDGSGSIDYVEFITATMHRHKLERDEHLFKAFQYFDKDSSGFITRDELETALIEHEMGDTDTIKDIISEVDTDNDGRINYDEFCAMMRGGIQQQPVRLK |
| BdCDPK07 | CDS | ATGGGCCTGTGCTCCTCCTCTAGCGCCGCCAGCCCCGCCGATCCCGCCGGCGGCAACGGTAATAAGGAGAAGGGGAGGAAGGGCAGCGGCAGCAGGGGGATCGTGGCGTGCGGGAAGCGGACGGACTTCGGTTACGACAAGGACTTCGAGGCGCGGTATACGATCGGGAAGCTTCTTGGCCACGGACAGTTCGGCTACACCTTCGCCGCCGTCGACCGCTACTCCGACGAGCGCGTCGCCGTCAAGCGCATCGACAAGAACAAGATGGTCCTTCCTGTTGCTGTTGAAGATGTAAAGCGAGAAGTTAAAATACTCAAGGCCTTACAAGGGCATGAAAATGTTGTACATTTTTACAATGCATTCGAGGATGATAACTATGTGTATATTGTTATGGAATTATGTGAGGGTGGTGAGTTGCTTGACCGCATATTAGCCAAGAAAGATAGCCGTTATAGCGAGAAAGATGCTGCAGTAGTTGTGCGACAAATGCTCAAGGTTGCAGCTGAATGCCATTTGCATGGTTTGGTTCATCGGGACATGAAACCCGAGAACTTCCTCTTCAAATCATCAAAGGAGGGCTCACCCCTCAAGGCTACAGATTTTGGCCTTTCGGACTTCATAAGAGCAGGGAAGCAATTTCGTGACATTGTCGGAAGTGCCTACTATGTAGCACCAGAAGTGCTCAAGCGTAAGTCAGGGCCGGAATCTGATGTTTGGAGTATTGGTGTTATAACTTATATTCTGTTGTGCGGAAGACGGCCTTTTTGGGACAAAACTGAAGATGGAATCTTTAAAGAGGTGTTAAAAAACAAGCCAGATTTCCGTCGCAAGCCTTGGACGAACATTACTCCAAGTGCTAAAGATTTTGTACAGAAGTTACTAGTTAAGGATCCCCGTGCAAGACTAACTGCTGCCCAGGCATTATCACATGAATGGGTGAGAGAAGGAGGACAGGCATCTGAAATACCTCTGGATATATCAGTATTGCATAATATGCGACAGTTTGTGAAATACAGTCGTTTCAAGCAATTTGCTTTAAGGGCTTTGGCATCTACACTAAATTCAGAAGAACTGTCTGATCTTCGCGACCAGTTCAATGCCATTGATATTGACAAGAGTGGAACGATAAGCCTAGAAGAACTGAAGCAGGCTCTTGCAAAGGATGTTCCGTGGAGACTAAAGGGTCCTCGTGTTCTAGAGATTGTCGAGGCGATCGACAGTAACACAGATGGGTTAGTTGATTTTGAGGAGTTTGTTGCTGCCACACTACATGTGCATCAGCTGGTGGAACATGACAGCGAGAAGTGGAAGTCATTGTCTCAAGCTGCGTTTGATAAATTCGATGTTGACGGAGATGGCTATATCACATCTGATGAACTGAGAATGAATACAGGACTGAAGGGTTCTATTGACCCCCTCCTGGAGGAGGCTGACATTGACAAAGATGGCAAAATAAGCCTTGACGAATTTCGTAGACTCTTGAAAACTGCAAGCATGAGTTCTCGCAACGCAACCCCAAAAAGCGTTTCCAAGTCATATAGATTTGCGTAG |
|  | Protein | MGLCSSSSAASPADPAGGNGNKEKGRKGSGSRGIVACGKRTDFGYDKDFEARYTIGKLLGHGQFGYTFAAVDRYSDERVAVKRIDKNKMVLPVAVEDVKREVKILKALQGHENVVHFYNAFEDDNYVYIVMELCEGGELLDRILAKKDSRYSEKDAAVVVRQMLKVAAECHLHGLVHRDMKPENFLFKSSKEGSPLKATDFGLSDFIRAGKQFRDIVGSAYYVAPEVLKRKSGPESDVWSIGVITYILLCGRRPFWDKTEDGIFKEVLKNKPDFRRKPWTNITPSAKDFVQKLLVKDPRARLTAAQALSHEWVREGGQASEIPLDISVLHNMRQFVKYSRFKQFALRALASTLNSEELSDLRDQFNAIDIDKSGTISLEELKQALAKDVPWRLKGPRVLEIVEAIDSNTDGLVDFEEFVAATLHVHQLVEHDSEKWKSLSQAAFDKFDVDGDGYITSDELRMNTGLKGSIDPLLEEADIDKDGKISLDEFRRLLKTASMSSRNATPKSVSKSYRFA |
| BdCDPK08 | CDS | ATGGGGAACACCTGCGCCGGCCCCAGCGCCGCCCCGGACCGCCATGGCTTCTTCGACAACGTGTCCGTCGCCATGCTCTGGCGCCCCGGCGGCGCCCGCGCCGAGCCGGTGGTCCCGCCGCCGCCGCCCGACTCGTGCTCTTCCATGTCGTCTTCAACCTCGTCCACGGCCCCCGATCCCGTGACCATCGCCGACTCCGACTACCGCCCGGGCTCCTCTAACCCTAACAAGCCCAGGGTGAAGCGCGTCCAGAGCGCCGGCCTGAGTGCCGAGTCCGTCCTCAAGCGCGACTCGGAGCGCATCAAGGACCTGTACACCCTAGGGAAGAAGCTGGGGCAGGGCCAGTTCGGCACCACCTACCAGTGCGTCGAGAAGGCCACGGGGAAGGAGTTCGCCTGCAAGTCCATCGCCAAGAGGAAGCTCGTCACCGAGGAGGACGTCGAGGACGTGCGCCGCGAGATCCAGATCATGCACCACCTTGCCGGCCACCCTAACGTGATCTCCATTGTCGGGGCGTATGAGGATGCCGTCGCCGTGCACCTTGTCATGGAGCTCTGCGCGGGAGGAGAGCTGTTCGACAGGATCATACAGCGGGGGCACTACTCTGAGAAGGCCGCCGCACAGCTGGCTAGGGTCATCATTTCGGTCGTCGAATCGTGCCATTCTCTTGGGGTCATGCACAGAGACCTCAAACCGGAGAATTTCTTGTTTGTGAACCAGAAAGAGGACTCGCTGCTCAAGGCCATCGATTTTGGCCTATCCATTTTCTTCAAGCCAGGTGAAATTTATTCGGATGTCGTTGGAAGTCCTTACTATGTCGCACCTGACGTTCTGATGAAGAGCTATGGCTGTGAAGTAGATGTTTGGAGTGCCGGTGTAATAATTTATATCTTGTTGAGTGGGGTCCCTCCATTTTGGGATGAATCTGAACAAGGGATATTTGAACAAGTTTTGAAAGGTGATCTTGATTTTTCATCGGAGCCCTGGCCCAGTATCTCAAAGAGTGCAAAGGATTTGGTCAGGAAAATGCTAAACCGTGATCCCAGGAAGAGATTGACTGCACATGAAGCTCTATGTCATCCTTGGGTTTGTGTTGATGGAGTTGCTCCTGACAAACCTCTTGATTCTGCTGTCTTAACTAGATTAAAACAATTTTCAGCAATGAACAAACTAAAAAAGATGGCCCTTAGGGTAATTGCCGAGAATCTGTCTGAAGATGAAATTGCAGGATTGAAAGAAATGTTCAAAATGCTGGACACTGACAATAGTGGTCAGATCACATTGGAGGAACTAAAAATTGGCTTGCACAGAGTCGGTGCTAACTTAAAGGAGTCAGAAATTGCAACTCTAATGGAAGCGGCGGATATTGATAACAGTGGTTCAATTGATTATGGGGAGTTCCTTGCTGCAACTTTGCATCTGAACAAGGTGGAGAGAGAAGATAATCTCTTTGCAGCATTCTCATACTTCGATAAGGATGGCAGTGGGTACATTACTCAAGATGAACTGGAAAAAGCCTGTGAAGAGTTTGGTATAGGAGACGCACATCTTGATGATATTATCCGAGACATTGATCAGGACAATGATGGCCGGATCGACTACAATGAATTTGTAACAATGATGCAGAAGGGAAATAATCCACTAGGGAAAAAGGGACAGGGGCAAATGAGCTTTGGTCTTAGGGAAGCATTGAAGATTGGCTAA |
|  | Protein | MGNTCAGPSAAPDRHGFFDNVSVAMLWRPGGARAEPVVPPPPPDSCSSMSSSTSSTAPDPVTIADSDYRPGSSNPNKPRVKRVQSAGLSAESVLKRDSERIKDLYTLGKKLGQGQFGTTYQCVEKATGKEFACKSIAKRKLVTEEDVEDVRREIQIMHHLAGHPNVISIVGAYEDAVAVHLVMELCAGGELFDRIIQRGHYSEKAAAQLARVIISVVESCHSLGVMHRDLKPENFLFVNQKEDSLLKAIDFGLSIFFKPGEIYSDVVGSPYYVAPDVLMKSYGCEVDVWSAGVIIYILLSGVPPFWDESEQGIFEQVLKGDLDFSSEPWPSISKSAKDLVRKMLNRDPRKRLTAHEALCHPWVCVDGVAPDKPLDSAVLTRLKQFSAMNKLKKMALRVIAENLSEDEIAGLKEMFKMLDTDNSGQITLEELKIGLHRVGANLKESEIATLMEAADIDNSGSIDYGEFLAATLHLNKVEREDNLFAAFSYFDKDGSGYITQDELEKACEEFGIGDAHLDDIIRDIDQDNDGRIDYNEFVTMMQKGNNPLGKKGQGQMSFGLREALKIG |
| BdCDPK09 | CDS | ATGGGTAATCAAAATGGGACCCTTGGGACCGATTGCTACCACAATCGGTACCCCAGGGCGCGTCCTGTTTCTGTATATTCAGATGGGTACGTTGAGGGGGGCGGCTACTTGGACTTGAAGAAGCCCTTACCAGAGGCGAACTCGCTTAAGCCCAGCGCTGCTGGTATTCTGAGGCAAGGATTGGATCCGACATCTATATCTGTGCTTGGGCGGAAGACGGCAGATCTAAGGCAGAATTATATCCTTGGTCGGAAGCTTGGGCAGGGTCAGTTTGGCACGACATACCTCTGCACTGAGATTAGTACAGGGTGTGAATATGCATGCAAGACCATCTTAAAGCGCAAGCTCATCACCAAGGTGGATGTTGAGGATGTGCGCCGTGAGATACAGATAATGCACCATTTGTCAGGACACAAGAATGTTGTTTCAATCAAGGATGTTTATGAGGACGGGCAGGCAGTGCACATAGTGATGGAGCTCTTGGCTGGTGGGGAGCTCTTTGATCGAATCAAGGGGAAAGGATATTACAGTGAACTGAAGGCTGCAGAGATTATAAAAATTGTTATTAGCATTGTGGCTATGTGCCATTCACTTGGGGTGATGCACCGCGATCTCAAGCCAGAAAATTTCCTCCTTTTGGATAAAGATGATGACCTGTCAATAAAGGCGATTGATTTTGGTCTATCCATTTTCTTCAAACCAGGGCAGGTTTTCAGTGAGCTGGTTGGGAGCCCATACTATCTTGCTCCTGAGGTATTGAATAAACGTTATGGACCAGAATCTGATGTGTGGTCAGCTGGTGTGATACTCTATGTATTGCTGAGTGGGGTCCCACCATTTTGGTCAGCAGAGACACCTCAAGGAATATTTGATGCAGTTCTTAAGGGGCACATTGATTTTGAATCAGAACCTTGGCCTAAGATATCTGACAGTGCAAAGGATCTTATAAGAAAGATGCTCTGCCATTGTCCTTCAGAGCGCTTGAAAGCCCATGAAGTGCTGCGGCATCCCTGGATCTGTAAAAATGGTGTGACCACTGGTCAGGCCTTGGATCCCAGCATTATCTCTCAGCTTAATGAGTTCTCTGCAATGAAAAATCTAAAGAAGTTGGCTCTGAGGGTGATAGCTGAACGTCTTTCGGAAGAGGAGATTGCTGGATTGAGAGAAATGTTCAAGGCAGTGGACATAAACAATAGAGGTGTAATCACTTTTGGTGAGCTTAGAAAAGGTTTAACAAGATATAGCAATGAATTGGAAGATGCCAAGATTAGTGATATAATGGAAATGGCTGATAGAGACGATAATGTAACCATCAATTATGAAGAATTTATTGCTGCAACCATGCCTCGTAACAAGATAGAATGTGAAGAACACTTGATGGCAGCTTTTACATATTTTGACAAAGATGGTAGTGGTTATATCACAATTGACAAGCTTCAACGAGCTTTTGGAGACCATAACATGGAGGTCACTTTCCTTGAAGAGATTATTTTAGAGGTCGACCAAAACAATGACGGTCAAATTGATTATGCCGAATTTGTAGCCATGATGCAAGGCAACAACAGTACTGGAGATGGGTGTCAAAAACTGGAAACCAATTCGAATGTAACCTTAAGAGACGCACCCCAACTAGATGGACCTAAAGTACATTGA |
|  | Protein | MGNQNGTLGTDCYHNRYPRARPVSVYSDGYVEGGGYLDLKKPLPEANSLKPSAAGILRQGLDPTSISVLGRKTADLRQNYILGRKLGQGQFGTTYLCTEISTGCEYACKTILKRKLITKVDVEDVRREIQIMHHLSGHKNVVSIKDVYEDGQAVHIVMELLAGGELFDRIKGKGYYSELKAAEIIKIVISIVAMCHSLGVMHRDLKPENFLLLDKDDDLSIKAIDFGLSIFFKPGQVFSELVGSPYYLAPEVLNKRYGPESDVWSAGVILYVLLSGVPPFWSAETPQGIFDAVLKGHIDFESEPWPKISDSAKDLIRKMLCHCPSERLKAHEVLRHPWICKNGVTTGQALDPSIISQLNEFSAMKNLKKLALRVIAERLSEEEIAGLREMFKAVDINNRGVITFGELRKGLTRYSNELEDAKISDIMEMADRDDNVTINYEEFIAATMPRNKIECEEHLMAAFTYFDKDGSGYITIDKLQRAFGDHNMEVTFLEEIILEVDQNNDGQIDYAEFVAMMQGNNSTGDGCQKLETNSNVTLRDAPQLDGPKVH |
| BdCDPK10 | CDS | ATGGGCGCCCGCGCCTCCCGCCACCGCCACCAATCCCCCTCCCAATCCCAATCCAGCGACGACTCCCAGTCCCACCACAAGCCACGACCCAAGCCCAAGCCCAAGCATCACCACCAACCACCACAACCCCCTCCGCGGCATCAGCCCCGCCACCACCACCACCCACCCCCGCAACGCCCTCCGCCGCAACATCAACAACAACACCAGCAGCAGCACCATGCGGCGGCGGCGGCGGAGGACACGGGCCGGGTCCTGGGCCGCCCGCTGTCGGACGTGCGCGCGACCTACACGTTCGGCCGGGAGCTGGGCCGGGGCCAGTTCGGGGTCACCTACCTAGCAACCCACAAGTCCACCGGCGCGCGCTACGCCTGCAAGTCGATCTCCGCCCGGAAGCTGTCCCGCGGGGACGACGCCGACGACGTCCGCCGCGAGGTGCAGATCATGCACCACCTCACGGGCCACCGCAGCATCGTCGAGCTCAAGGGCGCCCACGAGGACCGCCACTCGGTCAACCTCGTCATGGAGCTCTGCGAGGGCGGGGAGCTCTTCGACCGCATCATCGCCCGGGGACACTACTCCGAGAGCGCCGCGGCGGCGGTCTGCAGGGAGGTGGTTAATGTCGTCCACTGCTGCCATTCCATGGGGGTCATGCATCGGGACCTCAAGCCGGAGAACTTTTTGTTTCTGAATAAGCGGGAGGACTCGCCGCTTAAGGCTACTGATTTTGGTCTCTCCGTCTTCTTCAAGCCCGGGGAGCAGTTCAGGGATCTCGTTGGGAGCGCCTACTATGTGGCTCCGGAGGTGCTAAAGCGGCGATATGGAGCTGAGGCGGACATATGGAGTGCTGGAGTCATCCTCTACATTCTCCTCTCCGGTGTCCCTCCTTTCTGGGCAGAGAATGAGGATGGTATATTCGATGCTGTTCTGCAAGGTCATATTGATTTCTCATCTGATCCCTGGCCATCAATTTCACACGGGGCTAAAGACTTGGTCAGGAGGATGCTGCGGCAGGACCCCAAGGAGCGCCTCACTGCTGCTGAAATTTTGAACCATCCATGGATTAGAGAGGACGGAGAGGCCCCAGATAAACCACTTGATATTACAGTGATCAGTAGAATGAAGCAGTTCAGAGCAATGAACAAACTTAAGAAGGTTGCCTTGAAGATTGTTGCAGAAAGCTTGTCAGAAGAAGAGATCGTGGGCTTAAAACAAATGTTCAAATCCCTGGATACTGACAACAGCGGGACAATTACTCTTGAAGAACTACGGGCTGGATTACCAAAGCTCGGTACCAAAATTACTGAATCAGAAATAAGACAGTTGATGGAGGCAGCTGATGTTGATGGAAATGGGACCATTGATTATGTTGAATTCATATCGGCAACGATGCACATGAATAGACTAGAGAAGGAGGATCACATATTTAAAGCATTTGAATATTTTGACAAGGACCACAGTGGCTACATAACAGTTGATGAGTTGGAAGAAGCCTTGAAGAAGTATGATATGGGTGATGAGGCAACAATCAAGGAAATCATTGCTGAAGTGGATACAGATCATGATGGGAAAATAAACTACCAAGAGTTTGTTGCCATGATGAAAAACAATAGCCCAGAGATTGTTCCAAATCGACGCCGCTTGTTTTAA |
|  | Protein | MGARASRHRHQSPSQSQSSDDSQSHHKPRPKPKPKHHHQPPQPPPRHQPRHHHHPPPQRPPPQHQQQHQQQHHAAAAAEDTGRVLGRPLSDVRATYTFGRELGRGQFGVTYLATHKSTGARYACKSISARKLSRGDDADDVRREVQIMHHLTGHRSIVELKGAHEDRHSVNLVMELCEGGELFDRIIARGHYSESAAAAVCREVVNVVHCCHSMGVMHRDLKPENFLFLNKREDSPLKATDFGLSVFFKPGEQFRDLVGSAYYVAPEVLKRRYGAEADIWSAGVILYILLSGVPPFWAENEDGIFDAVLQGHIDFSSDPWPSISHGAKDLVRRMLRQDPKERLTAAEILNHPWIREDGEAPDKPLDITVISRMKQFRAMNKLKKVALKIVAESLSEEEIVGLKQMFKSLDTDNSGTITLEELRAGLPKLGTKITESEIRQLMEAADVDGNGTIDYVEFISATMHMNRLEKEDHIFKAFEYFDKDHSGYITVDELEEALKKYDMGDEATIKEIIAEVDTDHDGKINYQEFVAMMKNNSPEIVPNRRRLF |
| BdCDPK11 | CDS | ATGGGCAACTGCTGCCCGGGGTCGAAGGACGCCGATCCTTCGGACGGCGGCAACCCCGGCGAGGGCAGCTCCAACAACGCGCCTTCCGCCTCGGGGTCAGCATCCTCTGCTCCGGCCCAGAACAAGCCGCCGGCGCCCATCGGGCCGGTGCTTGGCCGGCCCATGGAGGACGTGAAGAGCATCTACAACGTCGGGAAAGAGCTCGGCCGCGGCCAGTTCGGGGTCACCTCGCTGTGCACGCACAAGGCGACGGGCCACAAGTTCGCGTGCAAGACCATCAGCAAGCGGAAGCTGTCCACCAAGGAGGACGTGGAGGACGTCCGGCGCGAGGTGCAGATCATGTACCATCTGGCCGGCCAGCCCGGCGTGGTGGAGCTCAAGGGCGCCTACGAGGACAAGCACTCGGTGCACCTGGTCATGGAGCTCTGCGCCGGCGGGGAGCTCTTCGACCGGATCATCGCCAAGGGACACTACACGGAGCGCGCCGCGGCTGCGTTGCTGCGCACCATTGTGGAGATCATCCATACCTGCCACTGCATGGGCGTCATCCACCGCGACCTCAAGCCCGAGAACTTCCTCCTGCTCAACAAGGACGAGAACGCGCCGCTCAAGGCCACCGATTTCGGCCTCTCCGTCTTCTTCAAAGAAGGGGAGGTGTTCAGGGACATCGTGGGCAGCGCGTACTACATCGCGCCGGAGGTGCTGAAGCGGAACTACGGGCCGGAGGCGGACATCTGGAGCGTCGGCGTCATGTTCTACATCCTGCTCTGCGGCGTCCCGCCCTTCTGGGCAGAGTCGGAGCACGGCATCTTCAACTCCATCCTGAGAGGGCAGGTGGACTTCGCCAGCGATCCGTGGTCGCGCATCTCCAGCGGCGCCAAGGACCTCGTCAGGAAGATGCTCACCTCCGACCCCAAGAAGAGGATTTCTGCCTACGACGTCCTCAATCACCCGTGGATAAAGGAAGACGGCGAGGCGCCTGACACGCCGCTGGACAACGCCGTACTCGGCAGGCTCAAGCAGTTCACGGCCATGAACCAGTTCAAGAAGGCGGCGCTGAGGGTGATCGCCGGGTGCTTGTCAGAGGAGGAGATCAAAGGGCTCAAGGAGATGTTCAAGGGAATGGACTCTGACAACAGCGGCACCATCACCGTCGACGAGCTCCGGAAAGGGCTGGCCAAGAAGGGGACCAAGCTCACCGAAGCTGAAGTCCAGCAGCTAATGGAAGCCGCGGACGCGGACGGGAACGGGACGATCGACTACGAGGAGTTCATCACGGCGACGATGCACATGAACAGGATGGACCGGGAGGAGCACCTCTACACGGCGTTCCAATACTTCGACAAGGACAACAGCGGGTATATCACGATAGAGGAGCTGGAGCAGGCCCTGAGGGAGAAGGGGTTGCTGGACGGCCGGGATATCAAGGACATCATATCGGAGGTGGACGGCGACAACGACGGAAGGATCAACTACACGGAGTTCGTGGCGATGATGAGGAAAGGGACCCCGGAGGCGAACCCAAAGAAGCGGCGCGACGTCGTGCTATAG |
|  | Protein | MGNCCPGSKDADPSDGGNPGEGSSNNAPSASGSASSAPAQNKPPAPIGPVLGRPMEDVKSIYNVGKELGRGQFGVTSLCTHKATGHKFACKTISKRKLSTKEDVEDVRREVQIMYHLAGQPGVVELKGAYEDKHSVHLVMELCAGGELFDRIIAKGHYTERAAAALLRTIVEIIHTCHCMGVIHRDLKPENFLLLNKDENAPLKATDFGLSVFFKEGEVFRDIVGSAYYIAPEVLKRNYGPEADIWSVGVMFYILLCGVPPFWAESEHGIFNSILRGQVDFASDPWSRISSGAKDLVRKMLTSDPKKRISAYDVLNHPWIKEDGEAPDTPLDNAVLGRLKQFTAMNQFKKAALRVIAGCLSEEEIKGLKEMFKGMDSDNSGTITVDELRKGLAKKGTKLTEAEVQQLMEAADADGNGTIDYEEFITATMHMNRMDREEHLYTAFQYFDKDNSGYITIEELEQALREKGLLDGRDIKDIISEVDGDNDGRINYTEFVAMMRKGTPEANPKKRRDVVL |
| BdCDPK12 | CDS | ATGTCCACAACTGAAAGCAGAAGGCTGTCTGATGACTATGAAGTGGTGGATGTCCTTGGCCGAGGTGGTTTCTCAATAGTGAGAAGAGGAGTGAGCAAGTCTGAAGGAAATATACAAGTTGCCATAAAGACTCTCCGAAGGCTTGGACCAGCGATGATGGGGATGCAACAAGGATCAAAGGGTGCGCCGAGCTCTGGGCTACCGGTGTGGAAGCAGGTATCCATCTCCGATGCTTTGCTAACTAATGAGATACTTGTTATGAGGAGGATAGTGGAGAATGTTGCGCCGCATCCAAATGTTATCAACCTGCATGATGTGTATGAAGATGTTCACGGTGTGCACCTTGTACTTGAGCTGTGCTCAGGTGGTGAACTGTTTGATAGGATAATAGGACGTGACAGGTACTCGGAGTTCGATGCAGCTGCTGTCATTAGTCAGATTGCTAGTGGATTGAAGGCTCTTCATAAGGCGAACATCATACACAGGGACTTGAAGCCAGAGAATTGCCTCTTCTTGGACAGAAAAGAGAATTCCACATTGAAGATCATGGATTTTGGTTTGAGTTCTGTAGAAGATTTCAGTGACCCAATTGTGGCGCTGTTTGGATCAGTAGATTATGTTTCACCAGAAGCACTCTCGAGGCAAGAGGTTTCAGCTGCAAGTGATATGTGGTCTGTTGGGGTGATTCTGTATATTCTTTTATCCGGATGCCCACCATTTCATGCTGCAACGAATCGAGAAAAACAGCAAAGGATCCTGCAAGGTGAATTCAGTTTTCAGGAGCACACATGGAAAACAATATCTTCATCGGCCAAAGATTTGATATCCAGTCTCCTTTCTGTTGAACCTTACAAAAGGCCCACTGCGAGTGATCTTCTGTTGCATCCGTGGGTGATAGGAGACTGCGCCAAGCAAGATCTCATGGATGCAGAGGTCGTCTCAAAACTGCAGAGGTTCAATGCTAGAAGGAAACTGCGAGCAGCAGCAATAGCCAGCGTCCTGAGCAGCAAAGTGGCATTGAGGACAAAAAGGCTGAGGAGTCTTTTGGGAACCCATGACCTTACCTCCGAGGAGCTAGATAACCTGCGGCTTCATTTTTCACGGATATGTGCAGACGGAGAGAACGCCACGCTGTCAGAATTCGAGCAGGTGCTGAAAGCAATGAAGATGGACTCGCTGGCCCCTCTGGCTCCCCGTGTATTTGATCTGTTCGACAACAACCGTGACGGGACTGTCGACATGAGGGAGATCCTCTGCGGGCTCTCCAGCCTGAGGAACTCGCGAGGCGACGATGCCCTGCGGCTTTGCTTCCAGATGTACGACACCGATCGGTCAGGCTGCATCAGCAAGGAGGAGCTGGCGTCGATGCTCCGGGCCCTGCCGGAGGAGTGCCTTCCGGGCGACATCGCGGAGCCGGGGAAGCTGGACGAGGTGTTCGACGAGATGGACGCCAACGGCGACGGCGAGGTCACCTTCGACGAGTTCAAGGCGGCGATGCAGAAGGACAGCTCCCTCCAGGACGTGCTCCTCTCCTCCCTGCGCCCCCCGGGCCCAGGACAGCAACAGCAGCAATAA |
|  | Protein | MSTTESRRLSDDYEVVDVLGRGGFSIVRRGVSKSEGNIQVAIKTLRRLGPAMMGMQQGSKGAPSSGLPVWKQVSISDALLTNEILVMRRIVENVAPHPNVINLHDVYEDVHGVHLVLELCSGGELFDRIIGRDRYSEFDAAAVISQIASGLKALHKANIIHRDLKPENCLFLDRKENSTLKIMDFGLSSVEDFSDPIVALFGSVDYVSPEALSRQEVSAASDMWSVGVILYILLSGCPPFHAATNREKQQRILQGEFSFQEHTWKTISSSAKDLISSLLSVEPYKRPTASDLLLHPWVIGDCAKQDLMDAEVVSKLQRFNARRKLRAAAIASVLSSKVALRTKRLRSLLGTHDLTSEELDNLRLHFSRICADGENATLSEFEQVLKAMKMDSLAPLAPRVFDLFDNNRDGTVDMREILCGLSSLRNSRGDDALRLCFQMYDTDRSGCISKEELASMLRALPEECLPGDIAEPGKLDEVFDEMDANGDGEVTFDEFKAAMQKDSSLQDVLLSSLRPPGPGQQQQQ |
| BdCDPK13 | CDS | ATGGGCAACTGCTGCCGCTCGCCGGCAGCGGCGGCGCGGGAGGACGTCAAGTCGTCGCACTTCCCCACCTCGACCGGGAAGAAGAAGCCCCACCAGGCGCGGAACGGCGGCGGCGCGGGGGGAGGCGGAGGCGGAGGCGGCGGCGGCGGCGGGGAGAAGAAGCTGCTCTCGGTGCTCGGGGAGGAGGGGCGCGACGTGAGCGGCGGGATCGACGAGAAGTACGCGCTGGACCGGGAGCTGGGCCGCGGCGAGTTCGGGGTGACGTACCTGTGCATGGATCGGGGCTCCAAGGAGCTGCTCGCGTGCAAGTCCATCTCGAAGCGGAAGCTGCGGACGCCGGTCGACGTGGAGGACGTGCGCCGGGAGGTGGCCATCATGCGGCATCTGCCCAAGAGCCCCAGCATCGTCACGCTCCGCGAGGCCTGCGAGGACGACGGCGCCGTGCACCTCGTCATGGAGCTCTGCGAGGGAGGGGAGCTGTTCGACCGAATCGTCGCGCGGGGCCACTACACGGAGCGCGCGGCGGCCGCGGTCACGCGCACCATCCTCGAGGTCGTGCAGCTCTGCCACCACCACGGCGTCATCCACCGCGACCTCAAGCCCGAGAACTTCCTCTTCGCCAACAAGAAGGAGAACTCCCCGCTCAAGGCCATCGACTTCGGTCTCTCCATCTTCTTCAAACCCGGCGAGAAATTTTCTGAAATTGTCGGAAGTCCCTACTATATGGCTCCTGAAGTGCTGAAGAGAAACTATGGACCTGAAATAGACATATGGAGTGCTGGCGTTATCTTGTATATCTTGTTATGTGGCGTTCCTCCATTTTGGGCTGAGACTGAACAAGGAGTGGCACAAGCTATCCTTCGTGGAAATATAGATTTTAAAAGAGAACCCTGGCCCCAAGTTTCTGATAATGCTAAAGATCTAGTTCGGCAGATGCTTCAGCCTGATCCGAAAATTAGGCTAACGGCAAAGCAAGTTCTTGAGCATACATGGCTTCAAAATGCTAAGAAAGCTCCAAATGTTCCTCTTGGAGACATTGTAAAGTCAAGACTGAAACAATTTTCAAGGATGAACAGATTCAAAAGAAGGGCTCTAAGGGTTATTGCTGACCACTTGTCGGCTGAAGAGGTTGAGGACATAAAAGACATGTTCAAGGTGATGGATACTGATAATGATGGTATAGTCTCTTATGAAGAGTTGAAGAGCGGGATCGCAAAATTTGGTTCTCACCTTGCAGAATCTGAAGTGCAGATGTTAATTGAAGCTGTGGATACAAATGGTAGAGGGGCACTAGATTATGGCGAATTTTTGGCTGTCTCGCTTCATTTACAAAGGATGGCAAATGATGAGCACCTTCGGCGGGCCTTCCTATTTTTTGACAAGGATGGCGATGGATTTATTGAGCCAGGGGAGCTTCAGGAGGCCCTGGTGGAGGATGGGACTGCTGATATCACAGAAGTGGTGAAGGACATATTGCAAGAAGTTGACACAGACAAGGATGGCAAAATTAGTTTTGAAGAATTCGTAGCGATGATGAAGACTGGCACAGACTGGAGAAAGGCATCCCGGCATTATTCGAGAGGTCGCTTCAATAGCCTTAGCATAAGGCTTATCAAGGATGGATCTGTAAAGCTGGGAAATGAGTGA |
|  | Protein | MGNCCRSPAAAAREDVKSSHFPTSTGKKKPHQARNGGGAGGGGGGGGGGGGEKKLLSVLGEEGRDVSGGIDEKYALDRELGRGEFGVTYLCMDRGSKELLACKSISKRKLRTPVDVEDVRREVAIMRHLPKSPSIVTLREACEDDGAVHLVMELCEGGELFDRIVARGHYTERAAAAVTRTILEVVQLCHHHGVIHRDLKPENFLFANKKENSPLKAIDFGLSIFFKPGEKFSEIVGSPYYMAPEVLKRNYGPEIDIWSAGVILYILLCGVPPFWAETEQGVAQAILRGNIDFKREPWPQVSDNAKDLVRQMLQPDPKIRLTAKQVLEHTWLQNAKKAPNVPLGDIVKSRLKQFSRMNRFKRRALRVIADHLSAEEVEDIKDMFKVMDTDNDGIVSYEELKSGIAKFGSHLAESEVQMLIEAVDTNGRGALDYGEFLAVSLHLQRMANDEHLRRAFLFFDKDGDGFIEPGELQEALVEDGTADITEVVKDILQEVDTDKDGKISFEEFVAMMKTGTDWRKASRHYSRGRFNSLSIRLIKDGSVKLGNE |
| BdCDPK14 | CDS | ATGGGCAACCGCACCTCGCGGCACCGCCGCGCCGCCGCCGCCGAGCAGCCGACGCCGGTTCCGTCCCCGCCGCCGGCCCAGCCCAAACCCGAGCCGCAACAGCAGACGCTCCACTGGCCGCAGCCGAAGCCCCAGCCGCCCCCCGCCCCGGCGGTGGAAGCGGGCGTGGTGGCGATGGGGCGCGTGCTGGGGCGGCCGATGGAGGACGTGCGCGCGACCTACACCTTCGGCCGCGAGCTCGGCCGGGGCCAGTTCGGGGTCACCTACCTCGTCACGCACAAGGCCACGGGCCAGCGCTTCGCCTGCAAGTCCATCGCCACGCGGAAGCTCGTCCACCGCGACGACATCGAGGACGTGCAGCGGGAGGTGCAGATCATGCACCACCTCACGGGCCACCGCAACATCGTCGAGCTCCGCGGCGCCTACGAGGACCGCCACTCGGTCAACCTCGTCATGGAGCTCTGCGAGGGCGGGGAGCTGTTCGACCGCATCATCGCGCGGGGCCACTACTCCGAGCGAGCCGCCGCCGTGCTCTGCCGCGAGATGGTCTCCGTCGTGCACAGCTGCCACTCGATGGGGGTTTTCCATCGGGATCTCAAGCCTGAGAACTTTCTGTTTCTGAATAACAAGGAAGACTCGCCACTCAAGGCCACTGACTTTGGCCTCTCCGTCTTCTTCAAGCATGGGGAGCAGTTTAAGGATCTTGTTGGAAGCGCATATTATGTTGCTCCTGAGGTGCTGAAACGGCACTATGGAGCAGAAGCTGACATATGGAGTGCAGGGGTTATTCTTTACATCCTTCTGTCTGGTGTTCCTCCTTTTTGGGCAGATAATGAGGATGGCATATTTAAGGCTGTTTTGCTTGGTCACATTGATTTCTCGTCTGACCCCTGGCCTTCAATTTCTAATCCCGCGAAAGATTTGGTCAAGAAGATGCTGCGACAAGACCCCAAAGAGCGCTTAACTGCTGCAGAGATTTTAGACCACCCATGGATTAAGGAAGATGGAGAGGCCCCGGATAAGCCACTTGACATTACAGTTATCAGTAGAATGAAGCAGTTCAGGGCGATGAACAAGCTTAAGAAAGTTGCACTGAAGATTGTTGCAGAGAACTTGTCTGAGGAAGAGATTACAGGCTTGAAAGAAATGTTCAGATCCCTGGATACTGATAACAGCGGGACAATTACTCTTGAAGAGCTACGATCTGGTTTACCAAAACTTGGCACCAAAATATCTGAATCAGAAATTACACAACTGATGGAGGCGGCTGATGTTGATGGAAATGGGACCATTGATTATTCTGAGTTTGTATCGGCGACAATGCACATGAATAGATTGGAGAAGGAAGACCACATACTTAAAGCATTTGAATACTTTGATAAGGACCACAGCGGATACATAACAGTAGATGAGTTGGAAGAAGCTCTGAAGAAATATGACATGGGGGACGATAAAACAATTAAAGAAATCATTGCTGAAGTAGATACAGATAATGACGGAAGAATCAACTACCAGGAGTTCGTTGCTATGATGAGAAACAACAGCCCTGAGATTGTTCCAAATCGGAGACGCATGTTTTAA |
|  | Protein | MGNRTSRHRRAAAAEQPTPVPSPPPAQPKPEPQQQTLHWPQPKPQPPPAPAVEAGVVAMGRVLGRPMEDVRATYTFGRELGRGQFGVTYLVTHKATGQRFACKSIATRKLVHRDDIEDVQREVQIMHHLTGHRNIVELRGAYEDRHSVNLVMELCEGGELFDRIIARGHYSERAAAVLCREMVSVVHSCHSMGVFHRDLKPENFLFLNNKEDSPLKATDFGLSVFFKHGEQFKDLVGSAYYVAPEVLKRHYGAEADIWSAGVILYILLSGVPPFWADNEDGIFKAVLLGHIDFSSDPWPSISNPAKDLVKKMLRQDPKERLTAAEILDHPWIKEDGEAPDKPLDITVISRMKQFRAMNKLKKVALKIVAENLSEEEITGLKEMFRSLDTDNSGTITLEELRSGLPKLGTKISESEITQLMEAADVDGNGTIDYSEFVSATMHMNRLEKEDHILKAFEYFDKDHSGYITVDELEEALKKYDMGDDKTIKEIIAEVDTDNDGRINYQEFVAMMRNNSPEIVPNRRRMF |
| BdCDPK15 | CDS | ATGGGCAACTGCTGCCCGGGCTCAGGGGATGCGGAGCCCGCCCCCTCCGCCTCCGCCGACCCTTCCTCCACCCGCCGCTCCGGCGCCTCCCCTACCTCGGCGCCGGCCCAGAACAAGCCCCCCGCCCCAATCGGCCCCGTCCTGGGCCGGCCCATGGAGGACGTCCGCAGCATCTACACCGTCGGCAAGGAGCTCGGGCGCGGCCAGTTCGGCGTCACCTCCCTGTGCACCCACAAGGCCACGGGCCAGAAGTTCGCGTGCAAGACCATCGCCAAGCGGAAGCTGTCCACCAAGGAGGACGTGGAGGACGTCCGCCGGGAGGTGCAGATCATGTACCACCTGGCCGGCCAGCCAAACATCGTGGAGCTCAAGGGCGCCTACGAGGACAAGCAGTCCGTGCACCTCGTCATGGAGCTCTGCGCCGGCGGGGAGCTCTTCGACCGGATCATCGCCAAGGGCAAGTACACGGAGCGCGCCGCGGCCTCGCTGCTGCGCACCATCGTCGAGATCGTCCACACCTGCCACTCCCTCGGCGTCATCCATCGTGATCTCAAGCCCGAGAACTTCCTCCTCAGCAGCAAGGAGGAGGACGCGCCGCTCAAGGCCACCGACTTCGGGCTCTCCGTCTTCTTCAAGCAAGGGGAGGTGTTCAAGGACATCGTGGGCAGCGCCTACTACATCGCGCCGGAGGTCCTCAAGCGGAACTACGGGCCGGAGGCGGACATCTGGAGCGTCGGCGTCATCCTCTACATCCTTCTCTGCGGTGTTCCTCCCTTCTGGGCCGAATCGGAGCACGGCACCTTCAACTCCATCCTGCGGGGGCAGGTGGACTTCACCAGCGACCCGTGGCCGCGCATTTCGCCCGGAGCCAAGGACCTCGTCAGGAAGATGCTCACCTCTGACCCCAACAAGAGGATCTCTGCCGACGATGTCCTCAATCATCCTTGGATCAAGGAAGACGGAGAAGCGCCGGATACACCACTGGACAACGCCGTCATGGGCAGGCTCAAGCAGTTCAGGGCTATGAACCAGTTCAAGAAAGCCGCGCTAAGGGTCATCGCCGGATGCCTGTCGGAGGAAGAGATCAGAGGGCTCAAGGAGATGTTCAAGAGCATGGACTCGGACAACAGCGGCACCATCACCGTCGACGAGCTCCGGAAAGGCCTTGGCGGCAAGCAGGGTACCAAGCTCACCGAGGCCGAAGTGGAGCAGCTAATGGAAGCCGCCGATGCAGATGGCAACGGGACGATCGACTACGAGGAGTTCATCACGGCGACGATGCACATGAACAGGATGGACAGGGAGGAGCATCTCTACACCGCGTTCCAGTACTTCGACAAGGACAACAGCGGCTGCATCTCCAAGGAGGAACTGGAGCAGGCCCTGCGGGAGAAAGGACTCCTGGACGGCAGAGACATCAAAGACATCATATCGGAAGTCGACGCCGACAACGACGGGAGGATCGACTACAGCGAGTTCGTGGCGATGATGAAGAAGGGGAACCCCGAGGCGAACCCCAAGAAGCGGCGCGAAATCGTTCTGTAG |
|  | Protein | MGNCCPGSGDAEPAPSASADPSSTRRSGASPTSAPAQNKPPAPIGPVLGRPMEDVRSIYTVGKELGRGQFGVTSLCTHKATGQKFACKTIAKRKLSTKEDVEDVRREVQIMYHLAGQPNIVELKGAYEDKQSVHLVMELCAGGELFDRIIAKGKYTERAAASLLRTIVEIVHTCHSLGVIHRDLKPENFLLSSKEEDAPLKATDFGLSVFFKQGEVFKDIVGSAYYIAPEVLKRNYGPEADIWSVGVILYILLCGVPPFWAESEHGTFNSILRGQVDFTSDPWPRISPGAKDLVRKMLTSDPNKRISADDVLNHPWIKEDGEAPDTPLDNAVMGRLKQFRAMNQFKKAALRVIAGCLSEEEIRGLKEMFKSMDSDNSGTITVDELRKGLGGKQGTKLTEAEVEQLMEAADADGNGTIDYEEFITATMHMNRMDREEHLYTAFQYFDKDNSGCISKEELEQALREKGLLDGRDIKDIISEVDADNDGRIDYSEFVAMMKKGNPEANPKKRREIVL |
| BdCDPK16 | CDS | ATGGGCAACTGCTGCCGCTCGCCGGCCGCCGCCGCGCGGGAGGACGTCAAGTCCTCGCACTTCCCCGCGGCCGCCGCGAAGAAGAAGCCCCACCAGCCGCGGACCGGCGCCGGCGGCGGGCAGAAGCGGCTGGCCGTGCTGGGCGAGGACGGGTGCGAGTTCATCGGCGGGATCGACGACAAGTACGTGCTTGACAGGGAGCTCGGGCGCGGGGAGTTCGGGGTGACGTACCTGTGCATGGATCGGGACACCAAGGAGCTGCTCGCCTGCAAGTCCATCTCCAAGCGGAAGCTGCGGACGCCGGTCGACGTCGAGGACGTCCGCCGGGAGGTGGCCATCATGCGCCACCTGCCCAAGAGCCACAGCATCGTGTCGCTGCGGGAGGCGTGCGAGGACGAGGGCGCCGTGCACCTCGTCATGGAGCTCTGCGAGGGCGGCGAGCTCTTCGACCGCATCGTCGCCCGGGGCCACTACACGGAGCGGGCCGCCGCCAACGTCACCCGCACCATCGTCGAGGTCGTGCAACTCTGCCACCGCCACGGTGTCATCCACCGGGACCTCAAGCCCGAGAACTTCCTTTTCGCCAATAAGAAAGAGAACTCGCCACTCAAGGCCATCGATTTCGGCCTCTCGATTTTCTTCAAGCCTGGTGAAAAGTTTTCAGAAATTGTCGGAAGCCCTTATTACATGGCTCCTGAAGTATTGAAGAGAAACTATGGACCAGAAATAGACATCTGGAGCGCCGGGGTTATCTTGTATATTTTACTATGTGGAGTTCCTCCATTTTGGGCAGAGACTGAGCAAGGGGTGGCACAAGCTATCCTTCGTGGAAATATTGATTTTAAGCGGGAACCCTGGCCCAACGTTTCAGAAAATGCTAAAGATTTAGTTCGACGTATGTTGGAACCTGATCCAAAGATCAGGTTAACTGCAAAGCAGGTTCTTGAGCACCATTGGCTTCAAAACGCTAAGAAAGCTCCAAATGTTCCTCTTGGAGATATTGTGAAGTCAAGGCTCAAACAATTTTCTAGGATGAATCGATTCAAAAGAAGAGCTTTAAGGGTCATTGCTGATCATTTGTCTGCCGAGGAAGTTGAGGACATTAAGGAGATGTTCAAGGCAATGGATACTGATAATGATGGTATTGTTTCTTGTGAAGAACTGAAAAGCGGAATAGCAAAATTCGGCTCCCATCTTGCTGAATCAGAAGTGCAGATGCTTATCGAAGCTGTGGATACAAATGGCAAGGGAGCATTAGATTATGCCGAATTTCTAGCGGTCTCTCTTCATTTGCAAAGGATGGCAAATGACGAGCACCTTCGGCGGGCCTTCCTATTCTTCGACAAGGATGGCAATGGTTACATTGAGCCAGATGAGCTTCGAGAGGCTTTAAAGGATGATGGAGCTGCTGATAGCATGGAAGTGGTGAATGACATATTGCAAGAAGTAGACACTGATAAGGTGACGATTCTCTTGTATGAATGTATCCACTACAACCATTACTTTGGTTCTCACTGTCTAAAATGTCATCTGCAGGATGGCAAGATTAGCTACGACGAATTTGTAGCAATGATGAAGACCGGCACAGATTGGAGAAAGGCATCACGGCATTACTCCAGAGGAAGATTCAACAGCCTTAGCATGAAGCTTGTGAAGGATGGGTCGGTAAAACTGGGCATCGAGTGA |
|  | Protein | MGNCCRSPAAAAREDVKSSHFPAAAAKKKPHQPRTGAGGGQKRLAVLGEDGCEFIGGIDDKYVLDRELGRGEFGVTYLCMDRDTKELLACKSISKRKLRTPVDVEDVRREVAIMRHLPKSHSIVSLREACEDEGAVHLVMELCEGGELFDRIVARGHYTERAAANVTRTIVEVVQLCHRHGVIHRDLKPENFLFANKKENSPLKAIDFGLSIFFKPGEKFSEIVGSPYYMAPEVLKRNYGPEIDIWSAGVILYILLCGVPPFWAETEQGVAQAILRGNIDFKREPWPNVSENAKDLVRRMLEPDPKIRLTAKQVLEHHWLQNAKKAPNVPLGDIVKSRLKQFSRMNRFKRRALRVIADHLSAEEVEDIKEMFKAMDTDNDGIVSCEELKSGIAKFGSHLAESEVQMLIEAVDTNGKGALDYAEFLAVSLHLQRMANDEHLRRAFLFFDKDGNGYIEPDELREALKDDGAADSMEVVNDILQEVDTDKVTILLYECIHYNHYFGSHCLKCHLQDGKISYDEFVAMMKTGTDWRKASRHYSRGRFNSLSMKLVKDGSVKLGIE |
| BdCDPK17 | CDS | ATGGGCGGCTGCTTCTCCACCAACACACCCGCGACGCCGGCGGCCGAGCGAAGGCGGCGGCGGCGGCGGAGGCAGAGGGCTGCGTCGCCGGAGAAGGGCAGCGGCGGCGGGGCGGAGGGGGTCGCCCGCGTGGTGGAGTTCGGCTACGAGCGGGACTTCGAGGGCCGGTACGAGGTCGGCCGGCTGCTGGGGCACGGCCAGTTCGGCTACACCTTCGCCGCCACCGACCGCGGCTCCGGGGACCGCGTCGCCGTCAAGCGCATCGACAAGGCCAAGATGAACCGACCTGTTGCTGTGGAAGATGTAAAGAGAGAAGTGAAGATTCTTAAAGCCCTTAAAGGCCATGAGAACATTGTTCATTTCTACAATGCATTTGAGGACGATTCATATGTGTATATTGTGATGGAACTATGCGAGGGTGGTGAACTATTGGACCGGATCTTGGCAAAGAAAAACAGCCGCTATAGCGAGAAAGATGCAGCAGTGGTAGTGCGGCAAATGCTCAAAGTGGCTGCTGAGTGTCATCTGCGTGGGTTAGTCCACCGAGATATGAAGCCTGAGAACTTCCTTTTCAAATCAACCAAGGAGGACTCGCCTCTGAAGGCCACAGATTTTGGTTTATCAGACTTTATAAATCCAGGGAAGAAGTTCCGTGATATAGTCGGCAGTGCCTATTATGTTGCACCAGAAGTATTAAAACGGCGGTCTGGCCCTGAGTCTGATGTTTGGAGCATAGGGGTCATAACTTACATTTTGCTCTGTGGGAGACGTCCTTTTTGGGATAAAACAGAGGATGGTATATTCAAGGAGGTTCTAAGAAACAAGCCTGATTTTCGTAAGAGGCCTTGGCCAAGCATCAGTACAAGTGCTAAAGATTTTGTTAAAAGGTTGCTTGTGAAGAACCCAAGGGCGAGATTAACAGCAGCTCAAGCCCTCTCACATCCATGGGTGAGAGAAGGAGGAGATGCATCTGAGATCCCTGTTGATATATCTGTATTGTACAACATGCGCCAGTTTGTCAAATACAGCCGTTTTAAGCAATTTGCGCTGAGGGCTTTAGCAAGTACAGTCAACGAGGAAGAACTAGCAGATCTGAAGGATCAATTCGATGCAATTGATATTGATAAAAGTGGATCGATTAGTATTGAGGAAATGCGGCATGCCCTTGCAAAGGATCTTCCTTGGAGATTGAAGGGTCCCCGTGTTCTCGAGATTATTCAAGCAATCGACAGCAACACCGATGGTCTTGTCGACTTCAAAGAGTTTGTTGCAGCAACTCTCCATATACACCAGATGGCCGAGCTGGACTCTGAAAGGTGGGGCCTACGCTGCCAAGCTGCCTTCAGCAAATTTGATCTGGATGGCGATGGATACATCACACCTGACGAGCTTAGAATGGTGCAGCACACCGGCTTAAAGGGGTCCATCGAGCCATTGCTGGAGGAGGCCGACATCGACAAGGACGGGAGGATAAGCTTGTCCGAATTCCGCAAGCTTCTGCGAACCGCGAGCATGAGCAACCTCCCGAGCCCGACCGGAGTCCCGAATCCCCAAGCTTTGTGA |
|  | Protein | MGGCFSTNTPATPAAERRRRRRRRQRAASPEKGSGGGAEGVARVVEFGYERDFEGRYEVGRLLGHGQFGYTFAATDRGSGDRVAVKRIDKAKMNRPVAVEDVKREVKILKALKGHENIVHFYNAFEDDSYVYIVMELCEGGELLDRILAKKNSRYSEKDAAVVVRQMLKVAAECHLRGLVHRDMKPENFLFKSTKEDSPLKATDFGLSDFINPGKKFRDIVGSAYYVAPEVLKRRSGPESDVWSIGVITYILLCGRRPFWDKTEDGIFKEVLRNKPDFRKRPWPSISTSAKDFVKRLLVKNPRARLTAAQALSHPWVREGGDASEIPVDISVLYNMRQFVKYSRFKQFALRALASTVNEEELADLKDQFDAIDIDKSGSISIEEMRHALAKDLPWRLKGPRVLEIIQAIDSNTDGLVDFKEFVAATLHIHQMAELDSERWGLRCQAAFSKFDLDGDGYITPDELRMVQHTGLKGSIEPLLEEADIDKDGRISLSEFRKLLRTASMSNLPSPTGVPNPQAL |
| BdCDPK18 | CDS | ATGGGTAATTCGTGCCCAAATGGAACCCGTGGGAAGAAATTCATTGACTACAACCGGTTCGAGAACGAGCGTTTGGCTTCTAGGTTTGATGATGTGAACGATACAGAGGATTGCTTTGCAGGACTTATGCAAAAAGTGTTGAGCCTCAGGTCCACCCGTGTCCTCGAACGAGAAACTCCAAACATAAGGGAACATTACACCCTTGGCCATAAGCTTGGGCAGGGTAAGTTTGGTACGACGTACCTCTGCACCGAGATCACCACCAGGTGCCAGTACGCGTGCAAGTCCATCTTGAAGGCCAAGTTCTGCCACACGGAAGATATCGAGGATGTGCGACGTGAAATCCAGATAATGCACCATCTCTCGGGCCAGAAGAACATAGTCGCAATCAAGGATGAGTATGAGGACAAAGAGGCCGTGCACATCGTCATGGAGCTTTGTGCAGGCGGAGAACTATTTGACCGCATTCATCAAAAAGGACATTATAGTGAGCATAAGGCTGCTAAGCTGACAAGAGTTATCGCTGGTGCCATAGCGAAGTGCCATTCACTTGGGGTGATGCATCGGGATCTCAAGCCAGAAAACTTCCTCTTGGTGGATAAAGATAATGATTTGTCTATAAAAGCAATCGATTTTGGTCTGTCTGTGTTCTTCGAACCAGGCCAGATTTTCACTGATTTAGTGGGAAGCCCATTTTACGTTGCTCCAGAGGTATTGGGTCAGCATTATGGACCTGAGGCTGATGTATGGGCAGTTGGAGTGATACTCTATATATTGCTAAGTGGCGACCCGCCATTTTTGGGAGATACGCAAGACAAAATATTTGCTAAAATTCGGGAAGGACGTGCTGATTTGGAATCAAAACTGTGGCCCACTATATCCGACAGTGCAAAGGATCTTGTAAGAAAAATGCTCTGTCCTTCTCCAGCAGAGCGTTTGAAGGCCCATGAAGTGTTAGAGCATCCCTGGATATGTGATATTGGAGTGGCTACTGAACAAGCTATGGACCCAAGTGTTCTTTCTCGTCTCAATCAATTCTATTCAATGAATAGGTTAAAGAAATTGGCTCTGCAAGTTATTGCTGAGCGTCTTTCAGAGGACGAGCTTGCTGGGTTAAGGGAAACGTTCAAGGCAATGGACACTGAAAATACAGGATTGGTTACTCTTGGTGGAATTACTGATTCGATGGAAGCGGCTGATAATGACGCCACAAAGACTGTCAACTCGGAGGATTTTATTGCTGCATCAGTACCTCTTACTAAATTGGAACACGATGAACACCTGATGGCAGCCTTTACATATTTTGACAAAGATGGAAGTGGTTATATCACGGTCGACAAGCTTCAGAAAGCTTGCGTCGAACGCAATGTGGAAGATAAATTCCTCGAAGAGACAATACTGGAGGTTGATCAAAACAATGATGGTAAAATCGATTACGCCGAGTTTGTAGTGATGATGCAGAGCAACAACTTAGGGATGGAGGGCAGCCTGAATGTAGCCATGAGGCAGACACCGCGAGTATACTGA |
|  | Protein | MGNSCPNGTRGKKFIDYNRFENERLASRFDDVNDTEDCFAGLMQKVLSLRSTRVLERETPNIREHYTLGHKLGQGKFGTTYLCTEITTRCQYACKSILKAKFCHTEDIEDVRREIQIMHHLSGQKNIVAIKDEYEDKEAVHIVMELCAGGELFDRIHQKGHYSEHKAAKLTRVIAGAIAKCHSLGVMHRDLKPENFLLVDKDNDLSIKAIDFGLSVFFEPGQIFTDLVGSPFYVAPEVLGQHYGPEADVWAVGVILYILLSGDPPFLGDTQDKIFAKIREGRADLESKLWPTISDSAKDLVRKMLCPSPAERLKAHEVLEHPWICDIGVATEQAMDPSVLSRLNQFYSMNRLKKLALQVIAERLSEDELAGLRETFKAMDTENTGLVTLGGITDSMEAADNDATKTVNSEDFIAASVPLTKLEHDEHLMAAFTYFDKDGSGYITVDKLQKACVERNVEDKFLEETILEVDQNNDGKIDYAEFVVMMQSNNLGMEGSLNVAMRQTPRVY |
| BdCDPK19 | CDS | ATGGGGGGCTGCTACTCCGCCTACGCCTGCTCGCGCAAGCTGCGCGGCCGCCTCGGCAACAGCCTCTCCTTCGTCCTCCCCGTCTCCGACCCCAACAACAACAAATCCGATGACGACGCCAACAACAACAACAAGGAGCGGTCGCCGAAGCCCAACAATGGCGCCGCCCAGTCCCCGTGGGAGGAGGAGGAGCCGATCCTGAGGACGACGGCGGCGGAGTTCGCGCGGCGGTACGTGCTGGGGAAGGAGCTAGGGCGGGGCGAGTTCGGGGTCACACGCCGCTGCAAGGACGCCGCCACCGGGGAATCCCTCGCCTGCAAGACCATCCGCCGCCACCGCCGCCGCCGCCGCAACAAGTCCGCTGCTGGTGGAGGTGGCGGCGGCGCGGCTGCTGCGGCTGCCGCTGCGGCCGCGGCGGCCGCGGCGCACCAGGCCGACGTGCGGCGCGAGGTGGCCATCATGCGGCGCATGTCTTCCTCGTCTTCCCCGGCGTCGGCGTCGTCCGTGGTCCGCCTCCGCGACGCCCGCGAGGACCCCGCCGACGGCTCCGTCCACCTCCTCATGGACCTCTGCGAGGGCGGCGAGCTCTTCGACCGCATCGTCGCCCGCGGCCACTACTCCGAGCGCGCCGCCGCCAAGCTCTTCCGCACCGTCGTCCACGTCGTCCAGCTGTGCCACGCGAACGGGGTGATGCACAGGGACCTAAAGCCGGAGAACTTTCTGTTCGCGGACAAGTCGGAGGACTCGCCGCTAAAGGTGATCGACTTCGGCCTCTCCGTCGTCTTCCGCCCCGGCGACCGCTTCACGGAGGTGGTGGGCAGCGCCTACTACATGGCCCCCGAGGTGCTCCGCCGCAGCTACGGGCCCGAGGCGGACGTCTGGAGCGCCGGCGTCATCCTCTACATCCTCCTCTGCGGCGTTCCCCCCTTCTGGGGCGACAACGACGAGAAGATCGCCCAGGCCGTCCTCCGCGGCGGGCTCGACTTCTCCCGGGAGCCATGGCCGCGCGTCTCCCCCAGCGCCAAGGACCTCGTGCGCCGCATGCTCGATCCCGATCCCTCCTCCCGCCTCACCGCCCCCCAAGTCCTAGAGCACCCGTGGCTGAAGAACGCGGAGACGGCGCCGAACGTGTCCCTCGGCTCAGCGGTGCGAGCCCGGCTCCAACAATTCTCCGCCATGAACAAGCTAAAGAAGCGAGCGCTCGGCGTCGTGGCGCGGAGCATGCCGGTGGAGGAGCTAGACAAGTACGTGCAAATGTTCCACATAATGGACAAGGACAAGAACGGCAACCTCTCCCTCGAGGAGCTCATGGAAGGGCTCCACATCAACGGGCAACCGGTCCCGGAGCCCGAGATCCGGATGCTCCTCGAGGCGGCCGACACCGACGGCAACGGGACCCTGGACTGCGACGAGTTCGTGACCGTCTCGCTCCACCTCAAGAAGATGACCAACGACGAGTACCTGGCGGCGGCGTTCCGGTACTTCGATAAAGACGGCAGCGGGTTCATCGAGGTGGAGGAGCTCCGGGAGGAGCTGGGGCCGAACGAGCAGGCGATCTTGGAGATTATCAGGGATGTTGATACTGATCAGGATGGGAGGATCAGTTACCAGGAGTTTGAGCTTATGATGAAGTCTGGGACGGATTGGAGGAATGGATCCAGGCATTATTCGAGAGCCAATTTTAGTAGCCTCAGCCGGAAGCTCTGCAAAGACGAAGGACCCGGGAGCTCTTCTTCTTGA |
|  | Protein | MGGCYSAYACSRKLRGRLGNSLSFVLPVSDPNNNKSDDDANNNNKERSPKPNNGAAQSPWEEEEPILRTTAAEFARRYVLGKELGRGEFGVTRRCKDAATGESLACKTIRRHRRRRRNKSAAGGGGGGAAAAAAAAAAAAAAHQADVRREVAIMRRMSSSSSPASASSVVRLRDAREDPADGSVHLLMDLCEGGELFDRIVARGHYSERAAAKLFRTVVHVVQLCHANGVMHRDLKPENFLFADKSEDSPLKVIDFGLSVVFRPGDRFTEVVGSAYYMAPEVLRRSYGPEADVWSAGVILYILLCGVPPFWGDNDEKIAQAVLRGGLDFSREPWPRVSPSAKDLVRRMLDPDPSSRLTAPQVLEHPWLKNAETAPNVSLGSAVRARLQQFSAMNKLKKRALGVVARSMPVEELDKYVQMFHIMDKDKNGNLSLEELMEGLHINGQPVPEPEIRMLLEAADTDGNGTLDCDEFVTVSLHLKKMTNDEYLAAAFRYFDKDGSGFIEVEELREELGPNEQAILEIIRDVDTDQDGRISYQEFELMMKSGTDWRNGSRHYSRANFSSLSRKLCKDEGPGSSSS |
| BdCDPK20 | CDS | ATGGGCAACACGTGCGGCGTCACCTTTAGATCCATGTACTTCTCCAGCTTCCGCGGCGCCTCGCAGCGCCACGACCCGGAGTACGCGCCCATCGCCGCCGCCGCCGCCGATGACCCGCCTGGCAAGCGGCCGTCGCGTCCGGCAGCTGCGGGACCTGACGGTTCCCTGGCGGCGGCCGCGGCGGACGATGCGCCGCCGCCTCCCGCCTCCGCCATGCGCAGGGGCGCGCTCGCCCCCGCGGAGCTGACGGCCAACGTGCTCGGCCACCCCACCCCGAGCCTCCACGACCACTACCTGCTCGGCCGAAAGCTCGGGCAGGGGCAGTTCGGCACCACCTACCTCTGCACCCACCGAGCCACGGGGGTGGATTACGCCTGCAAGTCGATCGGCAAGCGCAAGCTCATCACCAAGGAGGACGTCGAGGATGTGCGCCGCGAGATCCAGATCATGCACCACCTCGCCGGCCACCGGAACGTCGTCGCCATCAAGGGCGCCTACGAGGACCAGGCCTACGTCCACATCGTCATGGAGCTCTGCGCGGGCGGCGAGCTGTTCGACCGCATCATAAAGCGGGGGCATTACAGCGAGCGCAAGGCGGCCGAGCTCACGCGCATCGTTGTAGGGGTTGTCGAGGCGTGCCACTCGCTTGGGGTCATGCACAGGGACCTCAAACCTGAAAACTTCTTGCTGGCGAACAAGGATGACGACATGTCGCTTAAGGCCATCGATTTCGGCCTCTCTGTGTTCTTCAAGCCCGGTCAAGTGTTCACAGATGTTGTTGGAAGTCCTTACTACGTAGCTCCAGAAGTGCTGCGCAAATCTTATGGGCCAGAAGCTGATGTATGGACAGCTGGTGTAATTCTTTACATACTACTAAGTGGTGTACCACCATTTTGGGCAGAGACACAGCAAGGAATATTTGACGCAGTATTAAAAGGCACTATTGATTTTGACTGCGATCCCTGGCCTGTTATTTCTGAAAGTGCGAAGGATCTTATAAGAAGAATGTTGAATCCTCATCCTGCAGAACGTCTAACTGCTCATGAAGTTCTATGCCATCCTTGGATTTGTGATCAGGGAGTTGCTCCTGATCGACCACTTGATCCTGCTGTCCTTTCGCGCATTAAGCAGTTCTCAGCAATGAATAAGTTGAAGAAGATGGCTTTGCGAGTAATTGCTGAGAGCCTTTCAGAAGAGGAGATTGCGGGATTAAAAGAAATGTTTGAGGCAATGGACACAGATAACAGCGGTGCAATTACATATGATGAGCTGAAAGAGGGCATGAGAAAGTATGGTTCAACATTAAAAGATACTGAGATTCGTGATCTTATGGAAGCAGCAGATGTGGACAACAGTGGAACCATTGATTACATAGAATTCATTGCTGCTACGCTGCATCTCAATAAACTAGAACGAGAGGAACATCTAGTGGCAGCCTTTTCTTATTTTGACAAGGATGGCAGTGGTTACATTACAGTGGATGAGCTCCAGCAAGCTTGCAAAGAGCATAACATGCCAGATGCTTTTCTTGATGATGTCATAATAGAAGCTGATCAGGATAATGATGGTCGAATTGATTATGGGGAGTTCGTCGCCATGATGACAAAGGGCAATATGGGGGTTGGAAGAAGAACAATGAGAAACAGCCTGAATATTAGCATGACTGCATGA |
|  | Protein | MGNTCGVTFRSMYFSSFRGASQRHDPEYAPIAAAAADDPPGKRPSRPAAAGPDGSLAAAAADDAPPPPASAMRRGALAPAELTANVLGHPTPSLHDHYLLGRKLGQGQFGTTYLCTHRATGVDYACKSIGKRKLITKEDVEDVRREIQIMHHLAGHRNVVAIKGAYEDQAYVHIVMELCAGGELFDRIIKRGHYSERKAAELTRIVVGVVEACHSLGVMHRDLKPENFLLANKDDDMSLKAIDFGLSVFFKPGQVFTDVVGSPYYVAPEVLRKSYGPEADVWTAGVILYILLSGVPPFWAETQQGIFDAVLKGTIDFDCDPWPVISESAKDLIRRMLNPHPAERLTAHEVLCHPWICDQGVAPDRPLDPAVLSRIKQFSAMNKLKKMALRVIAESLSEEEIAGLKEMFEAMDTDNSGAITYDELKEGMRKYGSTLKDTEIRDLMEAADVDNSGTIDYIEFIAATLHLNKLEREEHLVAAFSYFDKDGSGYITVDELQQACKEHNMPDAFLDDVIIEADQDNDGRIDYGEFVAMMTKGNMGVGRRTMRNSLNISMTA |
| BdCDPK21 | CDS | ATGGGCAACTACTCGTGCCGCGATTCCAACAGCAGCACCGCCGACTCGGACCCCTTCCATCTCCACCTCCAAGCCCCCTGCACCACCAACACCATGCGCGGCCACCAGCACCACCTCTCCTCCCCGACCGCCGTCCTCGGCCACGACACCCCGCCGCTCACCTCCCTCTACACGCTCGGCCGCAAGCTCGGCCAGGGCCAGTTCGGCACCACCTACCTCTGCACCGACATCGCCACGGGGACACCCCTCGCCTGCAAGTCCATCGCCAAGCGCAAGCTCCTCACCCCCGAGGACGTGGACGACGTCCGCCGCGAGATCCAGATCATGCACCACCTGGCGGGCCACGCCAGCGTCGTCACCATCCGCGGCGCCTACGAGGACCCGCTCTACGTCCACATCGTCATGGACCTCTGCGAGGGCGGCGAGCTCTTCGACCGCATCGTCGCCCGCGGATACTTCTCCGAGCGGAAAGCCGCCGAGATCGCACGCGTCGTCGTCGGCGTCGTCGAGGCGTGCCATTCTCTGGGGGTCATGCACCGCGACCTCAAGCCCGAGAACTTCCTCTTGCTCGGCGGCGCCGGCGCCGGCAACGGAGAAGACGAAGACGACTCGGTCGCCGATCTCAAGGCCATCGATTTCGGACTCTCCGTCTTCTTCAAGCCCGGCCAGATCTTCACCGACGTCGTCGGAAGCCCGTACTACGTGGCCCCCGAGGTCCTCTGCAAGCACTACGGGCCCGAAGCCGACGTCTGGACGGCGGGGGTCATCGTCTACATCCTGCTCTCCGGCGTCCCGCCCTTCTGGGCCGAGACGCAGCAGGGGATCTTCGACGCGGTCCTAAAAGGCGCCATAGACTTCGACTCCGACCCGTGGCCCAACATCTCCCCCAGCGCCAAGGACCTCATCAAGAACATGCTCAAATCGCCCCCTGCCGAGCGGCTCACGGCGCACCAGGTGCTCTGCCACCCGTGGATCTGCGAGAACGGGGTGGCGCCCGACAAGCCGCTCGACCCGGCCGTCCTCTCCCGGCTCAAGCAGTTCTCCGCCATGAACCGCCTCAAGAAGATGGCGCTCCGCGTCATCGCGCGCAACCTCTCCGAAGAAGAGCTCGCCGGGCTCAAGGAGATGTTCAAAGCCATGGACACCGACGGCAGCGGCGCGATTACCTTTGAGGAGCTCAAGGAAGGGCTGAGGAGGCATGGGTCCAACTTGAGGGAGTCGGAGATCCGGGAGCTGATGCACGCCGCCGACGTCGACAACAGCGGCACCATCGACTACGACGAGTTCATCGCCGCCACCGTCCACATGAACAAGCTCGAGCGCGAGGAGCACTTGCTCGCCGCCTTTGCGTATTTCGATAAGGATGGCAGCGGGTACATCACCGTCGACGAGCTCGAGGAGGCATGCCGGGAGCATAACATGGCCGACGTCGGCATCGACGACATCATCCGGGAGGTCGACCAGGACAACGATGGTCGGATCGATTACGGGGAGTTCGTGGCGATGATGAAGAAGGGGATCATAGGGAACGGACGGCTCACCATGAGGCACACCTCCGACGGCAGCATCCTCTACCATGGAGCAGGAGAACTCACCTAG |
|  | Protein | MGNYSCRDSNSSTADSDPFHLHLQAPCTTNTMRGHQHHLSSPTAVLGHDTPPLTSLYTLGRKLGQGQFGTTYLCTDIATGTPLACKSIAKRKLLTPEDVDDVRREIQIMHHLAGHASVVTIRGAYEDPLYVHIVMDLCEGGELFDRIVARGYFSERKAAEIARVVVGVVEACHSLGVMHRDLKPENFLLLGGAGAGNGEDEDDSVADLKAIDFGLSVFFKPGQIFTDVVGSPYYVAPEVLCKHYGPEADVWTAGVIVYILLSGVPPFWAETQQGIFDAVLKGAIDFDSDPWPNISPSAKDLIKNMLKSPPAERLTAHQVLCHPWICENGVAPDKPLDPAVLSRLKQFSAMNRLKKMALRVIARNLSEEELAGLKEMFKAMDTDGSGAITFEELKEGLRRHGSNLRESEIRELMHAADVDNSGTIDYDEFIAATVHMNKLEREEHLLAAFAYFDKDGSGYITVDELEEACREHNMADVGIDDIIREVDQDNDGRIDYGEFVAMMKKGIIGNGRLTMRHTSDGSILYHGAGELT |
| BdCDPK22 | CDS | ATGGGCAACGTCTGCTGCGTCGGTCCGCGCCGCAGCTTCGCCAAGACCCGCTTCTTCAGCGCCCTCTCCAGCCCCATCTGGCGCCGCTCCGGCGCCCCCGCCGGCTCCTCCTCCCCTTCCCGCCCCACCGTCACCACCCGCTCCGTCCCCGTCGTCCAGCCCACCGCCTCCACCCCAACTCCTCCCCCACGCGCTTCCGCCGTCATCAAGCCCCCGCCCGAGCCCACCCACACGGCCCCTCCTGCCCCCATCGTCATCTCCGAGCCCTCCAGACCCCAACCTCCAGAACCACAGCCCGTCGTCCAAAATGATCCCCCTCACCACCACCACCACCAACAACAAGAAGAGCAGCAGCAACAACAACAAGCCCAGTCGCGGCCGAAGAAGCCAACGCACATCAAGCGGGTGTCCAGCGCGGGGCTGCAGGTGGCGTCCGTGCTGCGGCGGAAGACGGAGAACCTCAAGGACAAGTACAGCCTGGGCCGGAAGCTGGGCCAGGGCCAGTTCGGCACGACGTACCTGTGCGTGGACAAGGCGTCCGGGCAGGAGCTGGCCTGCAAGTCCATCGCCAAGCGGAAGCTCATCACGGACGAGGACGTGGAGGACGTGCGGCGGGAGATCCAGATCATGCACCACCTGGCGGGACACCCCAGCATCATCTCCATCCGGGGCGCATACGAGGACGCCGTCGCCGTGCACGTCGTCATGGAGCTCTGCGCCGGCGGGGAGCTCTTCGACCGGATCGTGCGCCGGGGGCATTACACGGAGAGGCAGGCCGCCGAGCTCGCGCGCGTCATCGTCGCCGTCGTCGAGTCGTGCCACTCGCTCGGGGTCATGCACCGGGACCTCAAGCCTGAGAACTTCCTCTTTGTGGGTAATGAGGAGGACGCCGCGCTCAAGACCATTGACTTTGGGTTATCCATGTTCTTCCGGCCCGGGGAGATGTTTACCGACGTCGTGGGGAGCCCGTATTACGTCGCGCCCGAGGTGCTCAAGAAGAACTATGGCCAGGAAGCTGATGTGTGGAGCGCCGGTGTCATCGTCTACATCCTGCTCTGCGGCGTGCCTCCCTTCTGGGCAGAGACGGAGCAGGGGATATTCGAGCAGGTGCTGCACGGCACTCTGGACTTCGACTCGGACCCATGGCCGAGCGTGTCGGAGGGCGCAAAGGACCTGCTCAGGAAGGTGCTCGTCAGGGACCCCAAGAAGCGGCTCACAGCGCACCAAGTCCTATGCCACCCGTGGCTGCAGATGACTGGCGAGGCACCTGACAAGCCGCTCGACTCGGCGGTCCTGTCGCGGCTGAAGCAGTTCTCGGCGATGAACAAGCTCAAGAAGATGGCATTAAGAGTGATCGCGGAGAACTTGTCGGAGGAGGAGATCGCGGGGCTGAAGGAGATGTTCAAGATGATGGACACCGACAACAGCGGGCAGATCAATTACGAGGAGCTCAAGGCTGGGCTGGAGAGGGTGGGCGCCAACATGAAGGAGTCTGAGATATCTCAACTCATGCAGGCTGCTGATATTGACAATAGTGGCACCATTGATTATGGAGAGTTCATAGCTGCCACTCTACACCTCAACAAAGTTGAGAGGGAGGACCATCTGTATGCTGCCTTCCAGTACTTCGACAAGGATGGCAGCGGATACATCACGGCTGACGAGCTCCAGCAGGCCTGCGATGAGTTTGGGATCGAGGACGTTCGACTGGAGGACATGATAGGCGAAGTAGATCAGGACAATGATGGGCGCATAGATTACAATGAGTTCGTGGCCATGATGCAGAAATCAACCGCAGGGTTTGGGAAGAAAAGGCCATCAGTACAACCTTAG |
|  | Protein | MGNVCCVGPRRSFAKTRFFSALSSPIWRRSGAPAGSSSPSRPTVTTRSVPVVQPTASTPTPPPRASAVIKPPPEPTHTAPPAPIVISEPSRPQPPEPQPVVQNDPPHHHHHQQQEEQQQQQQAQSRPKKPTHIKRVSSAGLQVASVLRRKTENLKDKYSLGRKLGQGQFGTTYLCVDKASGQELACKSIAKRKLITDEDVEDVRREIQIMHHLAGHPSIISIRGAYEDAVAVHVVMELCAGGELFDRIVRRGHYTERQAAELARVIVAVVESCHSLGVMHRDLKPENFLFVGNEEDAALKTIDFGLSMFFRPGEMFTDVVGSPYYVAPEVLKKNYGQEADVWSAGVIVYILLCGVPPFWAETEQGIFEQVLHGTLDFDSDPWPSVSEGAKDLLRKVLVRDPKKRLTAHQVLCHPWLQMTGEAPDKPLDSAVLSRLKQFSAMNKLKKMALRVIAENLSEEEIAGLKEMFKMMDTDNSGQINYEELKAGLERVGANMKESEISQLMQAADIDNSGTIDYGEFIAATLHLNKVEREDHLYAAFQYFDKDGSGYITADELQQACDEFGIEDVRLEDMIGEVDQDNDGRIDYNEFVAMMQKSTAGFGKKRPSVQP |
| BdCDPK23 | CDS | ATGCAGACGGACGCGAGCAGCAACGCTACCGGCGGCGGCGGCGCGAATGCTGCGAGGCCGACGCTGCCGCCGCCGGTGACCGCCGCGCCGGCGGCGTCGTCCGGGAGGCCGGCGTCGGTGCTGCCGCACAAGACGGCCAACGTACGCGACCACTACCGCATCGGGAAGAAGCTGGGGCAGGGGCAGTTCGGCACCACGTACCAGTGCGTGGCCAAGGAGGGCGGGGGCGACTTCGCCTGCAAGTCCATCCCCAAGCGCAAGCTGCTGTGCCGCGAGGACTACGAGGACGCCTGGCGCGAGATCCAGATCATGCACCACCTCTCCGAGCACCCCAACGTCGTCCGCATCCGCGGCGCCTACGAGGACGCCCTCTTCGTGCACATTGTCATGGAGCTCTGTGCCGGCGGCGAGCTCTTCGACCGCATCGTGGCCAAGGGGCACTACAGCGAGCGCGCCGCCGCACAGCTCATCAGGACAATCGTCGGGGTCGTGGAGGGATGCCACTCGCTCGGCGTCATGCACCGGGACCTCAAGCCGGAGAACTTCCTGTTCGCCAGCACCGCTGAGGACGCGCCGCTCAAGACCACCGATTTTGGGCTCTCCATGTTCTACAAGCCTGGTGATAAATTCTCTGATGTTGTTGGGAGCCCCTACTATGTTGCGCCTGAGGTGCTTCAGAAATGCTATGGGCCAGAAGCTGATGTCTGGAGCGCTGGGGTGATTCTGTACATTTTGCTATGTGGTGTTCCCCCATTTTGGGCAGAAACTGAAGCAGGAATCTTCAGGCAGATCCTTCGAGGCAAACTTGACTTTGAATCTGAACCCTGGCCTACTATCTCCGACAGTGCTAAAGATCTAGTCCGTAATATGCTTTGTCGGGATCCTACAAAGCGACTCACTGCTCACGAGGTTCTCTGTCACCCATGGATTGTTGATGATGCTGTGGCGCCTGATAAGCCTATTGATTCTGCTGTTTTGTCACGGCTGAAGCATTTTTCTGCAATGAACAAGCTCAAGAAGATGGCATTGAGGGTTATTGCTGAAAGTCTGTCTGAGGAAGAGATTGGAGGCCTAAAGGAGCTGTTCAAAATGATTGATACTGACAATAGTGGGACCATAACATTTGATGAGCTGAAAGATGGCTTGAAAAGAGTGGGCTCAGAATTGACCGAACATGAAATCCAGGCTTTAATGGATGCAGCGGATATCGACAACAGTGGTACTATCGATTATGGTGAATTCCTCGCAGCTACATTGCACATGAACAAACTGGAGAGGGAGGAGAACTTGGTATCAGCATTCTCATTTTTCGACAAGGATGGAAGTGGCTTCATAACCATCGACGAGCTATCACAAGCATGCCATGAATTCGGTCTGGATGACGTCCACCTTGAGGATATGATCAAAGATGTCGATCAGAACAATGATGGGCAAATCGATTACAGCGAGTTCACGGCGATGATGAGGAAGGGCAATGCCGGCGCCACAGGGAGGCGTACCATGAGGAACAGCTTGCATCTGAATCTCGGCGACATCTTGAATCCCAGCAACAACTAA |
|  | Protein | MQTDASSNATGGGGANAARPTLPPPVTAAPAASSGRPASVLPHKTANVRDHYRIGKKLGQGQFGTTYQCVAKEGGGDFACKSIPKRKLLCREDYEDAWREIQIMHHLSEHPNVVRIRGAYEDALFVHIVMELCAGGELFDRIVAKGHYSERAAAQLIRTIVGVVEGCHSLGVMHRDLKPENFLFASTAEDAPLKTTDFGLSMFYKPGDKFSDVVGSPYYVAPEVLQKCYGPEADVWSAGVILYILLCGVPPFWAETEAGIFRQILRGKLDFESEPWPTISDSAKDLVRNMLCRDPTKRLTAHEVLCHPWIVDDAVAPDKPIDSAVLSRLKHFSAMNKLKKMALRVIAESLSEEEIGGLKELFKMIDTDNSGTITFDELKDGLKRVGSELTEHEIQALMDAADIDNSGTIDYGEFLAATLHMNKLEREENLVSAFSFFDKDGSGFITIDELSQACHEFGLDDVHLEDMIKDVDQNNDGQIDYSEFTAMMRKGNAGATGRRTMRNSLHLNLGDILNPSNN |
| BdCDPK24 | CDS | ATGGGCCAATGCTGCTCCAAGGGCGCCGCCGCCGCCGCTGCCGCCGACGACCCGGCCGCCGCCCCTCCTCCTCCTCCTGCAGCATCGGCCCCACGAGTGGAGCCCACCGCGTCCTCCTCCTCAGCCTCCAACCGCGGCGGCGCATCGAAGGCGGCGCCGCCATCGTCCTCGGCTCCGGTGGGCGAGGTCCTGGGCCGGCCCATGGAGGACGTGCGGGCCACCTACAGCATCGGCAAGGAGCTGGGCCGCGGCCAGTTCGGCGTGACCCACCTCTGCACCCACCGGACCTCCGGCGAGAAGCTGGCCTGCAAGACCATCGCCAAGCGGAAGCTCTCCACCAGAGAAGACCTCGAGGACGTCCGGCGGGAGGTCCAGATCATGTACCACCTGTCCGGGCAGCCCAACATCGTGGACCTCCGCGGCGCCTACGAGGACAAGCACAGCGTCCACCTGGTCATGGAGCTCTGCGCCGGCGGCGAGCTCTTCGACCGGATCATCGCCAGGGGCCACTACACGGAGCGCGCCGCCGCATCGCTCCTCCGCGCCATCGTCGGCATCGTCCACACCTGCCACTGCATGGGGGTCATGCACCGGGACCTCAAGCCCGAGAACTTCCTCCTGCTAGGGAAAGCCGACGACGCGCCGCTAAAGGCCACCGACTTCGGCCTCTCCGTCTTCTTCAAGGAAGGCGAGGTGTTCCGGGACATTGTCGGCAGCGCATACTACATCGCGCCGGAGGTGCTCAAAAGGCGGTACGGGCCCGAGGCTGATATATGGAGCATTGGTGTCATGCTATACATCTTCCTTGCCGGCGTGCCGCCTTTTTGGGCGGAGAATGAGAATGCGATTTTCACCGCTGTTTTGCGGGGGCACGTCGACTTCTCCGGCGATCCGTGGCCCGCCATCTCTGCTGGTGCGAAGGATCTTGTCAAGAAGATGCTCAATATTAACCCCAAGGAGAGGCTCACTGCATTTCAAGTCCTCAATCATCCGTGGATCAAAGAAGACGGTGACGCGCCCGACACGCCGCTCGACAACGTGGTCCTCAACAGGCTCAAGCAGTTCAGGGCCATGAACCAGTTCAAGAAGGCTGCGCTGAGGGTCATAGCCGGGTGCTTATCCGAGGAGGAGATCAAGGGGCTCAAGGAGATGTTCAAGAACATCGACAAGGACAACAGCGGCACCATCACGCTCGAAGAGCTCAAGAACGGCCTCGCCAAGCAGGGCACCAAGCTGTCCGATAATGAGATCGAGCAACTCATGGAAGCTGCCGATGCGGACGGCAACGGGTTGATCGACTACGAGGAGTTCGTCACCGCCACGGTGCACATGAACAAGATGGACAGAGAGGAGCACCTCTACACCGCGTTCCAGTACTTTGACAAGGATAATAGTGGGTTCATCACGAGAGACGAGCTCGAGCAAGCCTTGAAAGAGAAAGGTTTGTACGACGCCCAAGAGATCAAGGAGGTCATCTCCGAAGCTGACACTGACAATGACGGGAGGATAGACTATTCAGAGTTCGTGGCGATGATGAGGAAAGGAACAGGCACCGCCGAGCCAACGAACCCGAAGAAGAGGAGAGATCTAGTCCTATAG |
|  | Protein | MGQCCSKGAAAAAAADDPAAAPPPPPAASAPRVEPTASSSSASNRGGASKAAPPSSSAPVGEVLGRPMEDVRATYSIGKELGRGQFGVTHLCTHRTSGEKLACKTIAKRKLSTREDLEDVRREVQIMYHLSGQPNIVDLRGAYEDKHSVHLVMELCAGGELFDRIIARGHYTERAAASLLRAIVGIVHTCHCMGVMHRDLKPENFLLLGKADDAPLKATDFGLSVFFKEGEVFRDIVGSAYYIAPEVLKRRYGPEADIWSIGVMLYIFLAGVPPFWAENENAIFTAVLRGHVDFSGDPWPAISAGAKDLVKKMLNINPKERLTAFQVLNHPWIKEDGDAPDTPLDNVVLNRLKQFRAMNQFKKAALRVIAGCLSEEEIKGLKEMFKNIDKDNSGTITLEELKNGLAKQGTKLSDNEIEQLMEAADADGNGLIDYEEFVTATVHMNKMDREEHLYTAFQYFDKDNSGFITRDELEQALKEKGLYDAQEIKEVISEADTDNDGRIDYSEFVAMMRKGTGTAEPTNPKKRRDLVL |
| BdCDPK25 | CDS | ATGGGCGGTTGCTACTCCGTCATCGCGGCCTCCAGGATGCTGAACCGCAGGCGCGCCGCCATCCTCCCCCTGGGAAGCGGCGACGAGCAGATGGCCGGCGGCGGCTGCTCCCCCGCTAACGACAACGACAGCAGCAGCAAGAAGAAGCAGCGGATCAGGGGATTAAGGTGGAGGAGGAGCACCGCCATCCTGGGCGCCCTCGACGACGGCGCCGGCGACAGCCAGGCGGGGCCGGGGTCGTGTCAGTGCTTCTCGAAGCGGTACAGGCTGGGCGCGGAGCTGGGGCGCGGGGAGTTCGGGGTGACGCGGCGGTGCGTGGACGCGGCGACGGGCGAGGCGCTGGCGTGCAAGACCATCCGGAGGAAGCGGCTGCGGCGGGGCGCGGACGCCGAGGACGTGCGGCGCGAGGTGGAGATCCTGCGGCGCCTGTCCGGTGTCGGTGTCGGTGGCGCCGGAGGCTCGGGCGAAGGAGAAGGCGTGGTGGTGCGGCTCCGGGAGGCGTGCGAGGACGGCAAGGGCGTGCACCTCGTCATGGAGCTCTGCGAGGGCGGCGAGCTCTTCGACCGCATCTTCGCGCGGGGGCACTACACCGAGCGCGCCGCCGCCAAGATCGGCCGGACCATCGCCGAGGTCGTGCAGCTGTGCCACGAGAACGGCGTGATGCACAGGGACCTCAAGCCGGAGAACTTCCTGTTCGCCAACAAGTCCGAGGACTCCTGTCTCAAGGCAATCGACTTTGGCCTCTCAGTCTTCTTCAAGCCAGGAGATCGGTTCACGGAAGTGGTCGGCAGCGGGTTCTACATGGCGCCGGAGGTCCTCATGAGAAGCTACGGCCCGGAGGCGGATGTGTGGAGCGCCGGTGTCATCATCTACATCCTCCTGTGCGGAGTCCCTCCTTTCTGGGGAGACACAGATGAACGAATTGCAGAGTCGATAATCCAGGGTGGGATCAATTTCCAGAAGGAGCCATGGCCCAAGGTCTCCCTGACTGCGAAGGACCTTGTCAAGAAGATGCTTGACCCAAATCCTTCTACCCGGTTGACGGCAAAGGAAGTCCTTGAGCATCCATGGATCAAGAATGCCGATAAGGCTCCAAATGTGTCGCTCGGAGAGCTTGTTCGATCCAGGCTGAAGCAATTCTCATCCATGAACAAGTTCAAAAAGAAGGCACTTGGTATCGTTGCCAAGAATTTACCGGTGGAGGAGATCAACAACTACACTCAGATGTTCCATACGATGGACAAGGACAAGAACGGTAGTTTGACGCTTGAGGAGCTCAAGGAGGGCCTCTGGATAAATGGTCATCCTGTTCCAGAGACGGAGATACATATGCTGTTAGAGGCTGGTGACATAGATGGTAATGGCACATTAGACTGCGAGGAGTTTGTAACAGTCTTACTTCACATAAAAAAGATGAGCAACGAGGAGTACCTACCTAAAGCTTTCAAATACTTTGACAAAGATGGGGATGGATTTATTGAAATGGAAGAGTTGATGGAGGCTTTAGCTGATGATGAACTAGGCCCTAATGAGCAAGTGGTTAAAGATATTATATGTGATGTTGACACGGATAAGGATGGTCGCATTAGTTATCATGAGTTTGAAGTGATGATGATATCTGGATCAGACTGGAGGAATGCTTCTCGGCGGTTCTCAAGAGCAAATTTCAGCTCCCTTAGTTACAAGCTGTGCCAATGA |
|  | Protein | MGGCYSVIAASRMLNRRRAAILPLGSGDEQMAGGGCSPANDNDSSSKKKQRIRGLRWRRSTAILGALDDGAGDSQAGPGSCQCFSKRYRLGAELGRGEFGVTRRCVDAATGEALACKTIRRKRLRRGADAEDVRREVEILRRLSGVGVGGAGGSGEGEGVVVRLREACEDGKGVHLVMELCEGGELFDRIFARGHYTERAAAKIGRTIAEVVQLCHENGVMHRDLKPENFLFANKSEDSCLKAIDFGLSVFFKPGDRFTEVVGSGFYMAPEVLMRSYGPEADVWSAGVIIYILLCGVPPFWGDTDERIAESIIQGGINFQKEPWPKVSLTAKDLVKKMLDPNPSTRLTAKEVLEHPWIKNADKAPNVSLGELVRSRLKQFSSMNKFKKKALGIVAKNLPVEEINNYTQMFHTMDKDKNGSLTLEELKEGLWINGHPVPETEIHMLLEAGDIDGNGTLDCEEFVTVLLHIKKMSNEEYLPKAFKYFDKDGDGFIEMEELMEALADDELGPNEQVVKDIICDVDTDKDGRISYHEFEVMMISGSDWRNASRRFSRANFSSLSYKLCQ |
| BdCDPK26 | CDS | ATGGGCAACTGCTGCGTTGCCCGGCCGTCCTTCAGGCGCCGCGGCGGCGGCGGCGGAGGTGGCTCCCCCCGGCACCGCGGCGGGCGTCTCGGCGGCGCCGGCAACCTCCGCTGCCTCTCCACCATCTCCTCCGTCACCGACACCCCCCGCGCCGCCGCCGCCCCCATCACCGTCCTCAACAGCAAATCCCTCGCCCCCCCAGAGGCCGCGTCCTCCGCCGCCGAGGAGCTTCTCCGCCGGTACGTCCTGGGCGAGGAGCTGGGCCGGGGCGAGTTCGGCGTGACCCGCCGCTGCACGGACTCAACCACCTCCCAGACCCTCGCCTGCAAGTCCATCAGCAAGCGGAAGCTCCGGAGCAGCGTGGACGTGGAGGACGTGCGGCGCGAGGTCTCCATCATGCGCGCTCTGCCCGCCCACCCAAACGTCGTCGCGCTCCGGGAAGCCTTCGAGGACTCGGACGCCGTGCACCTGGTCATGGAGGTCTGCGAGGGCGGGGAGCTCTTCGACCGCATCGTCGCCAAGGGCCACTACACGGAGCGCGCGGCCGCCGGCGTCATGCGCACCATCATGGAGGTCGTGGGGCATTGCCATAGGAACGGGGTCATGCATCGGGATCTCAAGCCTGAGAACTTCTTATATGCGAATGCGAGCGAGGCGTCGCCGCTTAAAGTCATCGACTTCGGGCTGTCCGTATGCTTCAAGCCGGGTGAAAGGTTCAGCGAGATCGTCGGGTCGCCGTATTACATGGCGCCCGAGGTGCTCAAGAGGAACTATGGCCAGGAAATTGATGTCTGGAGCGCTGGGGTCATACTCTACATCTTGCTGTGTGGTGTCCCTCCATTCTGGGCCGAGAGCGACGAAGGGATCGCGCAGGCCATCATCCGGGCGCGCCTCGACTTCGAGCGGGAGCCGTGGCCCAAGGTGTCGGACAACGCCAAGGACCTCGTCAGGAAAATGCTCGACCCCAACCCTTACGCCAGGCTCACGGCTCAGCAGGCTCTAGAGCACCCTTGGATACAGAATGCCAGTGTTGCCCCTAACATTCCTCTTGGAGAAGCAGTAAGGTCCAGGCTGAAGCAGTTCACGGTCATGAACAAGTTCAAGAAGAAGGCCCTACTTGTGGTGGCGGAGTACTTGCCGGCAGAGGAGCTGGAGGCGATCACGGAGCTGTTCCACATGCTGGACACCAACAACGACGGGCACCTGACGATCGAGGAGCTCAGGAAAGGATTACAGATGATAGGGAACAACGTCAATGACACCGACGTGGACATGCTCATGGAAGCTGCAGACATAGACGGGAATGGCACCCTGGACTGCAAGGAGTTCGTGACAGTGTCCATCCACCTGAAGAAGATCCGCGGCGAGGAGCACCTCCCCAAGGTGTTCAACTACTTCGACAAGAACATGAGCGGGTTCATCGAGATGGAGGAGCTCAAGGAGGCGCTGTCCCCGAGAGGCGACCAGAAGGCCATCGAAGACATCATCTTCGACATCGACATCGACAAGGACGGGAAGATAAGCTACGAGGAGTTCGAGCTGATGATGAAGGCCGGAGTGGACTGGAGGAACGCGTCGAGGCAGTACTCGAGAGCGGTTTTCAACACCCTGAGCCGAAAGATGTTCAAGGAGACGTCCCTGAAGATTCTTGATCCCATCACCCCACGTGGCGCAGCCGCCACCGTGGCGAAACAACAACAGGACATGATCTGA |
|  | Protein | MGNCCVARPSFRRRGGGGGGGSPRHRGGRLGGAGNLRCLSTISSVTDTPRAAAAPITVLNSKSLAPPEAASSAAEELLRRYVLGEELGRGEFGVTRRCTDSTTSQTLACKSISKRKLRSSVDVEDVRREVSIMRALPAHPNVVALREAFEDSDAVHLVMEVCEGGELFDRIVAKGHYTERAAAGVMRTIMEVVGHCHRNGVMHRDLKPENFLYANASEASPLKVIDFGLSVCFKPGERFSEIVGSPYYMAPEVLKRNYGQEIDVWSAGVILYILLCGVPPFWAESDEGIAQAIIRARLDFEREPWPKVSDNAKDLVRKMLDPNPYARLTAQQALEHPWIQNASVAPNIPLGEAVRSRLKQFTVMNKFKKKALLVVAEYLPAEELEAITELFHMLDTNNDGHLTIEELRKGLQMIGNNVNDTDVDMLMEAADIDGNGTLDCKEFVTVSIHLKKIRGEEHLPKVFNYFDKNMSGFIEMEELKEALSPRGDQKAIEDIIFDIDIDKDGKISYEEFELMMKAGVDWRNASRQYSRAVFNTLSRKMFKETSLKILDPITPRGAAATVAKQQQDMI |
| BdCDPK27 | CDS | ATGCAGCCGGACCCGAAAGGCCCTGGCAGGGGGAAGGGGGGCGGCAATGCGCACGCGCGGCTGCCGCCGCCGGTGACGGCGCCGTCGGTGGGGCGGCCGGCGTCGGTGCTGCCGCACAAGACGGCCAACGTGCGCGACCACTACCGCATCGGGAAGAAGCTGGGGCAAGGGCAGTTCGGCACCACGTACCTGTGCGTGGCCAAGGAGGACGGCGGCGAGTACGCCTGCAAATCCATCCCCAAGCGGAAGCTGCTGTGCCGGGAGGACTACGAGGACGTGTGGCGCGAGATCCAGATCATGCACCACCTCTCCGAGCACCCCAACGTCGTCCGCATCCGGGGCGCCTACGAGGACGCCCTCTTCGTGCACCTCGTCATGGAGCTCTGCGCCGGCGGCGAGCTGTTCGACCGCATCGTCGCCAAGGGACATTACAGCGAGCGCGCCGCCGCGCAGCTCATCAGGACCATCGTCGGGGTGGTGGAGGGGTGCCACTCGCTTGGCGTCATGCACCGGGACCTCAAGCCGGAGAATTTCCTGTTCGCGAGCACCGCCGAGGACTCCCCGCTCAAGGCCACCGACTTTGGGCTCTCCATGTTCTATAAGCCCGGCGATAAATTTGCAGATGTTGTTGGCAGCCCCTATTATGTTGCACCTGAGGTGCTCCTAAAAAGCTATGGCATGGAAGCTGATGTCTGGAGTGCCGGAGTAATTCTGTACATTTTGCTCTGTGGTGTGCCGCCATTTTGGGCAGAAAGTGAGTCAGGAATCTTCAGGCAGATTCTGCGAGGTAAACTTGACTTGGAAACTGAACCATGGCCTAGTATCTCTGATAGTGCTAAAGATCTAGTTCGTAAGATGCTTACCCGAGATCCTACAAAGAGACACACTGCTCATGAAGTTCTATGTCATCCATGGATTGTTGATGATTCTGTAGCACCTGATAAACCTATTGATTCTGCTGTTTTGTCAAGACTGAAACACTTCTCTGCAATGAACAAACTCAAGAAGATGGCATTGAGGGTAATTGCTGAAAGTCTATCTGAGGAGGAGATCGGTGGCTTAAAGGAATTGTTCAAAATGATTGACACCGACAATAGTGGGACGATAACTTATGAAGAACTGAAGGATGGCTTGAAAAGGGTGGGCTCTGATTTAATGGAACCTGAAATCCAGTCTTTAATGGATGCGGCTGATATTGACAACAGTGGAAGCATTGACTACGGTGAATTCTTAGCGGCTACGTTGCACGTGAATAAACTGGAAAGGGAGGAAAATTTGGTGTCAGCATTCTCATTCTTCGATAAGGATGGAAGTGGCTTCATAACAATTGATGAGCTCTCACAAGCATGCGAAAAGTTTGGTCTTTCTGATGTTCATCTTGAGGATATGATGAAAGACGTGGATCAAAACAATGATGGACAAATTGATTACAGCGAGTTTGCCGCAATGATGAGGAAGGGTAATGCTGGCGGATCTGGCACTGTTAGTGGAGCTGTTGGGACAGGAAGGAGAACCATGAGGAACAGCCTGCTTGTGAATCTCGGTGATATCTTCAGACCAGGCGAAAACTAA |
|  | Protein | MQPDPKGPGRGKGGGNAHARLPPPVTAPSVGRPASVLPHKTANVRDHYRIGKKLGQGQFGTTYLCVAKEDGGEYACKSIPKRKLLCREDYEDVWREIQIMHHLSEHPNVVRIRGAYEDALFVHLVMELCAGGELFDRIVAKGHYSERAAAQLIRTIVGVVEGCHSLGVMHRDLKPENFLFASTAEDSPLKATDFGLSMFYKPGDKFADVVGSPYYVAPEVLLKSYGMEADVWSAGVILYILLCGVPPFWAESESGIFRQILRGKLDLETEPWPSISDSAKDLVRKMLTRDPTKRHTAHEVLCHPWIVDDSVAPDKPIDSAVLSRLKHFSAMNKLKKMALRVIAESLSEEEIGGLKELFKMIDTDNSGTITYEELKDGLKRVGSDLMEPEIQSLMDAADIDNSGSIDYGEFLAATLHVNKLEREENLVSAFSFFDKDGSGFITIDELSQACEKFGLSDVHLEDMMKDVDQNNDGQIDYSEFAAMMRKGNAGGSGTVSGAVGTGRRTMRNSLLVNLGDIFRPGEN |
| BdCDPK28 | CDS | ATGGGCCAATGCTGCACCACCGGCGCCGCCCAGGCTGCCGCCGTCGACGCCGCTGCCGAGGCCGAAGCCCCGGGCCCCCCAAAGTCCGACTCCCCACGCGCCGGGGCCGACGCCGCCCCCAATGCCAAAGCGGACACCAATGCCGCCCCCAATGCCGATGCCAACGCCAAAGCCGCCCCCAATGCCGACGCCGACGCCAAGCCATCATCGTCTTCCTCGGCGCCGGTGGGCGAGGTGCTCGGCAACCCGATAGAGGACGTGCGCGCGACGTACACCATCGGCGAAGAGCTCGGGCGCGGCCAGTTCGGGGTGACATACCTCTGCACGCACTCCATCACCGGCGAGAAGCTGGCCTGCAAGACGATCGCCAAGCGGAAACTCTCCGGCAAAGAGGATGTGGAGGACGTCCGGCGGGAGGTGGCCATCATGCGGCACCTGGCGGGGCAGCCCAACATCGTGGCCCTCCGGGGCGCCTACGAGGACAAGCACAACGTCCACCTGGTCATGGAGCTCTGCGCCGGCGGGGAGCTCTTCGACCGGATCATCGCCAAGGGACACTACACGGAGCGCGCCGCGGCGTCGCTGCTCCGGACCGTGGTCGGCACGGTCCAGACGTGCCATGCCAGGGGCGTCATGCACCGGGATCTCAAGCCGGAGAACTTCCTCATGCTCAGCCGGGACGAGTCCTCCGCCATCAAGGCCACCGATTTCGGGCTCTCCGTCTTCTTCAAGGACGGCGAGACCTTCAACGACATCGTCGGCAGCGCGTACTATATCGCGCCCGAGGTGCTCAAGAGGAAGTATGGGCCAGAGGCCGACGTGTGGAGCATTGGTGTCATGCTCTACATTTTTCTCTCCGGCGTGCCGCCGTTCTGGGCTGAGACGGAGAATGCCATCTTTACCGCCATTTTGCGCGGCGAGGTCGATTTTGTCACTGATCCTTGGCCTAGTATCTCTAACGGGGCTAAGGATCTCGTCAGGAAGATGCTCCATGTTGACCCCAAGGAGAGGCTCACCGCCATCCAAGTCCTCAATCACCCTTGGATCAAGGAAGACGGAGACGCGCCCGACACGCCGCTCGACGACGTCGTGCTCGACAGGATGAAGCAGTTCAGGGCCATGAACCAGTTCAAGAAAGCTGCACTCAGGGTCATAGCCGGGTGCCTGTCGGAGGAGGAGATCAATGGGCTCAAGGAGATGTTCAAGAACATGGACAAGGACAACAGCGGCACCATCACGCTCGAGGAGCTCAAGAATGGGCTGGCCAAGCAAGGCACCAAGCTGTCAGACCATGAGATTCAGCAACTGATGGAAGCTGCCGACGCCGATGGCAATGGATTGATCGACTACGAGGAGTTCGTCACGGCCACGGTGCACATGAACAGAATGGACAGAGAGGAGCACCTTTACACTGCATTCCAGTACTTCGACAAGGACAACAGTGGGTTCATAACAGTAGAAGAGCTGGAGCAAGCCTTGCAGGAGCAAAAGCTGTACGACCCCAGCGAATTCAAGGAGGTCATCTCCGAAGCCGATTCTGACAATGATGGGAGGATAGATTATTCAGAGTTCGTGGCGATGATGAGGAAAGGAACAGGTGGCGCCGAGCCATCGAACCCGAAAAAGAGGAGAGACCTAGTCCTAGATTGA |
|  | Protein | MGQCCTTGAAQAAAVDAAAEAEAPGPPKSDSPRAGADAAPNAKADTNAAPNADANAKAAPNADADAKPSSSSSAPVGEVLGNPIEDVRATYTIGEELGRGQFGVTYLCTHSITGEKLACKTIAKRKLSGKEDVEDVRREVAIMRHLAGQPNIVALRGAYEDKHNVHLVMELCAGGELFDRIIAKGHYTERAAASLLRTVVGTVQTCHARGVMHRDLKPENFLMLSRDESSAIKATDFGLSVFFKDGETFNDIVGSAYYIAPEVLKRKYGPEADVWSIGVMLYIFLSGVPPFWAETENAIFTAILRGEVDFVTDPWPSISNGAKDLVRKMLHVDPKERLTAIQVLNHPWIKEDGDAPDTPLDDVVLDRMKQFRAMNQFKKAALRVIAGCLSEEEINGLKEMFKNMDKDNSGTITLEELKNGLAKQGTKLSDHEIQQLMEAADADGNGLIDYEEFVTATVHMNRMDREEHLYTAFQYFDKDNSGFITVEELEQALQEQKLYDPSEFKEVISEADSDNDGRIDYSEFVAMMRKGTGGAEPSNPKKRRDLVLD |
| BdCDPK29 | CDS | ATGGGGAACTGCTGCGCCAAGACGTACGAGATGGAGATACCCATCACGTCGGGGACGATGGAGCGGCCGCCGACGTTCGGCAACCACCCGCCGCCGCCCGCCGCCGGCCAGCGCAGCAAGGGGCAGGACCGGCCGCCGACGTCGTGGCGGCTGCCGACGTTCCCGAAGCAGGCGCCGCCGCCGTCCGGGCGGCCGGCGCTGCCGAGCTCCCTGACGGGGGTGTCCCAGTCCCGGAAGGCCGGGGGCGCGATGGGCCCGGTGCTGCAGAGGCCGATGGTGGACGTCCGGTCGCAGTACAACCTGGAGCGGAAGCTTGGGAGCGGGCAGTTCGGGACGACGTACCTGTGCACGGAGCGCGCCACGGGGCTCAAGTACGCCTGCAAGTCGGTGTCCAAGCGGAAGCTGCTGCGGCGGGCGGACGTGGAGGACATCCGCCGGGAGGTGACCATCCTGCAGCACCTCAGCGGGCAGCCCAACATCGCCGAGTTCAGGGGCGCCTTCGAGGACGGCGAGAGCGTGCACCTCGTCATGGAGTTCTGCTCCGGCGGGGAGCTCTTCGACCGCATCACCGCCAAAGGGAGCTACTCCGAGCGCCAGGCGGCCGCCGTGTGCCGGGACGTGCTCACCGTCGTCCACGTCTGCCACTTCATGGGGGTCATGCACCGGGACCTCAAGCCCGAGAACTTCCTGCTCGCCAGCCCCGCCGAGGATGCCCCGCTCAAGGCCATCGACTTCGGTCTCTCCGTCTTCATCGAAGAAGGAAAAGTGTACAAGGATATTGTGGGAAGTGCATACTATGTAGCACCAGAAGTATTGCGTCGAAATTACGGGAGGGAAATTGACGTCTGGAGCGCTGGAGTGATCTTGTATATTCTTTTATGTGGGTCGCCACCTTTCTGGGCAGGTAAATATTCATACATCTTCAGTTTACATGCTACATTGCTTATATACAGTATCGATCGCAGGGCTAAATATCCGAATTTGCAAATCTGTGCAGAAACAGAGAAGGGGATATTTGATGCTATACTGGTGGCCCAGCTTGATTTCAGTAGCAGCCCGTGGCCAACGATATCTGAAAACGCAAAGGATCTTATCAGACAAATGTTGAATACGGATCGCCAGAAGCGTATTACTGCAGCACAAGCCCTAGAACACCCATGGCTGAAAGAAGGCGGTGCATCTGACAGACCTATCGATAGTGCAGTCTTGTTAAGAATGAAGCAATTCAAGGCAATGAACAAGCTAAAGCAACTAGCACTTAAGGTAATGGCAGAAAACCTATCACCAGAAGAAATCAAGGGCTTGAAACAGATGTTCAACAACATGGATACAGACAAGAGTGGAACAATCACAGTTGAAGAACTAAAGATTGGTTTGACGAAGTTAGGATCAAAGATTAGCGAAGCAGAGGTCCAGAAGCTTCTGGAAGCGGTTGATGTAGACAAGAGTGGAAGCATTGACTACACTGAGTTTCTGACTGCTATGATGAATAAACATAAGATGGAAAAGGAAGAGGATTTGATCCGTGCATTTCAACACTTTGACAAAGATAACAGCGGGTACATATCAAGAGAGGAGCTTAAACAAGCTATGACAGAGTATGGAATTGGCGATGAAGCAAATATTAAAGAAGTACTAGATGAAGTTGACAAAGACAAGGATGGGAGAATCGACTATGAAGAGTTTGTGGAAATGATGAGGAAAGGAATATATACCTGA |
|  | Protein | MGNCCAKTYEMEIPITSGTMERPPTFGNHPPPPAAGQRSKGQDRPPTSWRLPTFPKQAPPPSGRPALPSSLTGVSQSRKAGGAMGPVLQRPMVDVRSQYNLERKLGSGQFGTTYLCTERATGLKYACKSVSKRKLLRRADVEDIRREVTILQHLSGQPNIAEFRGAFEDGESVHLVMEFCSGGELFDRITAKGSYSERQAAAVCRDVLTVVHVCHFMGVMHRDLKPENFLLASPAEDAPLKAIDFGLSVFIEEGKVYKDIVGSAYYVAPEVLRRNYGREIDVWSAGVILYILLCGSPPFWAGKYSYIFSLHATLLIYSIDRRAKYPNLQICAETEKGIFDAILVAQLDFSSSPWPTISENAKDLIRQMLNTDRQKRITAAQALEHPWLKEGGASDRPIDSAVLLRMKQFKAMNKLKQLALKVMAENLSPEEIKGLKQMFNNMDTDKSGTITVEELKIGLTKLGSKISEAEVQKLLEAVDVDKSGSIDYTEFLTAMMNKHKMEKEEDLIRAFQHFDKDNSGYISREELKQAMTEYGIGDEANIKEVLDEVDKDKDGRIDYEEFVEMMRKGIYT |
| BdCDPK30 | CDS | ATGGGCAACGCATGCGGCGGCTCCCTTAGATCCAAGTACCTGCACAGCTTCAAGCACGCCGCGTCGCAGCGCCACGACTCCGACTACAACAGCAGCGCCGCGGCCGCGGGAGCGGACTCGCCGAAGAAGCAGCAGCAGCCCTCCTCCCACCCGTCCGCAGCCGCCGCCAAGACGGACGGCCACGCGGCCCCCGCGCCCGCGCCGCCCGCCGCCATGAGGCGCGGCGGGGCCGGCGCGCCCGCCGACCTCGGCTCCGTGCTCGGCCACCCCACGCCCAGCCTCCGCGACCTCTACCAGCTCGGCAGGAAGCTCGGGCAGGGCCAGTTCGGCACCACCTTCCTCTGCACCGAGCTCGCCACGGGGACGGAGTACGCCTGCAAGTCCATCTCCAAGCGCAAGCTCATCACCAAGGAGGACATCGACGACGTGCGCCGCGAGATCCAGATCATGCACCATCTCTCCGGACACAACAACGTCGTCGCCATCAAGGGCGCCTACGAGGACCAGCTCTACGTGCACATCGTCATGGAGCTCTGCGCTGGCGGCGAGCTCTTCGACCGCATCATACAGCGCGGCCACTACAGCGAGCGCAAGGCCGCCGAGCTCACTCGGATCATCGTCGGGGTCGTCGAGGCCTGCCACTCGCTCGGGGTCATGCACCGGGACCTCAAGCCCGAGAACTTTCTGCTCGCCAACAAGGACGATGACCTCTCGCTCAAGGCCATCGATTTTGGGCTCTCCGTTTTCTTCAAGCCTGGCCAAATTTTCACCGATGTTGTCGGAAGCCCATATTATGTAGCTCCAGAAGTGCTCTGCAAAAAGTATGGACCAGAAGCTGATGTATGGACCGCTGGTGTAATTCTTTACATTCTACTAAGTGGTGTACCCCCGTTTTGGGCAGAGACACAACAAGGAATATTTGATGCTGTATTGAAGGGTGTCATTGATTTTGATTCTGAACCCTGGCCTGTGATATCTGACAGTGCAAAAGATCTGATAACACGAATGCTCAATCCCCGCCCTGCAGAACGCTTGACAGCACATGAAGTTCTATGCCATCCTTGGATTCGTGATCAGGGAGTCGCTCCTGATCGTCCTCTTGACACAGCTGTTCTATCTCGCATTAAGCAATTCTCTGCAATGAATAAGTTGAAGAAGATGGCTTTGCGGGTAATAGCCGAGAGCCTCTCAGAGGAGGAAATTGCTGGGTTGAAGGAAATGTTCCAGACTATGGACACTGATAACAGTGGTGCAATTACATATGATGAGCTCAAAGAAGGCTTGAAAAAATACGGCTCCACACTGAAGGATACTGAGATCCGTGATCTTATGGAAGCAGCGGATGTGGACAACAGTGGTACCATTGACTATATAGAGTTCATCGCTGCGACATTGCATCTGAATAAACTGGAGCGTGAGGAACATCTAGTGGCAGCCTTTTCATATTTTGACAAAGATGGAAGTGGCTACATCACAGTGGATGAACTGCAGCAAGCATGCCAAGAGCATAACATGCCAGATGCTTTTCTTGATGATGTCATTAAAGAAGCTGACCAGGACAATGATGGCCGCATTGACTATGGAGAATTTGTTGCCATGATGACCAAGGGCAATATGGGGGTTGGGCGAAGAACAATGAGAAATAGCTTGAATATCAGCATGAGAGATGCACCCGGTGCACTCTAG |
|  | Protein | MGNACGGSLRSKYLHSFKHAASQRHDSDYNSSAAAAGADSPKKQQQPSSHPSAAAAKTDGHAAPAPAPPAAMRRGGAGAPADLGSVLGHPTPSLRDLYQLGRKLGQGQFGTTFLCTELATGTEYACKSISKRKLITKEDIDDVRREIQIMHHLSGHNNVVAIKGAYEDQLYVHIVMELCAGGELFDRIIQRGHYSERKAAELTRIIVGVVEACHSLGVMHRDLKPENFLLANKDDDLSLKAIDFGLSVFFKPGQIFTDVVGSPYYVAPEVLCKKYGPEADVWTAGVILYILLSGVPPFWAETQQGIFDAVLKGVIDFDSEPWPVISDSAKDLITRMLNPRPAERLTAHEVLCHPWIRDQGVAPDRPLDTAVLSRIKQFSAMNKLKKMALRVIAESLSEEEIAGLKEMFQTMDTDNSGAITYDELKEGLKKYGSTLKDTEIRDLMEAADVDNSGTIDYIEFIAATLHLNKLEREEHLVAAFSYFDKDGSGYITVDELQQACQEHNMPDAFLDDVIKEADQDNDGRIDYGEFVAMMTKGNMGVGRRTMRNSLNISMRDAPGAL |
