# Supplementary material for: Genome-wide survey and expression analysis of calcium-dependent protein kinase (CDPK) in grass Brachypodium distachyon
Source: BMC Genomics. 2020 Jan 16;21:53. doi: 10.1186/s12864-020-6475-6 (PMC6966850; doi:10.1186/s12864-020-6475-6)
Supplement: Supplementary file 12 — Additional file 12. List of marker genes and qRT-PCR primers of marker genes. [file 12864_2020_6475_MOESM12_ESM.docx]

Additional file 12 List of marker genes and qRT-PCR primers

| Treatment | *B. distachyon*\Rice \Arabidopsis gene name | *B. distachyon* Gene ID | Forward Primer | Reverse Primer |
| --- | --- | --- | --- | --- |
| 6-BA | *OsRHT-1* | *Bradi4g18390* | TCTCTTCCCCAAGGAGATCA | CGCTGTTACTGTGGGCATTA |
| 6-BA | *OsRR10* | *Bradi4g43090* | AAGCAGATGCAAGGAGCATT | CGTCTTCTGTCGCCTTTCTC |
| ABA | *BdPP2CA4* | *Bradi2g18510* | AAATCCAGCAGTGGCACAA | CCTCCGAGGTTTCAAGTCAA |
| ABA | *BdPP2CA6* | *Bradi2g45470* | GCCAGACAGACCTGATGAGATG | TGACCTAGAAGTCGCAAGCAC |
| GA | *-* | *Bradi3g07160* | TCCATCGACCTCCCCTTC | GCCGGGTTCATCGAGTTA |
| GA | *AtEXP1* | *Bradi2g22290* | CTTCCTCCACATCGCTCAGT | AGTTCCTCGACATGGTCTGC |
| NAA | *AtAux/IAA9* | *Bradi2g31820* | GAACACTATGGCGACCAACC | ATCCTTGCCAGTTGTGAAGC |
| NAA | *AtAux/IAA18* | *Bradi2g33417* | CACGTTGCTATCAGCCAGAA | CCGACTGACCAAGATTCCTC |
| Cold | *BdCBF1* | *Bradi3g51630* | ACATGACTGTGTCTTCCGCC | ATGTCCCGAGCCATATCCTC |
| Cold | *BdCBF2* | *Bradi1g49560* | CTACGAAGATGACGGCGGAG | TTCTTGTTGGGCTCCCTGAC |
| Heat | *BdHsf02* | *Bradi1g05550* | ACTGAGCAGAAGCAACAGCA | ACGGAGAGCCATTGATGAAC |
| Heat | *BdHsp70-1* | *Bradi1g66590* | GCTCGTCGGAGGATCTACAC | GTCCTGCACTTTCTCGTTGC |
| H_2_O_2_ | *AtAPX1* | *Bradi1g16510* | CCTGAGTGGCGAGAAGGAAG | TACCCGCCAAATCCCAACTC |
| H_2_O_2_ | *AtAPX2* | *Bradi1g65820* | GCTACCAAGGGTTCTGACCA | GGCCCTCTTTGTCACCACT |
| NaCl | *BdCBF2* | *Bradi1g49560* | CTACGAAGATGACGGCGGAG | TTCTTGTTGGGCTCCCTGAC |
| NaCl | *BdP5CS1* | *Bradi2g54920* | CAAGCGCATCGTCATCAAGG | GTACTTGAGCCTCTGCCTCC |
| PEG | *BdPP2CA6* | *Bradi2g45470* | GCCAGACAGACCTGATGAGATG | TGACCTAGAAGTCGCAAGCAC |
| PEG | *BdCBF2* | *Bradi1g49560* | CTACGAAGATGACGGCGGAG | TTCTTGTTGGGCTCCCTGAC |
