# Supplementary material for: Genome-wide survey and expression analysis of calcium-dependent protein kinase (CDPK) in grass Brachypodium distachyon
Source: BMC Genomics. 2020 Jan 16;21:53. doi: 10.1186/s12864-020-6475-6 (PMC6966850; doi:10.1186/s12864-020-6475-6)
Supplement: Supplementary file 14 — Additional file 14 Homologous gene of BdCDPKs in responsive to hormone and abiotic stress. [file 12864_2020_6475_MOESM14_ESM.docx]

Additional file 14 Homologous gene of BdCDPKs in responsive to hormone and abiotic stress

| **Gene name** | **Species** | **Homologous gene** | **Response to hormone and abiotic stress^a^** |
| --- | --- | --- | --- |
| BdCDPK01 | Arabidopsis | At5g19450 | Drought[[1](#_ENREF_1)] |
|  | Rice | LOC_Os03g59390 | ND |
|  | Grape | VIT_208s0007g08300 | Salt[[2](#_ENREF_2)] |
|  | Maize | GRMZM2G104125_T01 | ND |
| BdCDPK02 | Arabidopsis | At5g04870 | Salt/drought[[3](#_ENREF_3)], SA[[4](#_ENREF_4)] |
|  | Rice | LOC_Os03g57510 | ND |
|  | Grape | VIT_208s0032g00780 | ND |
|  | Maize | GRMZM2G027351_T01 | ND |
| BdCDPK03 | Arabidopsis | At5g04870 | Salt/drought[[3](#_ENREF_3)] , SA[[4](#_ENREF_4)] |
|  | Rice | LOC_Os03g57450 | Drought, ROS[[5](#_ENREF_5)], cold, salt[[6](#_ENREF_6)] |
|  | Grape | VIT_213s0175g00080 | Drought[[2](#_ENREF_2)] |
|  | Maize | GRMZM2G320506_T01 | ND |
| BdCDPK04 | Arabidopsis | At1g18890 | Drought[[7](#_ENREF_7)], high salinity[[7](#_ENREF_7)], ABA[[8](#_ENREF_8)] |
|  | Arabidopsis | At1g74740 | Salt[[9](#_ENREF_9)] |
|  | Rice | LOC_Os03g48270 | Drought[[10](#_ENREF_10)] |
|  | Grape | VIT_208s0007g08300 | Salt[[2](#_ENREF_2)] |
|  | Maize | GRMZM2G030673_T01 | ND |
| BdCDPK05 | Arabidopsis | At5g19450 | Drought[[1](#_ENREF_1)] |
|  | Rice | LOC_Os07g38120 | SA and JA[[11](#_ENREF_11)] |
|  | Grape | VIT_208s0007g08300 | Salt[[2](#_ENREF_2)] |
|  | Maize | GRMZM2G028086_T01 | ND |
| BdCDPK06 | Arabidopsis | At3g20410 | ABA[[12](#_ENREF_12)] |
|  | Arabidopsis | At1g50700 | ABA[[12](#_ENREF_12), [13](#_ENREF_13)] |
|  | Rice | LOC_Os07g33110 | ND |
|  | Grape | VIT_219s0090g00410 | Drought, salt[[2](#_ENREF_2)] |
|  | Maize | GRMZM2G168706_T01 | ND |
| BdCDPK07 | Arabidopsis | At5g66210 | GA[[14](#_ENREF_14)], JA[[15](#_ENREF_15)] |
|  | Rice | LOC_Os07g22710 | ND |
|  | Grape | VIT_204s0023g03420 | ND |
|  | Maize | GRMZM2G365035_T01 | ND |
| BdCDPK08 | Arabidopsis | At5g04870 | Salt/drought[[3](#_ENREF_3)] , SA[[4](#_ENREF_4)] |
|  | Rice | LOC_Os07g06740 | Cold[[6](#_ENREF_6), [16](#_ENREF_16), [17](#_ENREF_17)], dehydration,salt[[6](#_ENREF_6)] |
|  | Grape | VIT_213s0175g00080 | Drought[[2](#_ENREF_2)] |
|  | Maize | GRMZM2G012326_T01 | ND |
| BdCDPK09 | Arabidopsis | At2g17290 | ABA[[18](#_ENREF_18)], salt/drought[[19](#_ENREF_19)], MeJA[[20](#_ENREF_20)] |
|  | Rice | LOC_Os03g03660 | Cold[[21](#_ENREF_21), [22](#_ENREF_22)], drought[[22](#_ENREF_22)], GA[[21](#_ENREF_21)] |
|  | Grape | VIT_203s0038g03960 | ND |
|  | Maize | GRMZM2G032852_T02 | ND |
| BdCDPK10 | Arabidopsis | At4g23650 | Auxin[[23](#_ENREF_23)], ABA[[18](#_ENREF_18)], salt[[24](#_ENREF_24)] |
|  | Rice | LOC_Os05g50810 | ND |
|  | Grape | VIT_202s0025g00690 | ND |
|  | Maize | GRMZM2G058305_T01 | ND |
| BdCDPK11 | Arabidopsis | At5g19360 | ND |
|  | Rice | LOC_Os01g59360 | ND |
|  | Grape | VIT_206s0009g03150 | ND |
|  | Maize | GRMZM2G340224_T01 | ND |
| BdCDPK12 | Rice | LOC_Os05g41090 | ND |
|  | Grape | VIT_213s0047g00260 | ND |
|  | Maize | GRMZM2G062772_T01 | ND |
| BdCDPK13 | Arabidopsis | At3g57530 | ABA and salt[[25](#_ENREF_25)] |
|  | Rice | LOC_Os05g39090 | ND |
|  | Grape | VIT_208s0105g00390 | ND |
|  | Maize | GRMZM2G088361_T01 | ND |
| BdCDPK14 | Arabidopsis | At4g23650 | Auxin[[23](#_ENREF_23)], ABA[[18](#_ENREF_18)] , salt[[24](#_ENREF_24)] |
|  | Rice | LOC_Os01g43410 | ND |
|  | Grape | VIT_202s0025g00690 | ND |
|  | Maize | GRMZM2G025387_T01 | ND |
| BdCDPK15 | Arabidopsis | At5g19360 | ND |
|  | Rice | LOC_Os01g59360 | ND |
|  | Grape | VIT_206s0009g03150 | ND |
|  | Maize | GRMZM2G167276_T01 | ND |
| BdCDPK16 | Arabidopsis | At3g51850 | ND |
|  | Rice | LOC_Os01g61590 | ND |
|  | Grape | VIT_208s0105g00390 | ND |
|  | Maize | GRMZM2G311220_T01 | ND |
| BdCDPK17 | Arabidopsis | At5g66210 | GA[[14](#_ENREF_14)],JA[[15](#_ENREF_15)] |
|  | Rice | LOC_Os02g03410 | Salt, drought[[26](#_ENREF_26)] |
|  | Grape | VIT_204s0023g03420 | ND |
|  | Maize | GRMZM2G053868_T01 | ND |
| BdCDPK18 | Arabidopsis | At2g17290 | ABA[[18](#_ENREF_18)], salt/drought[[19](#_ENREF_19)] , MeJA[[20](#_ENREF_20)] |
|  | Rice | LOC_Os10g39420 | JA[[27](#_ENREF_27)] |
|  | Grape | VIT_203s0038g03960 | ND |
|  | Maize | GRMZM2G032852_T01 | ND |
| BdCDPK19 | Rice | LOC_Os08g42750 | Salt, ABA[[28](#_ENREF_28), [29](#_ENREF_29)] |
|  | Maize | GRMZM2G332660_T01 | ND |
| BdCDPK20 | Arabidopsis | At4g38230 | ND |
|  | Rice | LOC_Os02g46090 | ND |
|  | Grape | VIT_203s0038g03960 | ND |
|  | Maize | GRMZM2G081310_T01 | ND |
| BdCDPK21 | Arabidopsis | At2g17290 | ABA[[18](#_ENREF_18)], salt/drought[[19](#_ENREF_19)] , MeJA[[20](#_ENREF_20)] |
|  | Rice | LOC_Os02g58520 | Cold, dehydration, heat[[6](#_ENREF_6)] |
|  | Grape | VIT_203s0038g03960 | ND |
|  | Maize | GRMZM2G347047_T01 | ND |
| BdCDPK22 | Arabidopsis | At5g04870 | Salt/drought[[3](#_ENREF_3)] , SA[[4](#_ENREF_4)] |
|  | Rice | LOC_Os12g30150 | ABA, H_2_O_2_, NO, cold[[30](#_ENREF_30)] |
|  | Grape | VIT_213s0175g00080 | Drought[[2](#_ENREF_2)] |
|  | Maize | GRMZM2G353957_T01 | ND |
| BdCDPK23 | Arabidopsis | At4g09570 | Auxin[[23](#_ENREF_23)], ABA, drought and salt [[31](#_ENREF_31)] |
|  | Arabidopsis | At1g35670 | Drought and salt [[7](#_ENREF_7)], ABA[[31](#_ENREF_31)] |
|  | Rice | LOC_Os11g07040 | Cold[[32](#_ENREF_32)] |
|  | Grape | VIT_207s0130g00130 | ABA, drought[[2](#_ENREF_2)] |
|  | Maize | GRMZM2G035843_T01 | ABA[[33](#_ENREF_33)] |
| BdCDPK24 | Arabidopsis | At5g12180 | ND |
|  | Rice | LOC_Os11g04170 | Cold, dehydration, heat[[6](#_ENREF_6)] |
|  | Grape | VIT_206s0009g03150 | ND |
|  | Maize | GRMZM2G365815_T01 | ND |
| BdCDPK25 | Rice | LOC_Os08g42750 | Salt, ABA[[28](#_ENREF_28), [29](#_ENREF_29)] |
|  | Maize | GRMZM2G080871_T01 | ND |
| BdCDPK26 | Rice | LOC_Os12g12860 | ND |
|  | Maize | GRMZM2G097533_T01 | ND |
| BdCDPK27 | Arabidopsis | At1g35670 | Drought and salt [[7](#_ENREF_7)], ABA[[31](#_ENREF_31)] |
|  | Arabidopsis | At4g09570 | Auxin[[23](#_ENREF_23)], ABA, drought and salt [[31](#_ENREF_31)] |
|  | Rice | LOC_Os12g07230 | ND |
|  | Grape | VIT_207s0130g00130 | ABA, drought[[2](#_ENREF_2)] |
|  | Maize | GRMZM2G463464_T01 | ND |
| BdCDPK28 | Arabidopsis | At5g12180 | ND |
|  | Rice | LOC_Os12g03970 | ND |
|  | Maize | GRMZM2G365815_T01 | ND |
| BdCDPK29 | Arabidopsis | At1g50700 | ABA[[12](#_ENREF_12), [13](#_ENREF_13)] |
|  | Rice | LOC_Os04g47300 | Salt[[34](#_ENREF_34)] |
|  | Maize | GRMZM2G112057_T01 | ND |
| BdCDPK30 | Arabidopsis | At2g17290 | ABA[[18](#_ENREF_18)], salt/drought[[19](#_ENREF_19)] , MeJA[[20](#_ENREF_20)] |
|  | Rice | LOC_Os04g49510 | Cold, salt, drought[[6](#_ENREF_6), [35](#_ENREF_35)] |
|  | Grape | VIT_203s0038g03960 | ND |
|  | Maize | GRMZM2G314396_T01 | Cold[[36](#_ENREF_36)] |

a: ND, not determined; blue font indicates the negative function.

**Reference**

1. Zou JJ, Li XD, Ratnasekera D, Wang C, Liu WX, Song LF, Zhang WZ, Wu WH: **Arabidopsis CALCIUM-DEPENDENT PROTEIN KINASE8 and CATALASE3 Function in Abscisic Acid-Mediated Signaling and H2O2 Homeostasis in Stomatal Guard Cells under Drought Stress**. *The Plant cell* 2015, **27**(5):1445-1460.

2. Chen F, Fasoli M, Tornielli GB, Dal Santo S, Pezzotti M, Zhang L, Cai B, Cheng ZM: **The evolutionary history and diverse physiological roles of the grapevine calcium-dependent protein kinase gene family**. *PloS one* 2013, **8**(12):e80818.

3. Huang K, Peng L, Liu Y, Yao R, Liu Z, Li X, Yang Y, Wang J: **Arabidopsis calcium-dependent protein kinase AtCPK1 plays a positive role in salt/drought-stress response**. *Biochemical and biophysical research communications* 2018, **498**(1):92-98.

4. Coca M, San Segundo B: **AtCPK1 calcium-dependent protein kinase mediates pathogen resistance in Arabidopsis**. *The Plant journal : for cell and molecular biology* 2010, **63**(3):526-540.

5. Bundo M, Coca M: **Calcium-dependent protein kinase OsCPK10 mediates both drought tolerance and blast disease resistance in rice plants**. *Journal of experimental botany* 2017, **68**(11):2963-2975.

6. Wan B, Lin Y, Mou T: **Expression of rice Ca(2+)-dependent protein kinases (CDPKs) genes under different environmental stresses**. *FEBS letters* 2007, **581**(6):1179-1189.

7. Urao T, Katagiri T, Mizoguchi T, Yamaguchi-Shinozaki K, Hayashida N, Shinozaki K: **Two genes that encode Ca(2+)-dependent protein kinases are induced by drought and high-salt stresses in Arabidopsis thaliana**. *Molecular & general genetics : MGG* 1994, **244**(4):331-340.

8. Zou JJ, Wei FJ, Wang C, Wu JJ, Ratnasekera D, Liu WX, Wu WH: **Arabidopsis calcium-dependent protein kinase CPK10 functions in abscisic acid- and Ca2+-mediated stomatal regulation in response to drought stress**. *Plant physiology* 2010, **154**(3):1232-1243.

9. Yang L, Jin Y, Huang W, Sun Q, Liu F, Huang X: **Full-length transcriptome sequences of ephemeral plant Arabidopsis pumila provides insight into gene expression dynamics during continuous salt stress**. *BMC genomics* 2018, **19**(1):717.

10. Wei S, Hu W, Deng X, Zhang Y, Liu X, Zhao X, Luo Q, Jin Z, Li Y, Zhou S *et al*: **A rice calcium-dependent protein kinase OsCPK9 positively regulates drought stress tolerance and spikelet fertility**. *BMC plant biology* 2014, **14**:133.

11. Fu L, Yu, Xiangchun, An, Chengcai: **OsCPK20 positively regulates Arabidopsis resistance against Pseudomonas syringae pv. tomato and rice resistance against Magnaporthe grisea**. *Acta Physiologiae Plantarum* 2014, **36**(2):273-282.

12. Chen DH, Liu HP, Li CL: **Calcium-dependent protein kinase CPK9 negatively functions in stomatal abscisic acid signaling by regulating ion channel activity in Arabidopsis**. *Plant molecular biology* 2019, **99**(1-2):113-122.

13. Li CL, Wang M, Wu XM, Chen DH, Lv HJ, Shen JL, Qiao Z, Zhang W: **THI1, a Thiamine Thiazole Synthase, Interacts with Ca2+-Dependent Protein Kinase CPK33 and Modulates the S-Type Anion Channels and Stomatal Closure in Arabidopsis**. *Plant physiology* 2016, **170**(2):1090-1104.

14. Matschi S, Werner S, Schulze WX, Legen J, Hilger HH, Romeis T: **Function of calcium-dependent protein kinase CPK28 of Arabidopsis thaliana in plant stem elongation and vascular development**. *The Plant journal : for cell and molecular biology* 2013, **73**(6):883-896.

15. Matschi S, Hake K, Herde M, Hause B, Romeis T: **The calcium-dependent protein kinase CPK28 regulates development by inducing growth phase-specific, spatially restricted alterations in jasmonic acid levels independent of defense responses in Arabidopsis**. *The Plant cell* 2015, **27**(3):591-606.

16. Almadanim MC, Alexandre BM, Rosa MTG, Sapeta H, Leitao AE, Ramalho JC, Lam TT, Negrao S, Abreu IA, Oliveira MM: **Rice calcium-dependent protein kinase OsCPK17 targets plasma membrane intrinsic protein and sucrose-phosphate synthase and is required for a proper cold stress response**. *Plant, cell & environment* 2017, **40**(7):1197-1213.

17. Almadanim MC, Goncalves NM, Rosa MTG, Alexandre BM, Cordeiro AM, Rodrigues M, Saibo NJM, Soares CM, Romao CV, Oliveira MM *et al*: **The rice cold-responsive calcium-dependent protein kinase OsCPK17 is regulated by alternative splicing and post-translational modifications**. *Biochimica et biophysica acta Molecular cell research* 2018, **1865**(2):231-246.

18. Mori IC, Murata Y, Yang Y, Munemasa S, Wang YF, Andreoli S, Tiriac H, Alonso JM, Harper JF, Ecker JR *et al*: **CDPKs CPK6 and CPK3 function in ABA regulation of guard cell S-type anion- and Ca(2+)-permeable channels and stomatal closure**. *PLoS biology* 2006, **4**(10):e327.

19. Xu J, Tian YS, Peng RH, Xiong AS, Zhu B, Jin XF, Gao F, Fu XY, Hou XL, Yao QH: **AtCPK6, a functionally redundant and positive regulator involved in salt/drought stress tolerance in Arabidopsis**. *Planta* 2010, **231**(6):1251-1260.

20. Munemasa S, Hossain MA, Nakamura Y, Mori IC, Murata Y: **The Arabidopsis calcium-dependent protein kinase, CPK6, functions as a positive regulator of methyl jasmonate signaling in guard cells**. *Plant physiology* 2011, **155**(1):553-561.

21. Abbasi F, Onodera H, Toki S, Tanaka H, Komatsu S: **OsCDPK13, a calcium-dependent protein kinase gene from rice, is induced by cold and gibberellin in rice leaf sheath**. *Plant molecular biology* 2004, **55**(4):541-552.

22. Kakar KU, Ren XL, Nawaz Z, Cui ZQ, Li B, Xie GL, Hassan MA, Ali E, Sun GC: **A consortium of rhizobacterial strains and biochemical growth elicitors improve cold and drought stress tolerance in rice (Oryza sativa L.)**. *Plant biology* 2016, **18**(3):471-483.

23. Rietz S, Dermendjiev G, Oppermann E, Tafesse FG, Effendi Y, Holk A, Parker JE, Teige M, Scherer GF: **Roles of Arabidopsis patatin-related phospholipases a in root development are related to auxin responses and phosphate deficiency**. *Molecular plant* 2010, **3**(3):524-538.

24. Mehlmer N, Wurzinger B, Stael S, Hofmann-Rodrigues D, Csaszar E, Pfister B, Bayer R, Teige M: **The Ca(2+) -dependent protein kinase CPK3 is required for MAPK-independent salt-stress acclimation in Arabidopsis**. *The Plant journal : for cell and molecular biology* 2010, **63**(3):484-498.

25. Choi HI, Park HJ, Park JH, Kim S, Im MY, Seo HH, Kim YW, Hwang I, Kim SY: **Arabidopsis calcium-dependent protein kinase AtCPK32 interacts with ABF4, a transcriptional regulator of abscisic acid-responsive gene expression, and modulates its activity**. *Plant physiology* 2005, **139**(4):1750-1761.

26. Campo S, Baldrich P, Messeguer J, Lalanne E, Coca M, San Segundo B: **Overexpression of a Calcium-Dependent Protein Kinase Confers Salt and Drought Tolerance in Rice by Preventing Membrane Lipid Peroxidation**. *Plant physiology* 2014, **165**(2):688-704.

27. Breviario D, Morello L, Giani S: **Molecular cloning of two novel rice cDNA sequences encoding putative calcium-dependent protein kinases**. *Plant molecular biology* 1995, **27**(5):953-967.

28. Asano T, Hakata M, Nakamura H, Aoki N, Komatsu S, Ichikawa H, Hirochika H, Ohsugi R: **Functional characterisation of OsCPK21, a calcium-dependent protein kinase that confers salt tolerance in rice**. *Plant molecular biology* 2011, **75**(1-2):179-191.

29. Chen Y, Zhou X, Chang S, Chu Z, Wang H, Han S, Wang Y: **Calcium-dependent protein kinase 21 phosphorylates 14-3-3 proteins in response to ABA signaling and salt stress in rice**. *Biochemical and biophysical research communications* 2017, **493**(4):1450-1456.

30. Lv X, Li H, Chen X, Xiang X, Guo Z, Yu J, Zhou Y: **The role of calcium-dependent protein kinase in hydrogen peroxide, nitric oxide and ABA-dependent cold acclimation**. *Journal of experimental botany* 2018, **69**(16):4127-4139.

31. Zhu SY, Yu XC, Wang XJ, Zhao R, Li Y, Fan RC, Shang Y, Du SY, Wang XF, Wu FQ *et al*: **Two calcium-dependent protein kinases, CPK4 and CPK11, regulate abscisic acid signal transduction in Arabidopsis**. *The Plant cell* 2007, **19**(10):3019-3036.

32. Liu Y, Xu C, Zhu Y, Zhang L, Chen T, Zhou F, Chen H, Lin Y: **The calcium-dependent kinase OsCPK24 functions in cold stress responses in rice**. *Journal of integrative plant biology* 2018, **60**(2):173-188.

33. Kong X, Lv W, Jiang S, Zhang D, Cai G, Pan J, Li D: **Genome-wide identification and expression analysis of calcium-dependent protein kinase in maize**. *BMC genomics* 2013, **14**:433.

34. Asano T, Hayashi N, Kobayashi M, Aoki N, Miyao A, Mitsuhara I, Ichikawa H, Komatsu S, Hirochika H, Kikuchi S *et al*: **A rice calcium-dependent protein kinase OsCPK12 oppositely modulates salt-stress tolerance and blast disease resistance**. *The Plant journal : for cell and molecular biology* 2012, **69**(1):26-36.

35. Saijo Y, Hata S, Kyozuka J, Shimamoto K, Izui K: **Over-expression of a single Ca2+-dependent protein kinase confers both cold and salt/drought tolerance on rice plants**. *The Plant journal : for cell and molecular biology* 2000, **23**(3):319-327.

36. Weckwerth P, Ehlert B, Romeis T: **ZmCPK1, a calcium-independent kinase member of the Zea mays CDPK gene family, functions as a negative regulator in cold stress signalling**. *Plant, cell & environment* 2015, **38**(3):544-558.
